# Does alcohol use and related harm differ based on the age of initiation to alcohol? Results from a prospective cohort study

**DOI:** 10.1111/add.70183

**Published:** 2025-10-13

**Authors:** Philip J. Clare, Wing See Yuen, Alexandra Henderson, Kypros Kypri, Raimondo Bruno, Tim Slade, Delyse Hutchinson, Nyanda McBride, Monika Wodalowski, Jim McCambridge, Louisa Degenhardt, Veronica C. Boland, Richard P. Mattick, Amy Peacock

**Affiliations:** ^1^ Prevention Research Collaboration The University of Sydney Sydney New South Wales Australia; ^2^ National Drug and Alcohol Research Centre University of New South Wales Randwick New South Wales Australia; ^3^ Charles Perkins Centre The University of Sydney Sydney New South Wales Australia; ^4^ School of Medicine and Public Health University of Newcastle Newcastle New South Wales Australia; ^5^ School of Psychological Sciences University of Tasmania Dynnyrne Tasmania Australia; ^6^ The Matilda Centre for Research in Mental Health and Substance Use The University of Sydney Sydney New South Wales Australia; ^7^ SEED Lifespan Strategic Research Centre, School of Psychology, Faculty of Health Deakin University Burwood Victoria Australia; ^8^ Department of Paediatrics The University of Melbourne Melbourne Victoria Australia; ^9^ Murdoch Children's Research Institute, Royal Children's Hospital The University of Melbourne Melbourne Victoria Australia; ^10^ National Drug Research Institute Curtin University Bentley Western Australia Australia; ^11^ enAble Institute Curtin University Bentley Western Australia Australia; ^12^ Research and Innovation Portfolio The University of Sydney Sydney New South Wales Australia; ^13^ UCL School of Pharmacy University College London London UK

**Keywords:** adolescence, alcohol, alcohol‐related harm, initiation, prospective cohort, risky drinking

## Abstract

**Background and Aims:**

There is evidence to suggest that earlier initiation to alcohol increases the rate and severity of alcohol consumption. However, this often overlooks the fact that earlier initiation also means a longer drinking history. This paper estimated differences in patterns of alcohol use and harm across adolescence and early adulthood, allowing for the fact that the patterns over time may differ depending on the age at which initiation occurred.

**Design:**

Prospective cohort study.

**Setting:**

Australia.

**Participants:**

The Australian Parental Supply of Alcohol Longitudinal Study (APSALS), a cohort of *n* = 1906 adolescents recruited in adolescence (mean age 12.9) from 107 Australian schools and followed up until adulthood (11 annual waves total, 2010–2021).

**Measurements:**

We defined age of initiation as the age at which alcohol consumption was first reported in the study. Our outcomes were amount of alcohol consumed, monthly heavy episodic drinking, alcohol‐related harm and self‐reported symptoms of alcohol use disorder in the years following initiation.

**Findings:**

Those who initiated at age 12 had a lower risk of consumption [risk ratio (RR) 0.01; 95% confidence interval (CI) = 0.01–0.02] in the year following initiation (age 13), compared with those who initiated at age 18. Similarly, those who initiated at age 15 had a lower risk of alcohol use disorder in the year after initiation (RR 0.66; 95% CI = 0.52–0.83), compared with those who initiated at age 18. However, at age 20, those who initiated at age 12 showed higher consumption (RR 1.57; 95% CI = 1.18–2.09), monthly heavy episodic drinking (RR 1.24; 95% CI = 1.02–1.51) and alcohol‐related harms [incidence‐rate ratio (IRR) 1.73; 95% CI = 1.21–2.46] than those who initiated at age 18. Similar results were seen for symptoms consistent with DSM‐IV alcohol dependence (RR 1.20; 95% CI = 1.05–1.38), DSM‐IV alcohol abuse (RR 1.54; 95% CI = 1.04–2.29) and DSM‐5 alcohol use disorder (RR 1.36; 95% CI = 1.12–1.65). However, there was evidence of ageing out, with risk of heavy episodic drinking and alcohol‐related harm peaking around age 20 and then declining, regardless of when initiation occurred.

**Conclusions:**

Later initiation to alcohol appears to be associated with more rapid escalation in drinking and related harm, but lower ‘peak’ harm than earlier initiation. The findings support the current guidelines recommending adolescents avoid alcohol until adulthood, and reinforce the need for public health intervention targeting both children and parents.

## INTRODUCTION

Alcohol is a leading cause of preventable death and disability worldwide and is the leading cause of burden of disease in people aged 10–24 years [1]. There is some evidence to suggest that an earlier initiation to alcohol increases both the rate and the severity of alcohol consumption [2, 3, 4], and leads to a greater risk of alcohol dependence and disorder [5, 6, 7, 8, 9, 10]. However, there is other evidence to show that the progression of drinking behaviours is slower among those who initiate earlier [11, 12]. Further, most studies that have shown links between earlier initiation and subsequent harm measured outcomes at a fixed point in time (e.g. a final study wave), meaning that those who initiated earlier had been consuming alcohol for longer by the time the outcome was ascertained [2, 3, 7, 8, 9, 10]. This makes it unclear whether a later initiation to alcohol actually reduces the risk of harm or merely delays it.

While the evidence on the effects of earlier initiation to alcohol is mixed and somewhat limited, many jurisdictions have a minimum legal age for the purchase of alcohol nonetheless, and national alcohol guidelines in Australia, Canada, the UK and the USA all recommend avoiding alcohol during childhood [13, 14, 15, 16]. Delaying alcohol initiation specifically until the minimum legal age for purchasing may reduce the negative health impacts of alcohol consumption [17]. Norberg *et al*. [18] found that an earlier legal purchase age was linked to greater risk of substance use problems, and when legal age of purchase was accounted for, the age of initiation was not important. This suggests that lower legal purchase age may be a bigger driver of harm than earlier initiation; however, it does not account for potential differences in patterns of alcohol use and related harm in the years following initiation. Despite this, the average age of alcohol initiation in Australia in 2019 was 16.2 years for males and 16.3 years for females [19], below the minimum legal purchase age of 18 years, with a similar average age of initiation reported in countries such as the USA [20] and Canada [21].

However, it has also been suggested that young people who wait until reaching the legal age may then show a rapid escalation in alcohol use owing to a lack of experience in contexts in which alcohol is consumed, which could in turn lead to increased risk of harm [11]. To date, there has been very limited research comparing outcomes for those initiating prior to and those initiating after reaching the legal age of purchase. Morleo *et al*. [22] found that those who drank at least monthly prior to reaching the legal age (age 18 years) were more likely to report lifetime serious alcohol‐related problems than those who did not drink regularly before 18 years of age. However, this could overstate the risk of harm if the earlier initiation of infrequent alcohol consumption of smaller quantities (such as the infrequent supply of sips by parents) reduces the likelihood of progressing from alcohol initiation to regular (i.e. monthly) consumption. That is, both the age of initiation and the amount consumed early after initiation may be important.

This study used data from the Australian Parental Supply of Alcohol Longitudinal Study (APSALS) cohort to estimate whether there are differences in alcohol‐related outcomes in the years following alcohol initiation, based on the age at which initiation occurred. More specifically, we hypothesised that:

1. those who initiate earlier in age will report greater consumption and harm at age 20 years, 2 years after reaching the legal age of purchase, compared with those who initiated later;
2. those who initiated alcohol consumption later in adolescence (age 16–18 years) would report a more rapidly increasing pattern of consumption and harm; and
3. those who initiate at age 18 years will show both a more rapid increase and greater peak consumption than those who initiate at other ages.

## METHODS

The study and its hypotheses were pre‐registered on the Open Science Framework (https://doi.org/10.17605/OSF.IO/BRDUV). The study is reported in line with the STROBE (STrengthening the Reporting of OBservational studies in Epidemiology) guidelines [23]. The STROBE reporting checklist is included in Table A1.

### Sample

We used data from the APSALS cohort (ClinicalTrials.gov: NCT02280551), a longitudinal cohort of adolescents and their parents, recruited from a convenience sample of 107 grade 7 cohorts in Australian schools, with adolescents recruited at a mean age of 12.9 years and followed up annually for 10 years (with 11 waves in total) [24, 25]. A recruitment flow diagram of the APSALS cohort is shown in Figure A1. Participants were excluded if they reported initiating alcohol more than 1 year prior to the commencement of the study, or if they had not initiated alcohol by 20 years of age. Around half of the participants initiated alcohol during the course of the study and up to age 20 years, resulting in a study sample of *n* = 928. All available waves were included such that longer patterns were available for those who initiated alcohol earlier in the study, but outcomes were available for at least the 3 years following initiation for all ages of initiation.

### Exposure

We defined the exposure as the age at which participants initiated drinking alcohol (any amount). At wave 1 (age 12.9 years) participants were asked the age at which they first consumed alcohol. In subsequent waves, we defined initiation based on the age at which alcohol consumption was first reported. Because the survey asked about consumption in the past year, initiation may have occurred up to a year prior to the age at the time of survey completion. As such, we defined the time of initiation as 6 months prior to completion of the survey.

As a secondary analysis, we also looked at the initiation of whole drinks (excluding sips of alcohol).

### Outcomes

We considered six primary outcomes: (i) alcohol consumption (frequency × quantity); (ii) heavy episodic drinking (HED); (iii) alcohol‐related harms; (iv) symptoms of Diagnostic and Statistical Manual of Mental Disorders, Fourth Edition (DSM‐IV) alcohol dependence; (v) symptoms of DSM‐IV alcohol abuse; and (vi) symptoms of DSM‐5 alcohol use disorder (AUD). Each was assessed based on the past year at each survey wave.

#### Frequency and quantity of alcohol consumption

Participants were asked how often they consumed alcohol in the past year (‘never’, ‘less than once a month’, ‘about 1/month’, ‘2–3 days/month’, ‘1–2 days/week’, ‘3–4 days/week’, ‘5–6 days/week’, ‘every day’), and how many standard drinks (equivalent to 10 g of alcohol) [26] they typically consumed when they drank alcohol (‘sip or taste’, ‘1–2 drinks’, ‘3–4 drinks’, ‘5–6 drinks’, ‘7–10 drinks’, ‘11–12 drinks’, ‘13 or more drinks’). These two variables were mid‐point coded (e.g. 3–4 days/week was coded as 3.5*52 = 182 days per year; 3–4 drinks was coded as 3.5), and then multiplied together to provide an estimate of the number of standard drinks consumed in the past year.

#### Heavy episodic drinking

Participants were asked how often in the past year they consumed at least four standard drinks on an occasion, consistent with Australian government guidelines on alcohol consumption [27]. As there is arguably more risk associated with regular HED compared with very occasional HED, we defined our outcome as HED that occurred at least monthly. An additional sensitivity analysis also considered any HED in the past year.

#### Experience of alcohol‐related harm

Adolescents were asked to report how often they experienced each of 14 alcohol‐related harms, including hangovers and getting in trouble with friends or parents, in the past year, (‘never’, ‘once’, ‘twice’ ‘3–4 times’, ‘5–11 times’, ‘12+ times’) [28]. The full list of harms included in the scale can be seen in Table B1. The primary variable was the number of different harms experienced (range: 0–14). As a sensitivity analysis, we also analysed a binary variable based on experiencing any (or none) of the 14 harms.

#### DSM‐IV dependence, DSM‐IV abuse and DSM‐5 AUD

From wave 5 (age 16.8 years) onwards, participants were assessed using the Diagnostic Interview Schedule for Children IV (DISC‐IV) [29], a scale that covers the symptoms of DSM‐IV alcohol abuse, DSM‐IV alcohol dependence and DSM‐5 AUD in the past 12 months. While past studies have shown high concordance between DSM‐IV and DSM‐5 diagnoses of AUD [30, 31], there are differences in the way that they classify disorder. To allow comparisons with older studies conducted prior to the introduction of DSM‐5, and to look at differences arising from the assessment of disorders, we included both DSM‐IV and DSM‐5 outcomes. For each of the three, we coded a binary variable (no/yes) indicating whether the participant reported sufficient symptoms to meet a diagnosis for that condition (at least three of the seven symptoms for dependence; at least one of the four symptoms for abuse; and at least two of the 11 symptoms for AUD). Based on DSM‐IV criteria, participants were only coded as self‐reporting DSM‐IV alcohol abuse if they did not meet the criteria for DSM‐IV alcohol dependence.

### Covariates

Analysis controlled for a range of covariates, based on a literature review of factors linked with early alcohol consumption [24]. These included child [sex; having money to buy alcohol; and externalising, anxious/depressed, withdrawn/depressed and social problems, as defined by the Child Behavior Checklist (CBCL)], peer (peer substance use; peer disapproval of substance use), parent (parental alcohol consumption; parental monitoring; parental consistency; parental demandingness; parental responsiveness; parent born in Australia; parent education; parent employment status; parent religiousness) and family (household income; single‐parent household; alcohol‐specific rules; home access to alcohol; family conflict; family positive relations; area‐level socio‐economic status) covariates. All covariates were measured at APSALS wave 1, and were considered time constant. Further details on the covariates are included in Appendix B.

### Statistical analysis

We report descriptive statistics at the time of initiation, with continuous variables reported as means and standard deviations for normally distributed variables, medians and inter‐quartile ranges (IQRs) for counts or skewed variables, and categorical variables reported as numbers and percentages.

Our primary analyses modelled the change over time in each outcome using a mixed‐effects logistic regression model, with fixed effects of time (varying) and age of initiation (constant), and with a random intercept to account for the fact that the sample includes repeated measures of each participant. To standardise analyses so that any change over time reflects change since initiation, we recoded all participants so that ‘Time 0’ was the wave in which initiation occurred. This allowed us to compare early patterns in the outcomes between different ages of initiation. Results are reported as the marginal predicted probability of the outcome. We used a critical *P*‐value of *P* < 0.05, and all results of inferential statistics are presented with 95% confidence intervals. Analysis was conducted in R 4.3.0 (R Foundation for Statistical Computing, Vienna, Austria) [32] and Stata 18.0 (StataCorp LLC, College Station, TX, USA) [33]. Analysis code is available at https://www.philipclare.com/code/apsals/.

To allow the patterns to differ by age, we included a time by age of initiation interaction term. We considered a range of non‐linear terms of both time and age of initiation, including polynomial and logarithmic terms, with the best model selected based on the Akaike information criterion (AIC) and Bayesian information criterion (BIC). Where the AIC and BIC differed, the model suggested by AIC was selected to prioritise the accuracy of model predictions, as results are based on marginal predicted means not the parameters of the model itself.

As a *post hoc* analysis, we also examined predictors of the age at which initiation of sips and whole drinking occured, using linear regression of all predictors from the time of initiation on age of initiation of sips and age of initiation of whole drinks, to examine whether there are socio‐demographic differences in those who initiate alcohol earlier.

### Missing data

Because the sample is a longitudinal cohort, not all participants completed every wave of the study, and some participants refused to answer specific questions within survey waves. To reduce the chance of bias arising from missing data, we conducted the primary analysis using multiple imputation using chained equations in the R package ‘mice’, with models fit using random forests to allow for non‐linearity in the imputation models. More information on missing data procedures is included in Appendix C, Figures C1 and C2.

## RESULTS

### Sample

Alcohol initiation occurred at a median age of 15.3 years (IQR 13.4–17.4 years). Further details of the sample are included in Table 1.

**TABLE 1:** Characteristics of the sample (*n* = 928).

		Mean (SD)/median (IQR)/*n* (%)
Age of initiation, years^a^		15.3 (13.4–17.4)
Sex	Male	459 (49.5%)
	Female	469 (50.5%)
Has money to buy alcohol	No	533 (57.4%)
	Yes	394 (42.5%)
Externalising^b^		42.3 (10.1)
Anxious/depressed^b^		53.4 (6.3)
Withdrawn/depressed^b^		52.2 (4.8)
Home access to alcohol		15.4 (3.7)
Family conflict^a^		0 (0–1.00)
Family positive relations^a^		3.00 (3.00–3.00)
Household income	Up to $34,000	77 (8.3%)
	$35–80,000	222 (23.9%)
	$81–180,000	455 (49.0%)
	>$180,000	167 (18.0%)
Area‐level socio‐economic status (SEIFA IRSAD)^c^	Lowest tertile	156 (16.8%)
	Middle tertile	206 (22.2%)
	Highest tertile	558 (60.1%)
Parent alcohol use^a^		105 (18.0–273.0)
Parental monitoring		28.1 (2.83)
Parental demandingness		24.0 (3.62)
Parental responsiveness		30.1 (4.08)
Parent born in Australia	No	242 (26.1%)
	Yes	678 (73.1%)
Parent education	High school or less	297 (32.0%)
	Diploma/Trade/Non‐trade	295 (31.8%)
	University	326 (35.1%)
Parent employment	Employed	744 (80.2%)
	Unemployed – in workforce	115 (12.4%)
	Unemployed – not in workforce	61 (6.6%)
Parent religiousness	Not religious/a little religious	632 (68.1%)
Peer substance use^a^		1.00 (0–2.00)
Peer disapproval of substance use^a^		8.00 (7.00–8.00)

^a^ Reported as median (IQR) owing to non‐normality.

^b^ Factors described by the Child Behavior Checklist (CBCL).

^c^ IRSAD = Index of Relative Socio‐Economic Advantage and Disadvantage; SEIFA = Socio‐Economic Indexes for Areas.

*Post hoc* analysis of the association between covariates at the time of initiation and age of initiation only showed evidence of an association with alcohol‐specific rules and perceptions of peer substance use (Appendix D, Table D1).

### Missing data

Around 40% of cases had at least some missing data, although there was only around 6% missing information. As such, all analyses reported are based on multiply imputed data with *m* = 20 imputations. More information on the amount and patterns of missing data is included in Appendix C.

### Alcohol consumption

**FIGURE 1:**
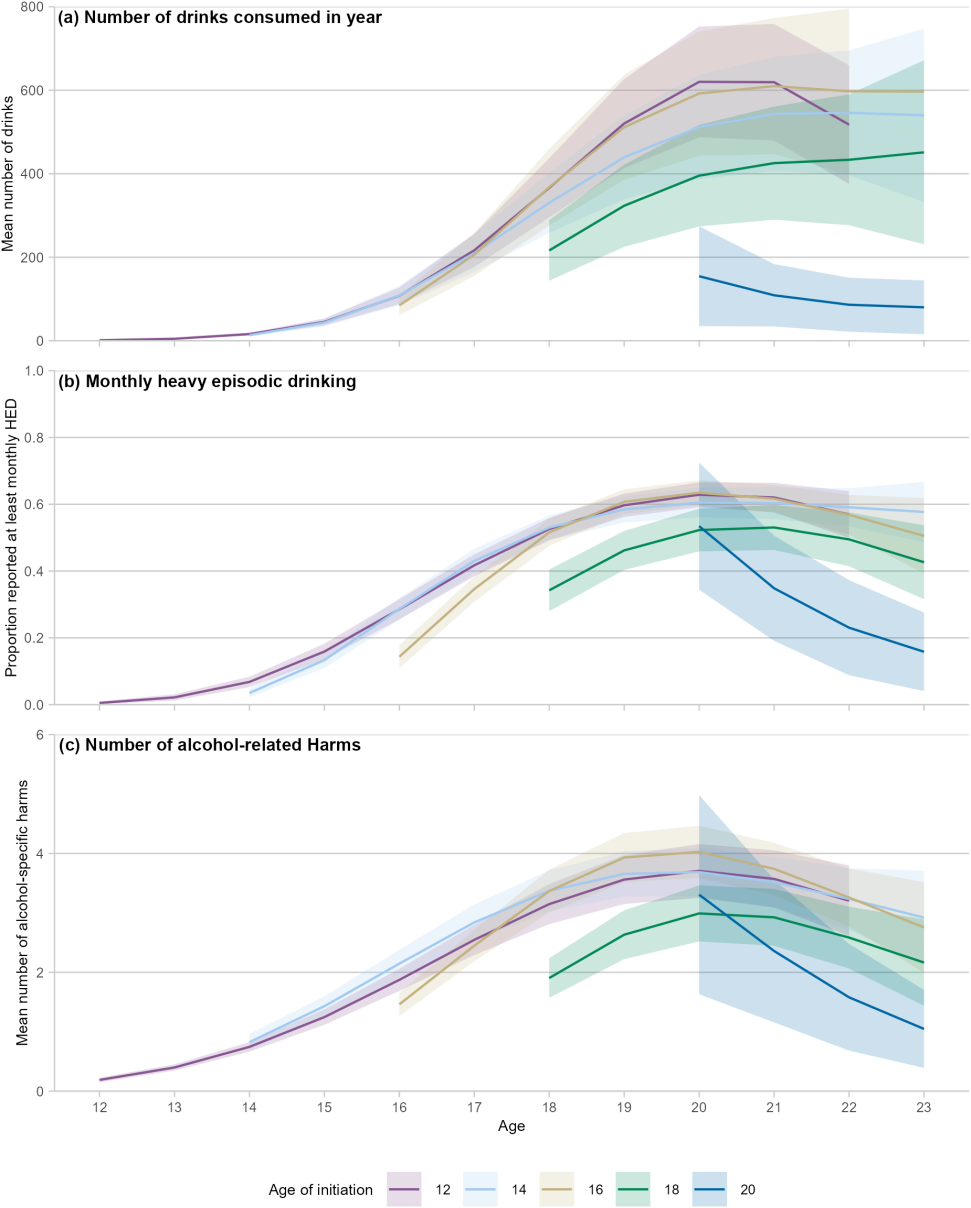
Patterns of alcohol consumption, heavy episodic drinking and alcohol‐related harms, by age of initiation. Note: ribbons show 95% confidence intervals around the trend. Full results for this figure are included in Tables E2, E3 and E4. Models of alcohol consumption and heavy episodic drinking (HED) included: linear and cubic terms for age; and linear, quadratic and cubic terms for time (age + age^3^ + time + time^2^ + time^3^). Model of alcohol‐related harms included: linear, quadratic and cubic terms for age; and linear, quadratic and cubic terms for time (age + age^2^ + age^3^ + time + time^2^ + time^3^). Further information on model fitting is included in Table E1.

Older ages of initiation were associated with a more rapid increase in the amount of alcohol consumed early in the years following initiation (Figure E1 and Tables 2 and E2), but with lower peak consumption in early adulthood. Those who initiated at age 12 years (RR = 0.01, 95% CI = 0.01–0.02) showed much lower risk of consumption in the year following initiation than those who initiated at age 18 years, with similar levels of risk seen among those initiating at ages 11 and 13 years. However, those who initiated any alcohol at age 12 years drank more at age 18 years [incidence‐rate ratio (IRR) 1.70, 95% CI = 1.25–2.30] and at age 20 years (IRR 1.57; 95% CI 1.18, 2.09) (Figures 1 and E2; Tables 3 and 4). Still, there was some evidence to suggest that alcohol consumption among those who initiated in early adolescence had peaked by age 20–22 years and begun to decline, while those who initiated from ages 14–19 years continued to show increases up to the end of the study period (age 23 years).

**TABLE 2:** Comparison of outcomes 1 year after initiation, based on age of initiation.

Age of initiation	RR/IRR (95% CI)					
	Number of drinks^a^	At least monthly HED	Number of harms^a^	DSM‐IV dependence	DSM‐IV abuse	DSM‐5 AUD^b^
11 years	0.00 (0.00–0.01)	0.06 (0.00–16.06)	0.04 (0.00–24.03)			
12 years	0.01 (0.01–0.02)	0.15 (0.00–6.13)	0.11 (0.00–9.28)			
13 years	0.05 (0.03–0.06)	0.32 (0.03–2.99)	0.24 (0.01–4.35)			
14 years	0.14 (0.10–0.18)	0.54 (0.16–1.80)	0.42 (0.07–2.46)			
15 years	0.33 (0.25–0.43)	0.77 (0.45–1.30)	0.64 (0.25–1.65)	0.51 (0.14–1.90)	0.67 (0.30–1.51)	0.66 (0.52–0.83)
16 years	0.64 (0.51–0.80)	0.93 (0.78–1.10)	0.84 (0.55–1.28)	0.75 (0.42–1.33)	0.85 (0.59–1.21)	0.84 (0.70–1.00)
17 years	0.94 (0.82–1.08)	1.00 (0.94–1.07)	0.97 (0.84–1.12)	0.93 (0.79–1.09)	0.97 (0.87–1.08)	0.96 (0.87–1.06)
18 years	Ref.	Ref.	Ref.	Ref.	Ref.	Ref.
19 years	0.72 (0.59–0.89)	0.95 (0.81–1.11)	0.92 (0.73–1.17)	0.95 (0.82–1.09)	0.94 (0.79–1.12)	0.95 (0.83–1.10)
20 years	0.33 (0.20–0.55)	0.89 (0.60–1.31)	0.75 (0.38–1.50)	0.75 (0.41–1.38)	0.80 (0.47–1.35)	0.83 (0.59–1.17)

^a^ Results are reported as incidence‐rate ratios (IRRs). All other results are reported as risk ratios (RRs). Models of alcohol consumption, heavy episodic drinking (HED) and Diagnostic and Statistical Manual of Mental Disorders (DSM) outcomes included: linear and cubic terms for age; and linear, quadratic and cubic terms for time (age + age^3^ + time + time^2^ + time^3^). Model of alcohol‐related harms included: linear, quadratic and cubic terms for age; and linear, quadratic and cubic terms for time (age + age^2^ + age^3^ + time + time^2^ + time^3^). Further information on model fitting is included in Table E1.

^b^ AUD = alcohol use disorder.

**TABLE 3:** Comparison of outcomes at age 18 years, based on age of initiation.

Age of initiation	RR/IRR (95% CI)					
	Number of drinks^a^	At least monthly HED	Number of harms^a^	DSM‐IV dependence	DSM‐IV abuse	DSM‐5 AUD^b^
11 years	2.37 (1.70–3.32)	1.68 (1.37–2.06)	1.78 (1.45–2.18)	2.65 (1.64–4.29)	2.34 (1.35–4.06)	2.21 (1.63–3.01)
12 years	1.70 (1.25–2.30)	1.54 (1.26–1.87)	1.65 (1.34–2.04)	2.46 (1.53–3.96)	2.08 (1.23–3.52)	2.13 (1.58–2.89)
13 years	1.47 (1.09–1.98)	1.50 (1.23–1.82)	1.65 (1.33–2.04)	2.52 (1.56–4.06)	2.21 (1.30–3.75)	2.18 (1.61–2.94)
14 years	1.53 (1.14–2.06)	1.55 (1.27–1.88)	1.77 (1.45–2.17)	2.65 (1.65–4.25)	2.54 (1.50–4.29)	2.24 (1.67–3.02)
15 years	1.69 (1.26–2.27)	1.59 (1.31–1.93)	1.88 (1.56–2.28)	2.52 (1.57–4.05)	2.60 (1.54–4.38)	2.20 (1.64–2.94)
16 years	1.70 (1.29–2.25)	1.51 (1.25–1.81)	1.77 (1.48–2.11)	2.00 (1.26–3.16)	2.06 (1.24–3.42)	1.93 (1.45–2.57)
17 years	1.41 (1.13–1.76)	1.25 (1.07–1.46)	1.39 (1.20–1.60)	1.33 (0.91–1.94)	1.29 (0.85–1.95)	1.47 (1.16–1.87)
18 years	Ref.	Ref.	Ref.	Ref.	Ref.	Ref.

^a^ Results are reported as incidence‐rate ratios (IRRs). All other results are reported as risk ratios (RRs). Models of alcohol consumption, heavy episodic drinking (HED) and Diagnostic and Statistical Manual of Mental Disorders (DSM) outcomes included: linear and cubic terms for age; and linear, quadratic and cubic terms for time (age + age^3^ + time + time^2^ + time^3^). Model of alcohol‐related harms included: linear, quadratic and cubic terms for age; and linear, quadratic and cubic terms for time (age + age^2^ + age^3^ + time + time^2^ + time^3^). Further information on model fitting is included in Table E1.

^b^ AUD = alcohol use disorder.

**TABLE 4:** Comparison of outcomes at age 20 years, based on age of initiation.

Age of initiation	RR/IRR (95% CI)					
	Number of drinks^a^	At least monthly HED	Number of harms^a^	DSM‐IV dependence	DSM‐IV abuse	DSM‐5 AUD^b^
11 years	1.83 (1.34–2.51)	1.15 (0.95–1.40)	1.73 (1.20–2.51)	1.19 (1.03–1.37)	1.59 (1.07–2.38)	1.35 (1.10–1.66)
12 years	1.57 (1.18–2.09)	1.24 (1.02–1.51)	1.73 (1.21–2.46)	1.20 (1.05–1.38)	1.54 (1.04–2.29)	1.36 (1.12–1.65)
13 years	1.34 (1.00–1.79)	1.21 (0.99–1.48)	1.65 (1.17–2.33)	1.17 (1.02–1.33)	1.38 (0.92–2.05)	1.34 (1.10–1.63)
14 years	1.30 (0.96–1.74)	1.23 (1.02–1.49)	1.67 (1.19–2.36)	1.16 (1.01–1.33)	1.42 (0.95–2.13)	1.34 (1.10–1.63)
15 years	1.40 (1.04–1.87)	1.31 (1.09–1.57)	1.74 (1.24–2.44)	1.19 (1.04–1.36)	1.63 (1.10–2.40)	1.35 (1.11–1.64)
16 years	1.50 (1.16–1.94)	1.35 (1.15–1.58)	1.69 (1.24–2.30)	1.21 (1.08–1.37)	1.73 (1.22–2.44)	1.31 (1.10–1.56)
17 years	1.39 (1.17–1.64)	1.24 (1.10–1.38)	1.40 (1.13–1.73)	1.16 (1.06–1.27)	1.47 (1.17–1.85)	1.19 (1.06–1.34)
18 years	Ref.	Ref.	Ref.	Ref.	Ref.	Ref.
19 years	0.59 (0.47–0.74)	0.84 (0.70–0.99)	0.79 (0.59–1.07)	0.84 (0.73–0.96)	0.76 (0.56–1.05)	0.83 (0.69–0.99)
20 years	0.39 (0.19–0.79)	1.11 (0.67–1.83)	1.29 (0.53–3.12)	1.02 (0.72–1.46)	1.62 (0.65–4.03)	0.83 (0.46–1.52)

^a^ Results are reported as incidence‐rate ratios (IRRs). All other results are reported as risk ratios (RRs). Models of alcohol consumption, heavy episodic drinking (HED) and Diagnostic and Statistical Manual of Mental Disorders (DSM) outcomes included: linear and cubic terms for age; and linear, quadratic and cubic terms for time (age + age^3^ + time + time^2^ + time^3^). Model of alcohol‐related harms included: linear, quadratic and cubic terms for age; and linear, quadratic and cubic terms for time (age + age^2^ + age^3^ + time + time^2^ + time^3^). Further information on model fitting is included in Table E1.

^b^ AUD = alcohol use disorder.

**FIGURE 2:**
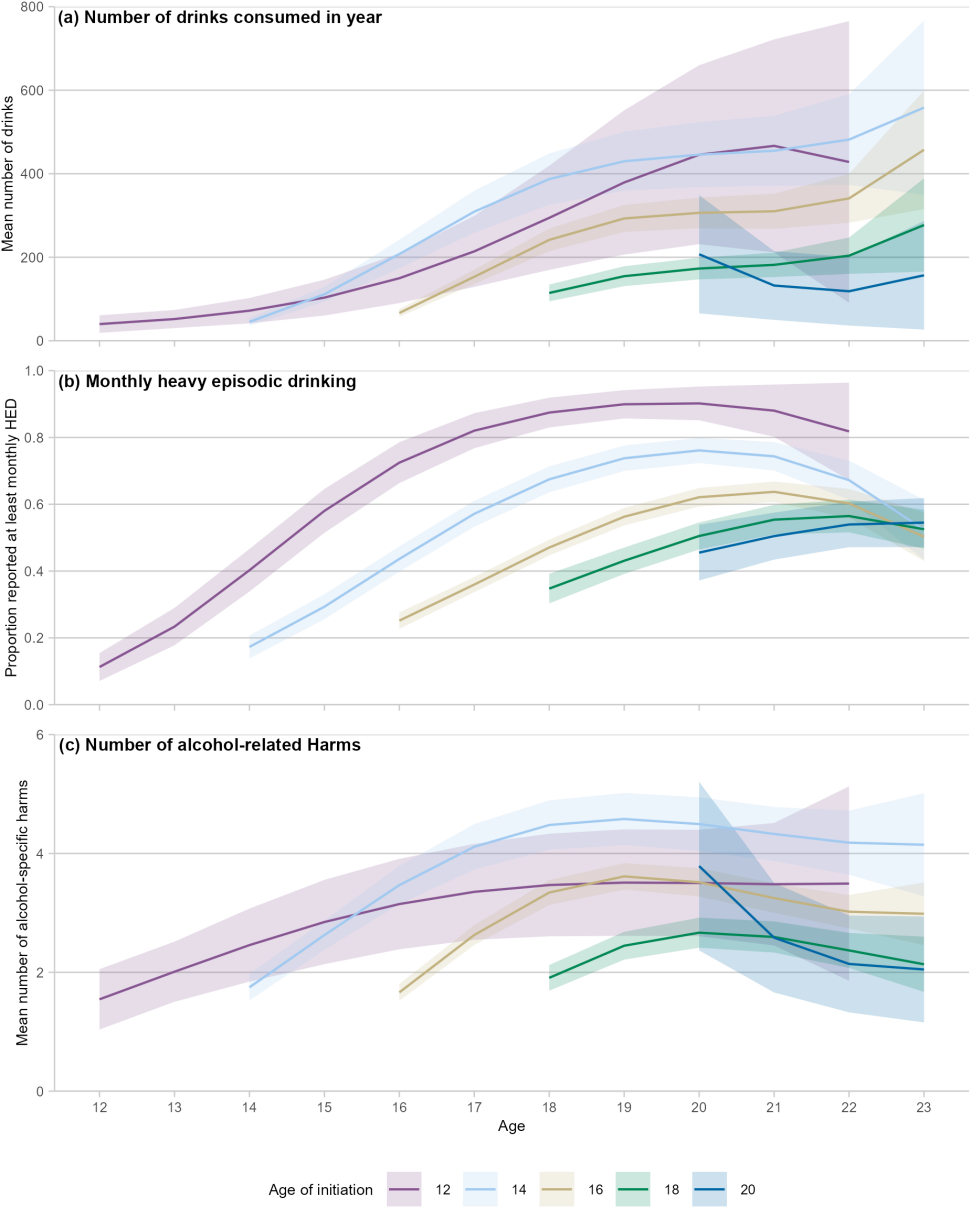
Patterns of alcohol consumption, heavy episodic drinking and alcohol‐related harms, by age of initiation to whole drinks. Note: ribbons show 95% confidence intervals around the trend. Full results for the figure are included in Tables F4, F5, and F6. Models of alcohol consumption and heavy episodic drinking (HED) included: linear and cubic terms for age; and linear, quadratic and cubic terms for time (age + age^3^ + time + time^2^ + time^3^). Model of alcohol‐related harms included: linear, quadratic and cubic terms for age; and linear, quadratic and cubic terms for time (age + age^2^ + age^3^ + time + time^2^ + time^3^). Further information on model fitting is included in Table F1.

Those who initiated whole drinks early showed similarly higher rates of drinking at age 18 years (age 12 years, IRR = 3.25, 95% CI = 2.45–4.31) and at age 20 years (age 12 years, IRR = 2.67, 95% CI 1.98–3.60), compared with those who initiated at age 18 years. However, there was less evidence of peaking (Figures 2, F1, and F2; Tables F2 and F3).

### Heavy episodic drinking

An earlier initiation of alcohol was associated with more HED in early adulthood, compared with young people who initiated later (Figure E3; Tables 2 and E3). A greater proportion of those who initiated up to age 16 years reported monthly HED at age 20 years, compared with those who initiated at age 18 years (Figures 1 and E4). However, the results of the monthly HED analysis showed evidence of peaking declining across the board by age 23 years.

Initiation to whole drinks showed a similar pattern, but with much greater differences between earlier ages of initiation, with initiation at each age showing a greater risk of HED than initiation 2 years later, as seen in Figures 2, F3 and F4. Sensitivity analyses of any HED in the past year were consistent with the primary analysis (Figures G1, G2, G5 and G6; Tables G1 and G3).

### Alcohol‐related harm

Consistent with the analysis of HED, an earlier initiation of alcohol was also associated with more harms experienced in early adulthood than in those who initiated later (Figures 1, E5 and E6; Tables 2 and E4). Experience of alcohol‐related harms also showed evidence of peaking, with declines evident by the age of 23 years, regardless of the age of initiation.

The results of the analysis of initiation of whole drinks showed a different pattern, with those who initiated at age 12 or 13 years showing lower reported levels of harms across adolescence than those who initiated whole drink consumption at age 14 years, although both showed higher rates of harm than initiation at later ages (Figures 2, F5 and F6). Sensitivity analyses of any alcohol‐related harms in the past year were consistent with the primary analysis (Figures G3, G4, G7 and G8; Tables G2 and G4).

### Symptoms of DSM alcohol disorders

**FIGURE 3:**
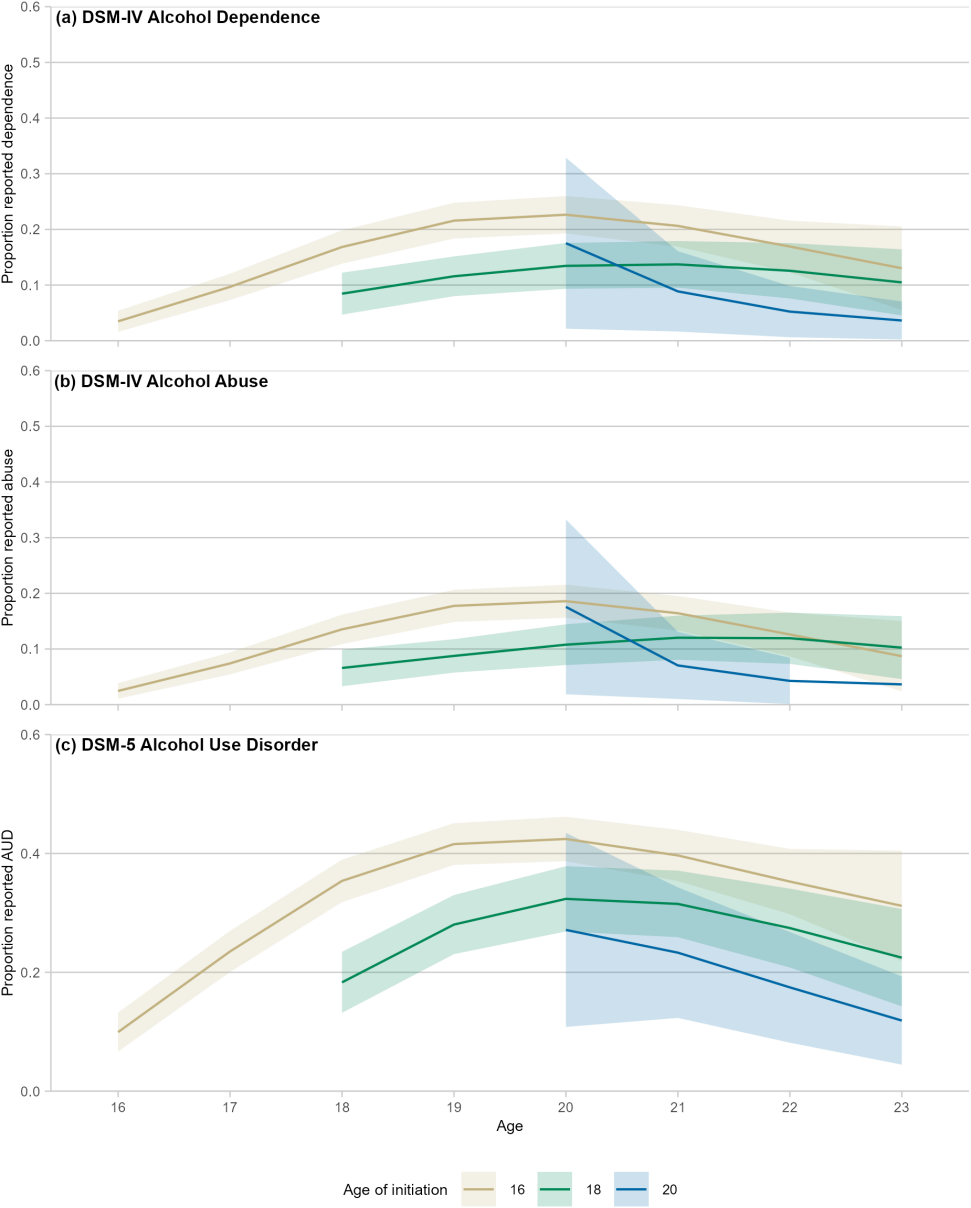
Patterns of Diagnostic and Statistical Manual of Mental Disorders (DSM)‐based outcomes, by age of initiation. Note: the Diagnostic Interview Schedule for Children (DISC) was not asked in early waves of the study, so analysis was limited to age 14+ years. Ribbons show 95% confidence intervals around the trend. Full results for the figure are included in Tables E5, E6 and E7. Models included: linear and quadratic terms for age; and linear, quadratic and cubic terms for time (age + age^2^ + time + time^2^ + time^3^). Further information on model fitting is included in Table E1.

All three DSM‐based outcomes showed similar patterns to HED and alcohol‐related harm, with higher peaks in early adulthood among those who initiated earlier (Figures 3 and E8). However, there was only evidence for more rapid increases early in DSM‐5 AUD (Figures E7, E9 and E11). While there was little difference between earlier ages of initiation by early adulthood, those who initiated at age 16 years showed higher rates of all three outcomes at age 20 years compared with those who initiated at age 20 years, as seen in Table 4. However, all three showed declines by the end of the study period (Figures 3, E8, E10 and E12).

**FIGURE 4:**
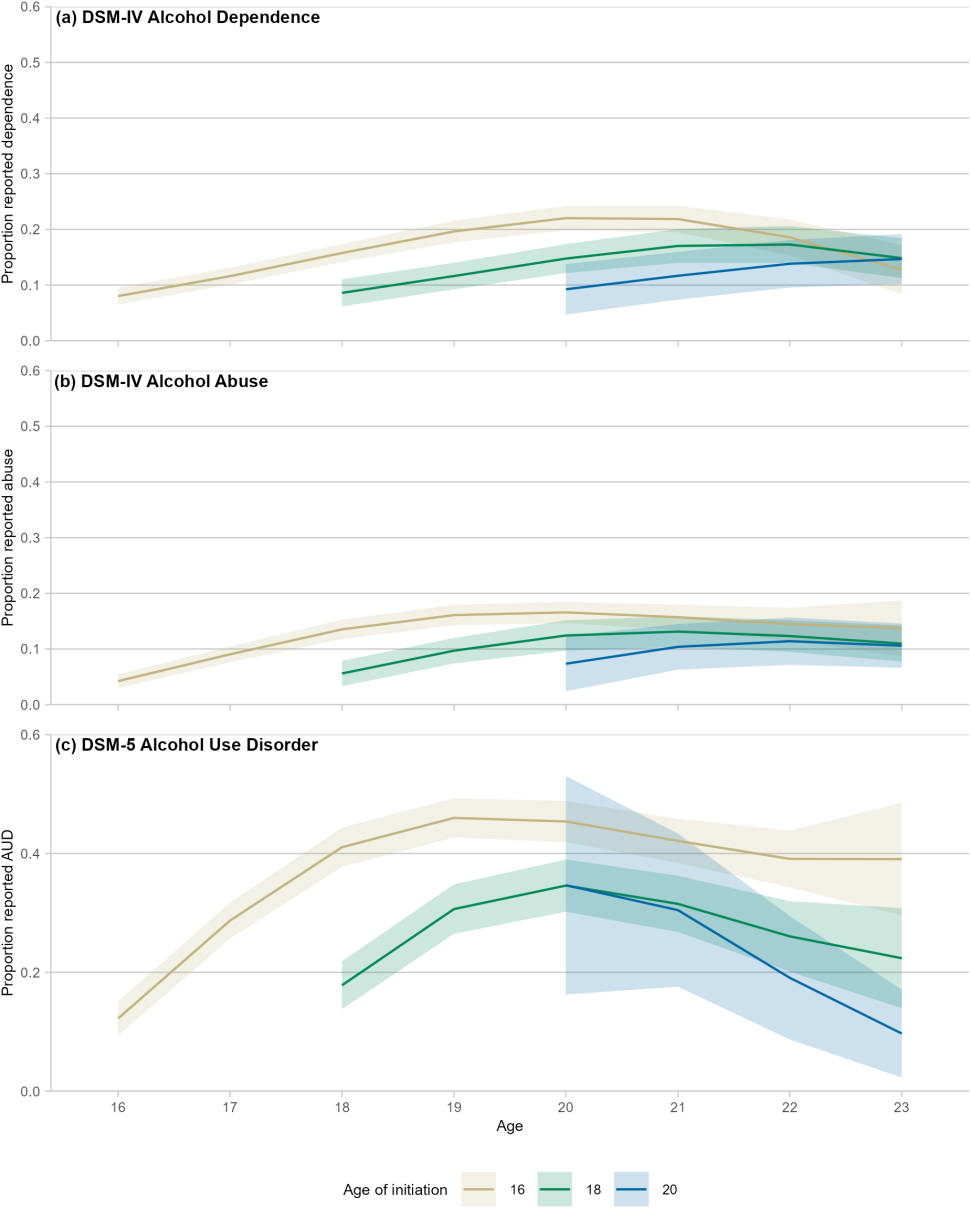
Patterns of Diagnostic and Statistical Manual of Mental Disorders (DSM)‐based outcomes, by age of initiation to whole drinks. Note: the Diagnostic Interview Schedule for Children (DISC) was not asked in early waves of the study, so analysis was limited to age 14+ years. Ribbons show 95% confidence intervals around the trend. Full results for the figure are included in Tables F7, F8, and F9. Models included: linear and quadratic terms for age; and linear, quadratic and cubic terms for time (age + age^2^ + time + time^2^ + time^3^). Further information on model fitting is included in Table F1.

In contrast, initiation to whole drinks showed quite similar early patterns in the years immediately following initiation (Figures 4, F9 and F11), regardless of the age at which initiation occurred. Those patterns continued well into adulthood, with earlier initiation linked to higher rates of dependence at age 20 years than later initiation, and similar results for abuse and AUD. However, similar to the analysis of initiation to any alcohol, dependence showed declines by the end of the study, and abuse and AUD showed either declines or plateauing (Figures 4, F8, F10 and F12).

## DISCUSSION

This prospective cohort study comprehensively examines patterns of consumption and alcohol‐related harms in the years after alcohol initiation by following participants from early adolescence (age 12 years) into early adulthood (age 23 years), controlling rigorously for a wide range of potential confounding factors.

Consistent with expectations, the results suggest that initiation at the legal age of purchase (age 18 years in Australia), or just prior to this age (approx. age 16 years), is associated with a more rapid uptake of alcohol consumption, risky drinking and alcohol‐related harm, than initiation at earlier ages. This pattern of results extended to both the level of consumption immediately following initiation (i.e. in the same year) and a more rapid increase over the following 3 years. In contrast, initiation well after reaching the legal age of purchase (e.g. age 20 years) showed a very rapid uptake of alcohol consumption immediately after initiation, followed by a decline over the next few years, rather than an increase. These findings are consistent with past research [3, 34], but extend our understanding beyond outcomes at a fixed point in time, to consider patterns of outcomes from initiation to early adulthood.

However, while the short‐term pattern of alcohol consumption, risky consumption and harm was greater with initiation at ages 16 and 18 years, the peak level of risk was lower than that with earlier ages of initiation (e.g. at ages 12 or 14 years). Specifically, while later initiation was linked to higher initial and short‐term levels of risk, the highest rates of consumption and harm in early adulthood were seen with initiation in early adolescence, consistent with past findings [3]. Importantly, this does not appear to be simply because the follow‐up period differed based on age of initiation. That is, it was not that the patterns of harm following later initiation had simply not reached their peak levels of risk by the end of the study. In fact, while average consumption largely continued to increase or remained steady, we saw evidence of ‘ageing out’ of both risky drinking and alcohol‐related harm, particularly for initiation later in adolescence or in early adulthood. This is highly relevant in light of the rapid escalation of consumption and risk among those who wait until later to start drinking alcohol, as it suggests that while risk does increase quickly following initiation in those who begin drinking at age 18 years, the risk peaks and declines without requiring intervention. Similarly, all three DSM‐based outcomes showed peaks around age 19–21 years, regardless of the age of initiation, and declined thereafter. And while earlier initiation was linked to a higher peak risk of dependence and AUD, there was also a more rapid decline into adulthood, with levels of risk very similar at age 23 years regardless of the age of alcohol initiation. This suggests that harms associated with early initiation are not permanent and can potentially be reduced in adulthood. Intervention may be necessary to reduce the peak risk among those who initiate alcohol early.

Initiation to drinking whole drinks showed largely similar trends to initiation of any alcohol consumption; however, the early patterns were steeper and were more similar across initiation ages. This suggests that an earlier initiation to whole drinks results in the same rapid escalation as initiation at or beyond the legal age of purchase. In other words, initiation to whole drink consumption earlier in adolescence resulted in similar trends, but with greater time of exposure, resulting in greater consumption and risk of harm in early adulthood. However, there was still evidence of ageing out for most of the outcomes, and by age 23 years many of the differences between earlier and later whole drink initiation had disappeared. Nonetheless, initiating whole drink consumption was linked to greater consumption, risky drinking and an elevated risk of AUD at age 23 years. Thus, while it is possible that the risk of harm may decline without intervention, reducing the peak lever of harm, and thereby any long‐term impacts from that peak, requires efforts focused on prevention and early intervention in adolescence.

Although the results suggest that the initiation of sips of alcohol in adolescence is linked to relatively short‐term risk, whereas the initiation to whole drinks is linked more to longer‐term harm, the likelihood of risk was elevated for all adolescents who consumed any amount of alcohol. Thus, the findings support the recommendation that adolescents avoid alcohol until adulthood made by national guidelines in Australia, Canada, the UK and the USA [13, 14, 15, 16]. These findings also reinforce the need for continued public health intervention aimed at both children and parents, as well as a consideration of broader established effective policy levers pertinent to the initiation and use of alcohol among young people (e.g. alcohol marketing and availability restrictions) [35, 36, 37].

It is worth considering that this study examined a single cohort of adolescents. Given the general decline in alcohol consumption by young people in Australia [38], more work is needed to examine how alcohol initiation, and any downstream implications of earlier initiation, have changed over the decade since the recruitment of this cohort. And while other countries are similar to Australia in terms of both legal age of purchase and other trends in alcohol consumption [39], further research is needed to see whether the same patterns exist in countries where legal restrictions or cultural norms are very different.

### Strengths and limitations

This study has a number of strengths. Our analysis includes regular observations of participants prior to the age of initiation for most participants (around 6% had consumed alcohol prior to entering the study). We also have a long follow‐up period, with 11 annual waves of data, at least three waves of data following alcohol initiation for all participants who initiated up to age 20 years, and six or seven waves of follow‐up data for those who initiated alcohol at the ‘average’ age in Australia (16 years) [19]. Our analysis also adjusted for a range of potential confounding factors and risk factors including child, familial and peer factors. This affords a careful and detailed study of the natural history of drinking among study participants, and thus a secure basis for the inferences drawn.

However, the study does have some limitations. First, as data was collected annually, we do not know the exact age of initiation, only that it occurred within the previous year (i.e. initiation could have occurred at any time in the previous year). This means the time between initiation and outcomes may vary between participants. Second, the sample was not a random sample, with an over‐representation of independent schools (49% of included schools) and with participants from three of the seven Australian states (New South Wales, Tasmania and Western Australia). It thus may not generalise to the Australian population. However, the sample was broadly consistent with the Australian population, with 50.5% female participants and with similar levels of alcohol use. However, the sample was more socio‐economically advantaged than the Australian population [24]. Thus, findings may not generalise to all Australian adolescents. Finally, while retention was relatively high, and we addressed missing data using multiple imputation, there remains a chance of bias arising from missing data, in particular if attrition was linked to more severe outcomes (e.g. if those with AUD were less likely to be retained in the study).

## CONCLUSION

This study suggests that an earlier initiation to alcohol, and particularly initiation to whole drinks, is associated with an elevated risk of alcohol‐related harm in early adulthood. While some of those risks showed evidence of ageing out, other risk behaviours persisted to age 23 years. Regarding the hypothesis that people who wait until the age of 18 years to initiate alcohol are ‘unleashed’, this study found that, while those initiating at 18 years or later showed more rapid increases in consumption, those who initiated earlier still reached a higher level of overall risk in early adulthood. These findings support the current recommendations to delay alcohol initiation until early adulthood.

## AUTHOR CONTRIBUTIONS

**Philip J. Clare:** Conceptualization (equal); formal analysis (lead); methodology (lead); writing—original draft (lead); writing—review and editing (lead). **Wing See Yuen:** Conceptualization (supporting); data curation (equal); project administration (equal); writing—original draft (supporting); writing—review and editing (supporting). **Alexandra Henderson:** Conceptualization (equal); data curation (equal); project administration (equal); writing—original draft (supporting); writing—review and editing (supporting). **Kypros Kypri:** Conceptualization (supporting); funding acquisition (equal); investigation (equal); writing—review and editing (supporting). **Raimondo Bruno:** Conceptualization (supporting); funding acquisition (equal); investigation (equal); writing—review and editing (supporting). **Tim Slade:** Conceptualization (supporting); funding acquisition (equal); investigation (equal); writing—review and editing (supporting). **Delyse Hutchinson:** Conceptualization (supporting); funding acquisition (equal); investigation (equal); writing—review and editing (supporting). **Nyanda McBride:** Conceptualization (supporting); funding acquisition (equal); investigation (equal); writing—review and editing (supporting). **Monika Wodalowski:** Conceptualization (supporting); data curation (equal); funding acquisition (equal); investigation (equal); project administration (equal); writing—review and editing (supporting). **Jim McCambridge:** Conceptualization (supporting); funding acquisition (equal); investigation (equal); writing—review and editing (supporting). **Louisa Degenhardt:** Conceptualization (supporting); funding acquisition (equal); investigation (equal); writing—review and editing (supporting). **Veronica C. Boland:** Conceptualization (supporting); data curation (supporting); investigation (supporting); project administration (supporting); writing—review and editing (supporting). **Richard P. Mattick:** Conceptualization (supporting); funding acquisition (equal); investigation (equal); project administration (equal); writing—review and editing (supporting). **Amy Peacock:** Conceptualization (supporting); funding acquisition (equal); investigation (equal); project administration (equal); writing—review and editing (supporting).

## DECLARATION OF INTERESTS

A.P. has received untied educational grants from Mundipharma and Seqirus for post‐marketing surveillance of pharmaceutical opioids. R.B. has received untied educational grants from Mundipharma and Indivior for studies relating to pharmaceutical opioids. Co‐authors have also received the following funding: National Health and Medical Research Council Principal Research Fellowship grants to R.M. (APP1045318); National Health and Medical Research Council Research Fellowship grants to K.K. (GNT0188568, APP1041867); and National Health and Medical Research Council Early Career Fellowship and NHMRC Investigator Fellowship grants to A.P. (APP1109366; APP1174630) and D.H. (APP1197488); Investigator Grant L3 (2016825) and NHMRC Senior Principal Research Fellowship (1135991) to L.D.; and National Health and Medical Research Council Project grants to R.P.M. for a Longitudinal Cohorts Research Consortium (GNT1009381 and GNT1064893). All other authors declare no financial conflicts of interest.

## CLINICAL TRIAL REGISTRATION

ClinicalTrials.gov (NCT02280551); secondary analysis registered at Open Science Framework (OSF; https://doi.org/10.17605/OSF.IO/BRDUV).

## Supporting information


**Table A1.** STROBE checklist.
**Table B1.** Different harms included in 14‐item harms scale.
**Table D1.** Baseline predictors of age of initiation.
**Table E1.** Assessment of model fit for non‐linear terms of age of initiation and age.
**Table E2.** Trajectories of alcohol consumption, by age of initiation.
**Table E3.** Trajectories of at least monthly heavy episodic drinking, by age of initiation.
**Table E4.** Trajectories of number of alcohol‐related harms, by age of initiation.
**Table E5.** Trajectories of DSM‐IV alcohol dependence, by age of initiation.
**Table E6.** Trajectories of DSM‐IV alcohol abuse, by age of initiation.
**Table E7.** Trajectories of DSM‐5 alcohol use disorder, by age of initiation.
**Table F1.** Assessment of model fit for non‐linear terms of age of initiation of whole drinks and age.
**Table F2.** Comparison of outcomes at age 18 years, based on age of initiation.
**Table F3.** Comparison of outcomes at age 20 years, based on age of initiation.
**Table F4.** Trajectories of alcohol consumption, by age of initiation of whole drinks.
**Table F5.** Trajectories of at least monthly heavy episodic drinking, by age of initiation of whole drinks.
**Table F6.** Trajectories of number of alcohol‐related harms, by age of initiation of whole drinks.
**Table F7.** Trajectories of DSM‐IV alcohol dependence, by age of initiation of whole drinks.
**Table F8.** Trajectories of DSM‐IV alcohol abuse, by age of initiation of whole drinks.
**Table F9.** Trajectories of DSM‐5 alcohol use disorder, by age of initiation of whole drinks.
**Table G1.** Trajectories of any heavy episodic drinking, by age of initiation.
**Table G2.** Trajectories of experience of any alcohol‐related harms, by age of initiation.
**Table G3.** Trajectories of any heavy episodic drinking, by age of initiation of whole drinks.
**Table G4.** Trajectories of experience of any alcohol‐related harms, by age of initiation of whole drinks.
**Figure A1.** Study flow chart of recruitment and assessment of APSALS cohort.
**Figure C1.** Percent of missing data in each variable.
**Figure C2.** Most common patterns of missing data.
**Figure E1.** Trajectories of alcohol consumption in the 3 years following initiation.
**Figure E2.** Trajectories of alcohol consumption for all ages of initiation.
**Figure E3.** Trajectories of at least monthly heavy episodic drinking in the 3 years following initiation.
**Figure E4.** Trajectories of at least monthly heavy episodic drinking for all ages of initiation.
**Figure E5.** Trajectories of number of alcohol‐related harms in the 3 years following initiation.
**Figure E6.** Trajectories of number of alcohol‐related harms for all ages of initiation.
**Figure E7.** Trajectories of DSM‐IV alcohol dependence in the 3 years following initiation.
**Figure E8.** Trajectories of DSM‐IV alcohol dependence for all ages of initiation.
**Figure E9.** Trajectories of DSM‐IV alcohol abuse in the 3 years following initiation.
**Figure E10.** Trajectories of DSM‐IV alcohol abuse for all ages of initiation.
**Figure E11.** Trajectories of DSM‐5 alcohol use disorder in the 3 years following initiation.
**Figure E12.** Trajectories of DSM‐5 alcohol use disorder for all ages of initiation.
**Figure F1.** Trajectories of alcohol consumption in the 3 years following initiation of whole drinks.
**Figure F2.** Trajectories of alcohol consumption for all ages of initiation of whole drinks.
**Figure F3.** Trajectories of at least monthly heavy episodic drinking in the 3 years following initiation of whole drinks.
**Figure F4.** Trajectories of at least monthly heavy episodic drinking for all ages of initiation of whole drinks.
**Figure F5.** Trajectories of number of alcohol‐related harms in the 3 years following initiation of whole drinks.
**Figure F6.** Trajectories of number of alcohol‐related harms for all ages of initiation of whole drinks.
**Figure F7.** Trajectories of DSM‐IV alcohol dependence in the 3 years following initiation of whole drinks.
**Figure F8.** Trajectories of DSM‐IV alcohol dependence for all ages of initiation of whole drinks.
**Figure F9.** Trajectories of DSM‐IV alcohol abuse in the 3 years following initiation of whole drinks.
**Figure F10.** Trajectories of DSM‐IV alcohol abuse for all ages of initiation of whole drinks.
**Figure F11.** Trajectories of DSM‐5 alcohol use disorder in the 3 years following initiation of whole drinks.
**Figure F12.** Trajectories of DSM‐5 alcohol use disorder for all ages of initiation of whole drinks.
**Figure G1.** Trajectories of any heavy episodic drinking in the 3 years following initiation.
**Figure G2.** Trajectories of any heavy episodic drinking for all ages of initiation.
**Figure G3.** Trajectories of experience of any alcohol‐related harm in the 3 years following initiation.
**Figure G4.** Trajectories of experience of any alcohol‐related harm for all ages of initiation.
**Figure G5.** Trajectories of any heavy episodic drinking in the 3 years following initiation of whole drinks.
**Figure G6.** Trajectories of any heavy episodic drinking for all ages of initiation of whole drinks.
**Figure G7.** Trajectories of experience of any alcohol‐related harm in the 3 years following initiation of whole drinks.
**Figure G8.** Trajectories of experience of any alcohol‐related harm for all ages of initiation of whole drinks.

## Data Availability

The data used in this study is not publicly available due to ethical considerations. However, researchers can request access to the data used in this study for the purpose of verification of study findings.
