# Supplementary material for: Does alcohol use and related harm differ based on the age of initiation to alcohol? Results from a prospective cohort study
Source: Addiction. 2025 Oct 13;121(1):44–57. doi: 10.1111/add.70183 (PMC12710686; doi:10.1111/add.70183)
Supplement: Supplementary file 1 — Table A1. STROBE checklist. Table B1. Different harms included in 14‐item harms scale. Table D1. Baseline predictors of age of initiation. Table E1. Assessment of model fit for non‐linear terms of age of initiation and age. Table E2. Trajectories of alcohol consumption, by age of initiation. Table E3. Trajectories of at least monthly heavy episodic drinking, by age of initiation. Table E4. Trajectories of number of alcohol‐related harms, by age of initiation. Table E5. Trajectories of DSM‐IV alcohol dependence, by age of initiation. Table E6. Trajectories of DSM‐IV alcohol abuse, by age of initiation. Table E7. Trajectories of DSM‐5 alcohol use disorder, by age of initiation. Table F1. Assessment of model fit for non‐linear terms of age of initiation of whole drinks and age. Table F2. Comparison of outcomes at age 18 years, based on age of initiation. Table F3. Comparison of outcomes at age 20 years, based on age of initiation. Table F4. Trajectories of alcohol consumption, by age of initiation of whole drinks. Table F5. Trajectories of at least monthly heavy episodic drinking, by age of initiation of whole drinks. Table F6. Trajectories of number of alcohol‐related harms, by age of initiation of whole drinks. Table F7. Trajectories of DSM‐IV alcohol dependence, by age of initiation of whole drinks. Table F8. Trajectories of DSM‐IV alcohol abuse, by age of initiation of whole drinks. Table F9. Trajectories of DSM‐5 alcohol use disorder, by age of initiation of whole drinks. Table G1. Trajectories of any heavy episodic drinking, by age of initiation. Table G2. Trajectories of experience of any alcohol‐related harms, by age of initiation. Table G3. Trajectories of any heavy episodic drinking, by age of initiation of whole drinks. Table G4. Trajectories of experience of any alcohol‐related harms, by age of initiation of whole drinks. Figure A1. Study flow chart of recruitment and assessment of APSALS cohort. Figure C1. Percent of missing data in each variable. Figure C [file ADD-121-44-s001.pdf]

Does the trajectory of alcohol use and related harm differ based on the age of initiation to alcohol? – Online Appendices

Appendix A - STROBE Reporting 1
Appendix B - Measures 4
Harms scale 4
Child variables 4
Peer variables 4
Parent variables 5
Family variables 6
References 8
Appendix C - Missing data 9
References 10
Appendix D - Post-hoc analysis of baseline predictors of age of initiation 12
Appendix E - Additional results from primary analysis 15
Appendix F - Additional results from secondary analysis 28
Appendix G - Sensitivity analysis results 43

List of tables

Table A1 STROBE checklist 1

Table B1 Different harms included in 14-items harms scale 4

Table D1 Baseline predictors of age of initiation 13

Table E1 Assessment of model fit for non-linear terms of age of initiation and age 15

Table E2 Trajectories of alcohol consumption– by age of initiation 17

Table E3 Trajectories of at least monthly heavy episodic drinking – by age of initiation 19

Table E4 Trajectories of number of alcohol related harms – by age of initiation 21

Table E5 Trajectories of DSM-IV alcohol dependence – by age of initiation 23

Table E6 Trajectories of DSM-IV alcohol abuse – by age of initiation 25

Table E7 Trajectories of DSM-5 alcohol use disorder – by age of initiation 27

Table F1 Assessment of model fit for non-linear terms of age of initiation of whole drinks and age 28

Table F2 Comparison of outcomes at age 18 based on age of initiation 29

Table F3 Comparison of outcomes at age 20 based on age of initiation 30

Table F4 Trajectories of alcohol consumption– by age of initiation of whole drinks 32

Table F5 Trajectories of at least monthly heavy episodic drinking – by age of initiation of whole drinks 34

Table F6 Trajectories of number of alcohol related harms – by age of initiation of whole drinks 36

Table F7 Trajectories of DSM-IV alcohol dependence – by age of initiation of whole drinks 38

Table F8 Trajectories of DSM-IV alcohol abuse – by age of initiation of whole drinks 40

Table F9 Trajectories of DSM-5 alcohol use disorder – by age of initiation of whole drinks 42

Table G1 Trajectories of any heavy episodic drinking – by age of initiation........ 44

Table G2 Trajectories of experience of any alcohol related harms – by age of initiation 46

Table G3 Trajectories of any heavy episodic drinking – by age of initiation of whole drinks48

Table G4 Trajectories of experience of any alcohol related harms – by age of initiation of whole drinks..50

List of figures

Figure A1 Study flowchart of recruitment and assessment of APSALS cohort........................3

Figure C1 Percent of missing data in each variable...11

Figure C2 Most common patterns of missing data 11

Figure E1 Trajectories of alcohol consumption in the three years following initiation 16

Figure E2 Trajectories of alcohol consumption for all ages of initiation 16

Figure E3 Trajectories of at least monthly heavy episodic drinking in the three years following initiation 18

Figure E4 Trajectories of at least monthly heavy episodic drinking for all ages of initiation.18

Figure E5 Trajectories of number of alcohol-related harm in the three years following initiation..20

Figure E6 Trajectories of number of alcohol-related harm for all ages of initiation...............20

Figure E7 Trajectories of DSM-IV alcohol dependence in the three years following initiation 22

Figure E8 Trajectories of DSM-IV alcohol dependence for all ages of initiation..................22

Figure E9 Trajectories of DSM-IV alcohol abuse in the three years following initiation.......24

Figure E10 Trajectories of DSM-IV alcohol abuse for all ages of initiation..........................24

Figure E11 Trajectories of DSM-5 alcohol use disorder in the three years following initiation 26

Figure E12 Trajectories of DSM-5 alcohol use disorder for all ages of initiation 26

Figure F1 Trajectories of alcohol consumption in the three years following initiation of whole drinks 31

Figure F2 Trajectories of alcohol consumption for all ages of initiation of whole drinks 31

Figure F3 Trajectories of at least monthly heavy episodic drinking in the three years following initiation of whole drinks 33

Figure F4 Trajectories of at least monthly heavy episodic drinking for all ages of initiation of whole drinks...33

Figure F5 Trajectories of number of alcohol-related harm in the three years following initiation of whole drinks 35

Figure F6 Trajectories of number of alcohol-related harm for all ages of initiation of whole drinks 35

Figure F7 Trajectories of DSM-IV alcohol dependence in the three years following initiation of whole drinks 37

Figure F8 Trajectories of DSM-IV alcohol dependence for all ages of initiation of whole drinks 37

Figure F9 Trajectories of DSM-IV alcohol abuse in the three years following initiation of whole drinks....................39

Figure F10 Trajectories of DSM-IV alcohol abuse for all ages of initiation of whole drinks.39

Figure F11 Trajectories of DSM-5 alcohol use disorder in the three years following initiation of whole drinks 41

Figure F12 Trajectories of DSM-5 alcohol use disorder for all ages of initiation of whole drinks 41

Figure G1 Trajectories of any heavy episodic drinking in the three years following initiation 43

Figure G2 Trajectories of any heavy episodic drinking for all ages of initiation....................43

Figure G3 Trajectories of experience of any alcohol-related harm in the three years following initiation....................45

Figure G4 Trajectories of experience of any alcohol-related harm for all ages of initiation...45

Figure G5 Trajectories of any heavy episodic drinking in the three years following initiation of whole drinks 47

Figure G6 Trajectories of any heavy episodic drinking for all ages of initiation of whole drinks 47

Figure G7 Trajectories of experience of any alcohol-related harm in the three years following initiation of whole drinks....................49

Figure G8 Trajectories of experience of any alcohol-related harm for all ages of initiation of whole drinks....................49

Appendix A - STROBE Reporting

Table A1 STROBE checklist

	Item No.	Recommendation	
**Title and abstract**	1	(*a*) Indicate the study's design with a commonly used term in the title or the abstract	✓
		(*b*) Provide in the abstract an informative and balanced summary of what was done and what was found	✓
**Introduction**			
Background/rationale	2	Explain the scientific background and rationale for the investigation being reported	✓
Objectives	3	State specific objectives, including any prespecified hypotheses	✓
**Methods**			
Study design	4	Present key elements of study design early in the paper	✓
Setting	5	Describe the setting, locations, and relevant dates, including periods of recruitment, exposure, follow-up, and data collection	✓
Participants	6	(*a*) Give the eligibility criteria, and the sources and methods of selection of participants. Describe methods of follow-up	✓ published elsewhere
		(*b*) For matched studies, give matching criteria and number of exposed and unexposed	N/A
Variables	7	Clearly define all outcomes, exposures, predictors, potential confounders, and effect modifiers. Give diagnostic criteria, if applicable	✓
Data sources/ measurement	8*	For each variable of interest, give sources of data and details of methods of assessment (measurement). Describe comparability of assessment methods if there is more than one group	✓
Bias	9	Describe any efforts to address potential sources of bias	✓
Study size	10	Explain how the study size was arrived at	N/A
Quantitative variables	11	Explain how quantitative variables were handled in the analyses. If applicable, describe which groupings were chosen and why	✓
Statistical methods	12	(*a*) Describe all statistical methods, including those used to control for confounding	✓
		(*b*) Describe any methods used to examine subgroups and interactions	✓
		(*c*) Explain how missing data were addressed	✓
		(*d*) If applicable, explain how loss to follow-up was addressed	✓
		(*e*) Describe any sensitivity analyses	✓
**Results**			
Participants	13*	(a) Report numbers of individuals at each stage of study—eg numbers potentially eligible, examined for eligibility, confirmed eligible, included in the study, completing follow-up, and analysed	
		(b) Give reasons for non-participation at each stage	
		(c) Consider use of a flow diagram	
Descriptive data	14*	(a) Give characteristics of study participants (eg demographic, clinical, social) and information on exposures and potential confounders	
		(b) Indicate number of participants with missing data for each variable of interest	
		(c) Summarise follow-up time (eg, average and total amount)	
Outcome data	15*	Report numbers of outcome events or summary measures over time	
Main results	16	(*a*) Give unadjusted estimates and, if applicable, confounder-adjusted estimates and their precision (eg, 95% confidence interval). Make clear which confounders were adjusted for and why they were included	

		(*b*) Report category boundaries when continuous variables were categorized	✓
		(*c*) If relevant, consider translating estimates of relative risk into absolute risk for a meaningful time period	N/A
Other analyses	17	Report other analyses done—eg analyses of subgroups and interactions, and sensitivity analyses	
**Discussion**			
Key results	18	Summarise key results with reference to study objectives	
Limitations	19	Discuss limitations of the study, taking into account sources of potential bias or imprecision. Discuss both direction and magnitude of any potential bias	
Interpretation	20	Give a cautious overall interpretation of results considering objectives, limitations, multiplicity of analyses, results from similar studies, and other relevant evidence	
Generalisability	21	Discuss the generalisability (external validity) of the study results	
**Other information**			
Funding	22	Give the source of funding and the role of the funders for the present study and, if applicable, for the original study on which the present article is based	✓

Appendix A - STROBE reporting

Figure A1 Study flowchart of recruitment and assessment of APSALS cohort

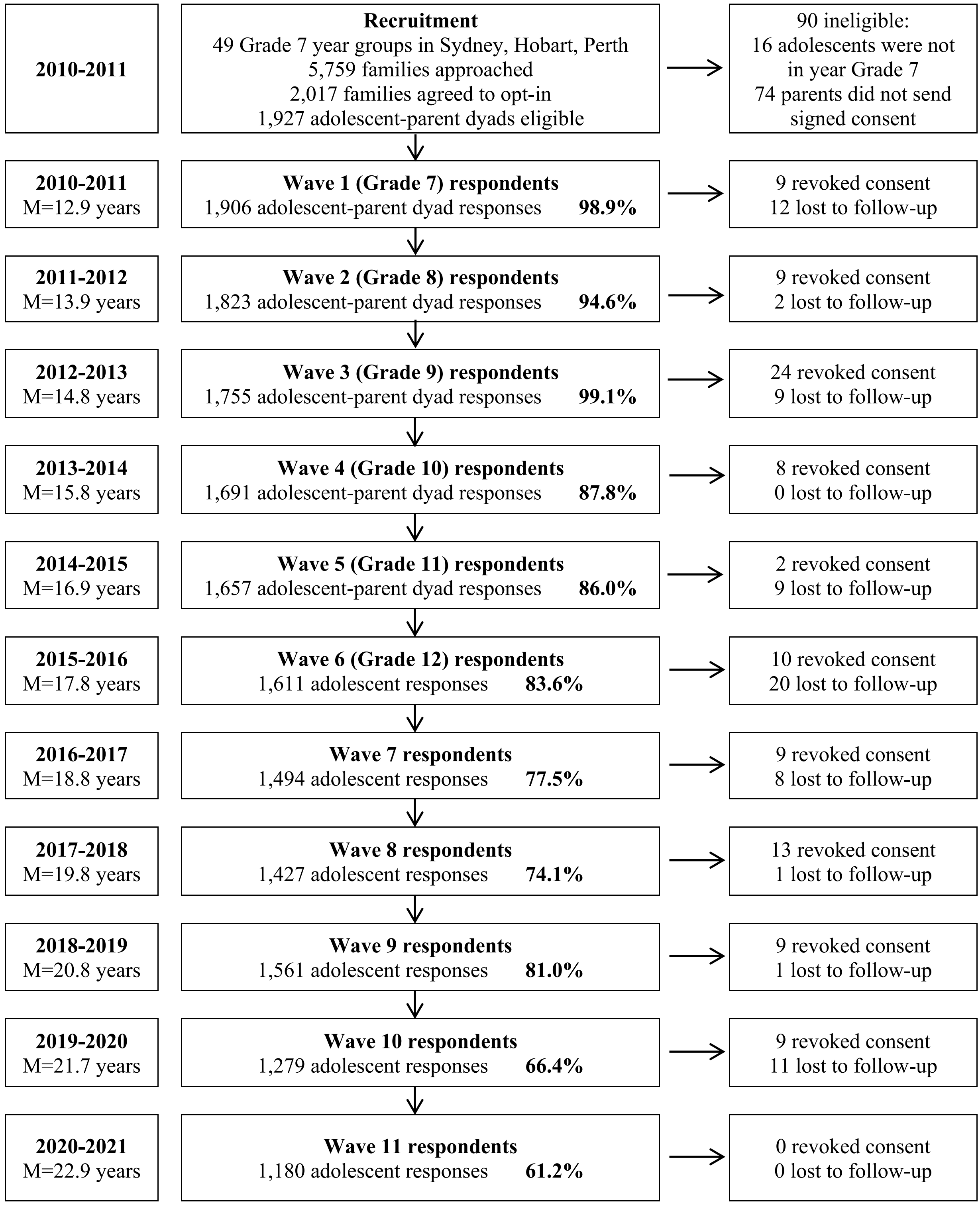

Appendix B - Measures

Harms scale

The 14 items included in the harms scale are included in table B1.

Table B1 Different harms included in 14-items harms scale

**Harm**
Did you drink more than you planned?
Were you sick after drinking?
Did you have a hangover after drinking?
Have you been unable to remember what had happened while you had been drinking?
Did you get in a physical fight with someone because you were affected by alcohol?
Did you damage something because you were affected by alcohol?
When affected by alcohol, did you have sex that you later regretted?
When affected by alcohol, did you have sex you were afraid would lead to pregnancy or sexually transmitted disease?
When affected by alcohol, have you been sexually harassed?
Has your school/uni/TAFE/work etc. performance been affected by your use of alcohol?
Did you get into trouble with your friends because of alcohol (your friends got annoyed with you)?
Did someone you were going out with complain about your drinking?
Did you get into trouble with your parents because of your drinking?
Did you get into trouble at school/uni/TAFE/work etc. because of your drinking?

Child variables

Adolescents were asked their sex, and also whether they had money to purchase alcohol, should they wish to.

Child behavior checklist (CBCL)

The study including 5 subscales from the Child behavior checklist (CBCL) (1): Rule-Breaking Behaviour, Aggressive Behaviour, Withdrawn-Depressed, Anxious-Depressed, and Social Problems. The first two were combined into the composite scale of externalizing problems. The other three subscales were included separately.

Peer variables

Peer use of alcohol and/or tobacco

Appendix B – Measures

We included six items adapted from the 2011 Monitoring the Future (MTF) survey (2), asking adolescents about their peers' quantity and frequency of alcohol consumption, and smoking tobacco, with responses ranging from none to all (of their peers). Items were summed (score range: 6-30), with higher scores indicated higher levels of peer alcohol and/or tobacco use.

Peer disapproval of alcohol and/or tobacco use

We also included four items, also adapted from the 2011 MTF (2), to measure perceptions of peer disapproval of substance use. Adolescents were asked how they thought their close friends would feel about them: smoking cigarettes, consuming any alcohol, consuming 1-2 drinks daily, and binge drinking over weekends. Each item had three response options: not disapprove, disapprove, and strongly disapprove. These items were summed (score range: 4-12), with a higher score indicating more peer disapproval of substance use.

Parent variables

Parent were asked if they were born in Australia, what their highest level of education was, what their employment status was, and how religious they were.

Parental alcohol consumption

Parents were asked how often they consumed alcohol (3), and how many standard drinks they typically consumed in the past 12-months, based on a measure from the National Drug Strategy Household Survey (4). Both were given a numerical code based on the response, or the mid-point in the case of a range. These numbers were then multiplied together to generate an estimate of overall alcohol consumption.

Parental monitoring

We assessed parental monitoring using a validated six-item scale with good internal consistency (Cronbach's alpha=0.87)(5, 6). The scale includes items such as: "My parent(s) usually know what I am doing after school"; and "I talk to my parent(s) about the plans I have with friends". Each item was coded from never (1) to always (5), and responses were summed, with a higher score indicating stricter monitoring (score range: 6-30).

Parenting consistency

We assessed parenting consistency using two five-item subscales derived from a previous study(7), assessing rule enforcement and discipline. Rule enforcement includes items such as,

“I soon forgot the rules I had made”, and “I changed my mind to make things easier for myself”, while the discipline subscale was comprised of items such as, “I usually don’t find out about my child’s misbehaviour”, and “I seldom insisted that my child do anything”. All items were scored on a five-point Likert scale, with response options ranging between strongly agree and strongly disagree. Each subscale was scored individually then averaged, providing an overall score of parenting consistency (score range 5-25).

Parental responsiveness/demandingness

We used the Authoritative Parenting Index (8) to measure parental responsiveness (9 items) and demandingness (7 items). For each item, adolescents were asked to think about their mother or step-mother, or a maternal figure, in relation to each item, and select one of four response options, ranging from “just like my mum” to “not like my mum”. Higher scores on the 9-item responsiveness subscale (score range: 9-36) indicated increased responsiveness. Likewise, higher scores on the 7-item demandingness subscale (score range: 7-24) indicate increased demandingness.

Family variables

Parents were asked the households income, and adolescents were asked about their family structure (whether they lived in a single parent household, or had older siblings).

Alcohol-specific rules

We assessed alcohol-specific rules using a 10-item scale developed in the Netherlands (9). The 10 items included a range of situations in which drinking might occur, such as “I am allowed to drink alcohol at home when my father or mother is around” and “I am allowed to drink alcohol with my friends at a party”. Responses to each item were on a 5-item scale, and were summed, with a higher score indicating stricter alcohol-specific rules (score range: 10-50).

Home access to alcohol

We measured access to alcohol in the family home with a six-item validated scale (10). Items included: “do you keep track of the alcohol supply in your home?” and “how likely do you think it is that your child would have taken alcohol from your home without you knowing?” Higher scores indicated increased levels of access to alcohol in the family home (score range: 6-20).

Appendix B – Measures

Family conflict/positive relations

We assessed family conflict and positive relations using two three-item scales, based on a previous study (11). Responses options for each item were dichotomous (yes or no), with scores for each scale ranging from 3 to 6.

Area level socioeconomic status

As an estimate of the relative socioeconomic status of the area in which the child lived, we included the Socio-Economic Indexes for Areas (SEIFA) Index of Relative Socio-Economic Advantage/Disadvantage (IRSAD) created by the Australian Bureau of Statistics (12). This was calculated based on the postcode of the area of residence, provided at wave 1. The IRSAD score is calculated using Australian national census data and a wide range of socioeconomic variables are used in the construction of the index including household income, educational levels, occupational levels, rent and mortgage repayments, dwelling size and number of occupants and disability rates within the area (13).

Appendix B – Measures

References

1. Achenbach TM. Manual for the youth self-report and 1991 profile: University of Vermont Department of Psychiatry; 1991.

2. Johnston LD, O'Malley PM, Bachman JG, Schulenberg JE. Monitoring the Future national survey results on drug use, 1975-2012. Volume II: College students and adults ages 19-50: Ann Arbor, MI: Institute for Social Research, University of Michigan; 2013.

3. Donovan JE, Molina BS. Childhood risk factors for early-onset drinking. Journal of studies on alcohol and drugs. 2011;72(5):741-51.

4. Australian Institute of Health and Welfare. National Drug Strategy Household Survey 2019. Canberra: AIHW; 2020. Contract No.: PHE 270.

5. Silverberg S, Small S, editors. Parental monitoring, family structure and adolescent substance use. meeting of the Society of Research in Child Development, Seattle, WA; 1991.

6. Small SA, Luster T. Adolescent sexual activity: An ecological, risk-factor approach. Journal of Marriage and the Family. 1994:181-92.

7. Stice E, Barrera Jr M, Chassin L. Prospective differential prediction of adolescent alcohol use and problem use: examining the mechanisms of effect. Journal of abnormal psychology. 1998;107(4):616.

8. Jackson C, Henriksen L, Foshee VA. The Authoritative Parenting Index: predicting health risk behaviors among children and adolescents. Health Education & Behavior. 1998;25(3):319-37.

9. Van Der Vorst H, Engels RC, Meeus W, Deković M, Van Leeuwe J. The role of alcohol-specific socialization in adolescents' drinking behaviour. Addiction. 2005;100(10):1464-76.

10. Komro KA, Maldonado-Molina MM, Tobler AL, Bonds JR, Muller KE. Effects of home access and availability of alcohol on young adolescents' alcohol use. Addiction. 2007;102(10):1597-608.

11. Ary DV, Duncan TE, Duncan SC, Hops H. Adolescent problem behavior: The influence of parents and peers. Behaviour research and therapy. 1999;37(3):217-30.

12. Statistics ABO. Socio-economic indexes for areas (SEIFA). Canberra: Australian Bureau of Statistics. 2011.

13. Pink B. An introduction to socio-economic indexes for areas (SEIFA). Canberra: Australian Bureau of Statistics. 2006:24-32.

Appendix C - Missing data

As a prospective cohort with 11 waves of data, there are a number of different mechanisms for missing data in the APSALS dataset:

1. Non-completion of waves, included loss to follow-up;
2. Questions not asked in some waves; and
3. Intermittent missingness due to refusal to answer, not knowing, etc.

The proportion of missing data in each variable is shown in Figure C1, and patterns of missing data are shown in Figure C2. We first confirmed that data was not missing completely at random using Little's test (1). As such, we have assumed the any missing data is missing at random. Because ignoring missingness related to both the outcome and exposure variables can introduce bias (2), we conducted all analyses primary using multiple imputation.

We imputed the data using fully conditional expectation (otherwise known as chained equations), using the R package 'mice' (3). To ensure compatibility between the imputation and outcomes models without explicitly specifying complexity such as non-linear association, we imputed all variables using random forests (4), from the package 'ranger' (5). Based on the proportion of missing information in the data, we used M=20 imputations (6). We then conducted analyses on each imputed dataset, and combined the results using Rubin's rules with the R package 'Amelia' (7).

References

1. Little RJ, Rubin DB. Statistical analysis with missing data: John Wiley & Sons; 2019.
2. Hughes RA, Heron J, Sterne JAC, Tilling K. Accounting for missing data in statistical analyses: multiple imputation is not always the answer. International Journal of Epidemiology. 2019;48(4):1294-304.
3. van Buuren S, Groothuis-Oudshoorn K. mice: multivariate imputation by chained equations in R. Journal of Statistical Software. 2011;45(3).
4. Shah AD, Bartlett JW, Carpenter J, Nicholas O, Hemingway H. Comparison of random forest and parametric imputation models for imputing missing data using MICE: a CALIBER study. American journal of epidemiology. 2014;179(6):764-74.
5. Wright MN, Wager S, Probst P. Ranger: A fast implementation of random forests. R package version 012. 2020;1.
6. Graham JW, Olchowski AE, Gilreath TD. How many imputations are really needed? Some practical clarifications of multiple imputation theory. Prevention Science. 2007;8(3):206-13.
7. Honaker J, King G, Blackwell M. Amelia II: a program for missing data. Journal of Statistical Software. 2011;45(7):47.

Appendix C – Missing data

Figure C1 Percent of missing data in each variable

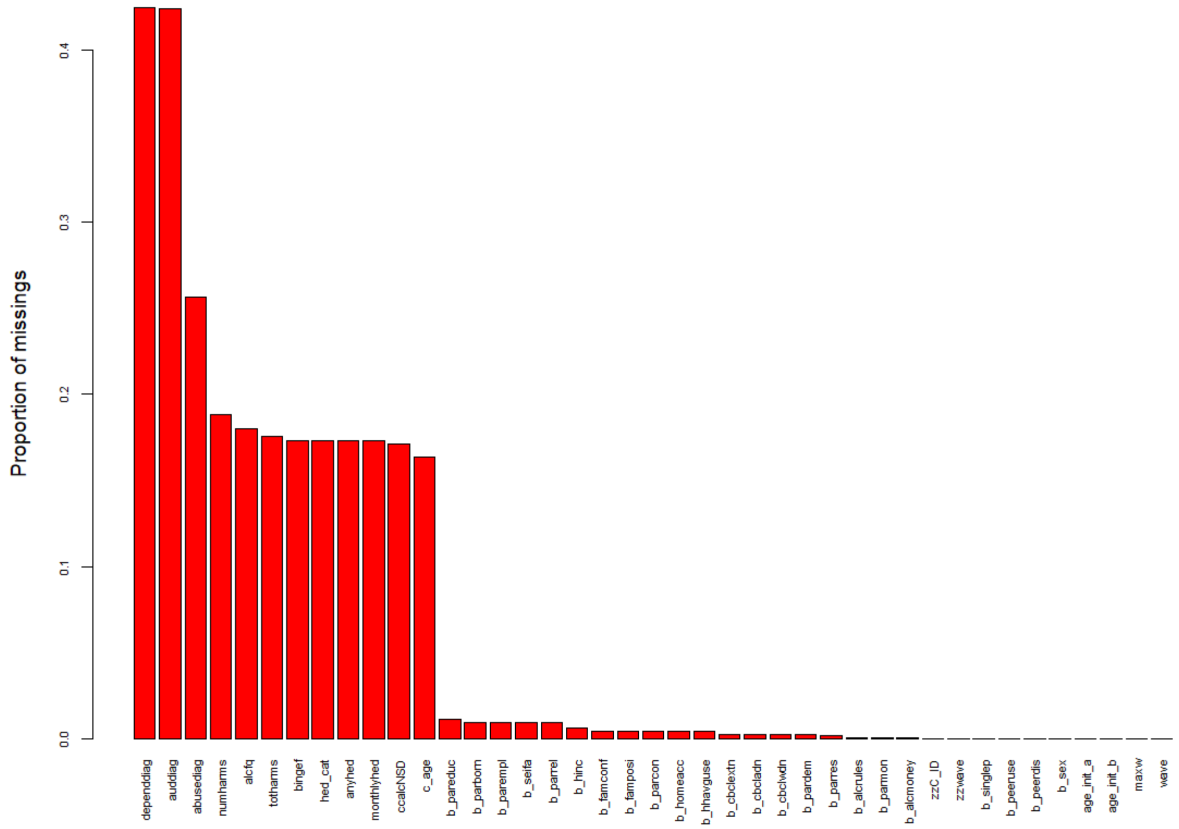

Figure C2 Most common patterns of missing data

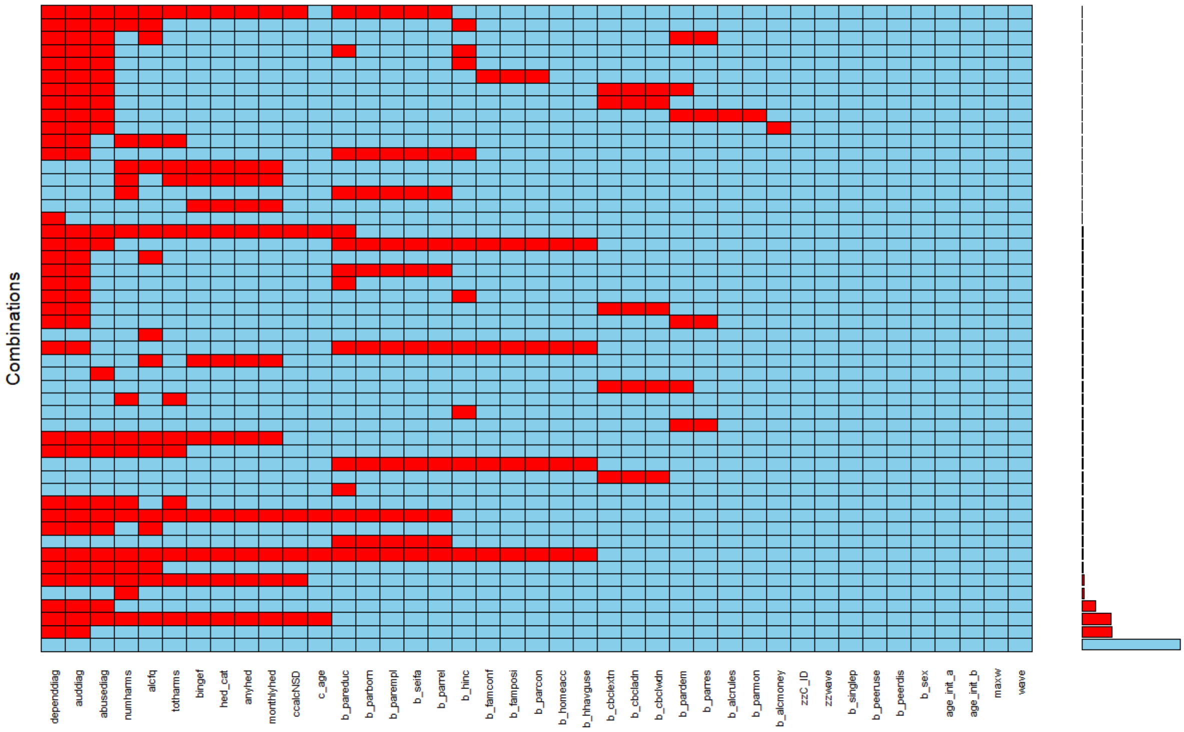

Appendix D - Post-hoc analysis of baseline predictors of age of initiation

For each standard deviation increase in the alcohol-specific rules scale (toward more rule setting), age of initiation of sips increased by 0.28 years (95% CI: 0.10, 0.46), and age of initiation of whole drinks increased by 0.27 years (99.5% CI: 0.10, 0.44). For each standard deviation increase in the peer perception of substance use scale (toward greater acceptance of alcohol by peers), age of initiation of sips decreased by 0.83 years (99.5% CI: -1.02, -0.64), and age of initiation of whole drinks decreased by 0.78 years (99.5% CI: -0.96, -0.59).

Appendix D – Post-hoc analysis of baseline predictors

Table D1 Baseline predictors of age of initiation

**Variable**		**Age of initiation of sips in years**		**Age of initiation of whole drinks in years**	
		**Coef (95% CI)**	**p-value**	**Coef (95% CI)**	**p-value**
**Adolescent**					
Sex	Male	*REF*		*REF*	
	Female	-0.47 (-0.79, -0.14)	p=0.006	-0.42 (-0.74, -0.11)	p=0.008
Has money to buy alcohol	No	*REF*		*REF*	
	Yes	0.13 (-0.21, 0.47)	p=0.456	0.13 (-0.19, 0.45)	p=0.437
CBCL - Externalising		-0.20 (-0.42, 0.02)	p=0.073	-0.28 (-0.49, -0.06)	p=0.010
CBCL - Anxious		-0.05 (-0.31, 0.21)	p=0.693	0.00 (-0.25, 0.24)	p=0.980
CBCL - Anxious		0.27 (0.02, 0.52)	p=0.038	0.25 (0.01, 0.49)	p=0.043
**Parent**					
Parent born in Australia	No	*REF*		*REF*	
	Yes	-0.14 (-0.52, 0.25)	p=0.485	-0.06 (-0.42, 0.30)	p=0.753
Parent education	High school or less	*REF*		*REF*	
	Diploma, Trade, non-trade	-0.30 (-0.72, 0.12)	p=0.155	-0.28 (-0.68, 0.12)	p=0.166
	University degree	-0.17 (-0.60, 0.27)	p=0.454	-0.15 (-0.57, 0.26)	p=0.463
Parent employment	Employed (full-time/part-time)	*REF*		*REF*	
	Unemployed - in workforce	0.32 (-0.19, 0.83)	p=0.220	0.40 (-0.09, 0.89)	p=0.106
	Unemployed - not in workforce	0.06 (-0.53, 0.64)	p=0.843	0.02 (-0.54, 0.58)	p=0.947
Parent demandingness		0.00 (-0.04, 0.05)	p=0.902	0.00 (-0.04, 0.05)	p=0.869
Parent responsiveness		-0.02 (-0.06, 0.03)	p=0.506	-0.01 (-0.06, 0.03)	p=0.511
Parent religiousity	Not religious/a little religious	*REF*		*REF*	
	Pretty religious/very religious	0.20 (-0.16, 0.56)	p=0.280	0.14 (-0.21, 0.48)	p=0.430
**Household**					
SEIFA tertile	Low	*REF*		*REF*	

Appendix D – Post-hoc analysis of baseline predictors

	Medium	-0.08 (-0.63, 0.48)	p=0.783	0.11 (-0.42, 0.63)	p=0.687
	High	0.05 (-0.45, 0.55)	p=0.850	0.19 (-0.28, 0.67)	p=0.427
Household income	Up to $34,000	*REF*		*REF*	
	$35-80,000	-0.28 (-0.96, 0.40)	p=0.421	-0.40 (-1.05, 0.25)	p=0.231
	$81-180,000	-0.23 (-0.94, 0.48)	p=0.523	-0.23 (-0.91, 0.44)	p=0.498
	$180,001+	-0.57 (-1.34, 0.21)	p=0.153	-0.53 (-1.27, 0.21)	p=0.161
Single parent household	No	*REF*		*REF*	
	Yes	-0.28 (-0.74, 0.19)	p=0.246	-0.29 (-0.74, 0.16)	p=0.203
Parent alcohol use		-0.02 (-0.19, 0.15)	p=0.803	-0.05 (-0.21, 0.11)	p=0.523
Alcohol-specific rules		0.28 (0.10, 0.46)	p=0.002	0.27 (0.10, 0.44)	p=0.002
Parental monitoring		0.22 (0.02, 0.42)	p=0.030	0.21 (0.02, 0.40)	p=0.030
Parenting consistency		0.08 (-0.09, 0.25)	p=0.359	0.07 (-0.10, 0.23)	p=0.420
Home access to alcohol	No	*REF*		*REF*	
	Yes	-0.09 (-0.27, 0.10)	p=0.351	-0.07 (-0.25, 0.11)	p=0.435
Family conflict		-0.12 (-0.30, 0.07)	p=0.211	-0.13 (-0.30, 0.04)	p=0.144
Family positive relations		0.22 (0.04, 0.40)	p=0.014	0.19 (0.03, 0.36)	p=0.024
**Peer perceptions**					
Peer substance use		-0.83 (-1.02, -0.64)	p<0.001	-0.78 (-0.96, -0.59)	p<0.001
Peer disapproval of substance use		0.02 (-0.16, 0.21)	p=0.812	-0.01 (-0.19, 0.17)	p=0.919

Appendix E - Additional results from primary analysis

Table E1 Assessment of model fit for non-linear terms of age of initiation and age

Age of initiation	Time	Number of drinks		Monthly HED		Any HED		Number of harms		Any harms		DSM-IV Dependence		DSM-IV Abuse		DSM-5 AUD	
		**AIC**	**BIC**	**AIC**	**BIC**	**AIC**	**BIC**	**AIC**	**BIC**	**AIC**	**BIC**	**AIC**	**BIC**	**AIC**	**BIC**	**AIC**	**BIC**
x	z	79289	79490	7824	8018	6859	7054	28351	28552	7510	7705	4847	5035	4014	4202	6849	7037
x + x^2	z	79195	79410	7801	8010	6825	7033	28275	28490	7448	7656	4847	5048	4011	4212	6844	7045
x + x^3	z	79198	79413	7797	8006	6816	7025	28281	28496	7445	7654	4848	5049	4013	4214	6844	7045
x + x^2 + x^3	z	79195	79424	7797	8019	6808	7030	28271	28500	7442	7664	4841	5056	4009	4223	6832	7047
x	z + z^2	78395	78610	7429	7637	6467	6675	27610	27826	7216	7425	4783	4984	3962	4163	6739	6940
x + x^2	z + z^2	78259	**78495**	7339	7568	6375	6604	27467	27703	7108	7338	4749	**4970**	3931	4152	6669	6890
x + x^3	z + z^2	78261	78497	7336	7566	6366	6596	27480	27717	7105	7335	4750	4971	3930	**4151**	6668	**6889**
x + x^2 + x^3	z + z^2	78253	78510	7324	7574	6351	6602	27437	**27694**	7093	7343	4746	4988	3923	4164	6667	6909
x	z + z^3	78429	78644	7454	7662	6459	6667	27657	27872	7207	7415	4789	4990	3969	4170	6752	6953
x + x^2	z + z^3	78291	78527	7362	7591	6366	6595	27510	27746	7094	7324	4760	4981	3939	4160	6692	6913
x + x^3	z + z^3	78294	78530	7360	7590	6358	6588	27523	27759	7092	7321	4761	4982	3940	4161	6689	6910
x + x^2 + x^3	z + z^3	78288	78545	7347	7597	6342	6592	27484	27741	7078	7328	4756	4997	3929	4170	6686	6927
x	z + z^2 + z^3	78390	78619	7418	7640	6442	6665	27600	27829	7177	7400	4785	5000	3964	4178	6738	6952
x + x^2	z + z^2 + z^3	78240	78497	7301	7551	6343	6593	27447	27704	7063	7313	**4739**	4980	**3919**	4160	**6652**	6893
x + x^3	z + z^2 + z^3	**78239**	78496	**7299**	**7549**	6331	**6581**	27457	27714	**7057**	**7307**	4742	4983	3921	4162	6656	6897
x + x^2 + x^3	z + z^2 + z^3	78239	78524	7303	7581	**6331**	6608	**27436**	27721	7062	7340	4742	5010	3924	4192	6656	6924

Note: best performing model based on each criteria is highlighted in bold. Final model was chosen based on AIC to prioritise predictions, because results are based on marginal predicted means based on the models, not the model parameters themselves.

Appendix G - Sensitivity analysis results

Figure E1 Trajectories of alcohol consumption in the three years following initiation

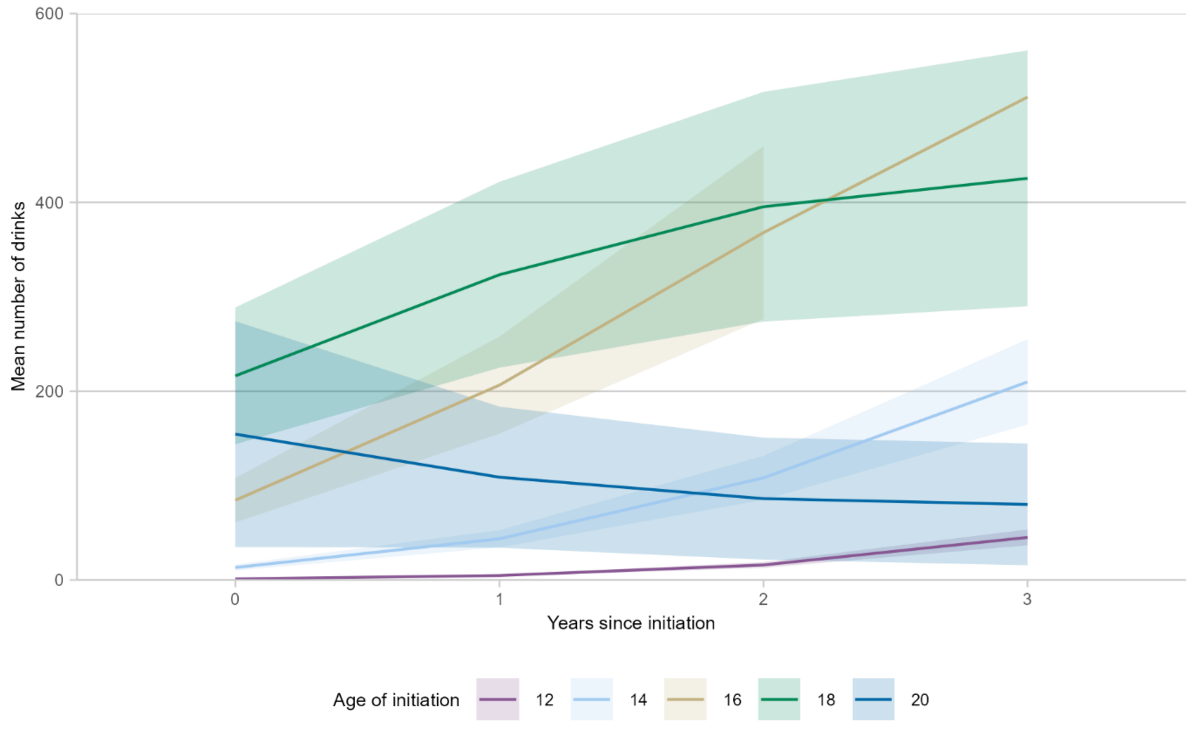

Figure E2 Trajectories of alcohol consumption for all ages of initiation

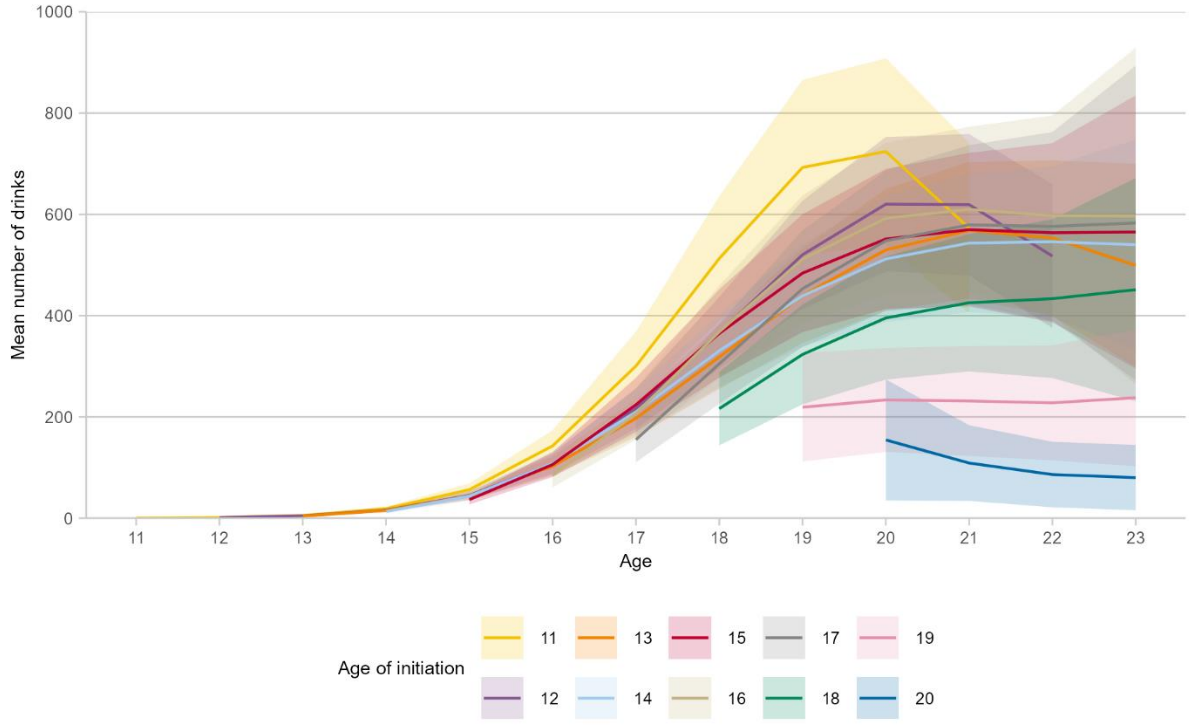

Model included linear and cubic terms for age; and linear, quadratic, and cubic terms for time (age + age^3 + time + $time^2$ + $time^3$). Further information on model fit is included in Appendix Table E1.

Table E2 Trajectories of alcohol consumption– by age of initiation

		Mean number of drinks (95% CI)												
		**Age**												
		**11**	**12**	**13**	**14**	**15**	**16**	**17**	**18**	**19**	**20**	**21**	**22**	**23**
**Age of initiation**	11	0.3 (0.2, 0.4)	1.4 (1.0, 1.8)	5.5 (4.2, 6.7)	18.8 (14.5, 23.1)	56.3 (44.0, 68.5)	142.8 (112.1, 173.5)	300.6 (233.2, 367.9)	512.7 (389.9, 635.6)	692.7 (519.8, 865.5)	724.1 (540.0, 908.1)	572.7 (404.7, 740.6)		
	12		1.2 (0.9, 1.5)	4.8 (3.8, 5.7)	15.9 (13.0, 18.9)	45.1 (36.8, 53.4)	107.7 (88.1, 127.2)	216.5 (176.9, 256.1)	366.2 (295.7, 436.7)	520.2 (413.7, 626.8)	620.1 (487.6, 752.6)	619.2 (479.9, 758.6)	517.6 (375.7, 659.4)	
	13			4.2 (3.2, 5.1)	15.2 (12.4, 18.0)	44.0 (35.8, 52.2)	102.7 (83.5, 121.8)	197.3 (160.1, 234.4)	318.2 (255.5, 380.9)	439.5 (346.3, 532.8)	529.8 (410.5, 649.2)	568.4 (433.6, 703.2)	553.4 (400.0, 706.8)	499.2 (298.7, 699.6)
	14				13.2 (10.2, 16.3)	43.6 (34.6, 52.6)	108.2 (85.1, 131.3)	209.8 (164.8, 254.9)	330.4 (258.8, 402.1)	439.2 (338.9, 539.5)	511.9 (387.7, 636.1)	543.4 (407.1, 679.7)	545.9 (396.7, 695.1)	540.0 (332.7, 747.3)
	15					36.7 (27.2, 46.1)	106.0 (81.3, 130.7)	224.0 (170.4, 277.5)	364.9 (278.0, 451.8)	483.9 (367.6, 600.2)	551.4 (413.1, 689.6)	569.7 (418.1, 721.3)	563.7 (386.8, 740.5)	565.0 (295.2, 834.9)
	16						84.5 (61.2, 107.9)	206.4 (155.2, 257.6)	368.0 (276.6, 459.3)	511.5 (385.9, 637.1)	592.2 (443.5, 740.9)	609.9 (447.1, 772.7)	597.3 (399.5, 795.2)	596.7 (264.7, 928.7)
	17							155.1 (110.7, 199.5)	304.8 (226.0, 383.7)	453.2 (338.2, 568.1)	547.8 (409.1, 686.5)	579.2 (421.9, 736.5)	576.3 (390.0, 762.5)	582.9 (272.5, 893.4)
	18								216.3 (143.9, 288.6)	323.3 (225.0, 421.7)	395.4 (273.8, 517.0)	425.4 (290.0, 560.9)	433.5 (276.8, 590.1)	451.2 (230.9, 671.5)
	19									219.2 (112.3, 326.1)	233.4 (130.9, 336.0)	231.4 (123.1, 339.7)	227.7 (114.4, 341.0)	237.9 (102.4, 373.4)
	20										154.5 (35.0, 274.0)	108.9 (34.2, 183.6)	86.2 (21.6, 150.8)	80.1 (15.7, 144.5)

Figure E3 Trajectories of at least monthly heavy episodic drinking in the three years following initiation

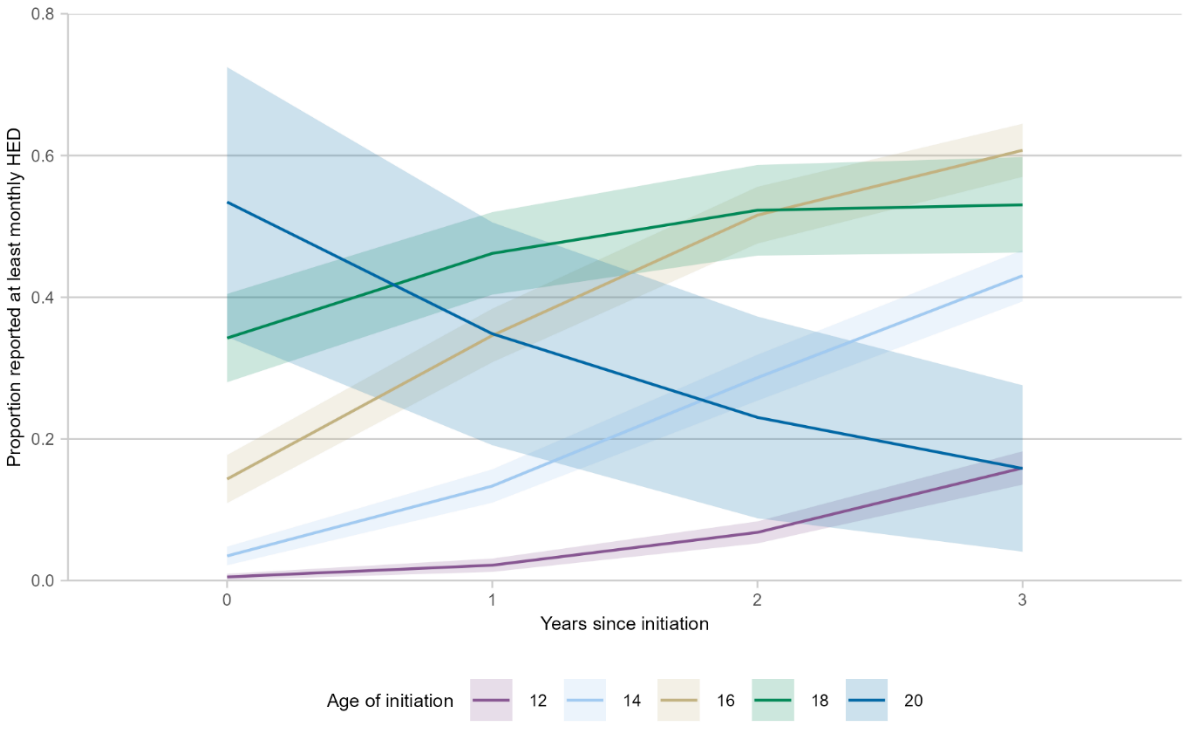

Figure E4 Trajectories of at least monthly heavy episodic drinking for all ages of initiation

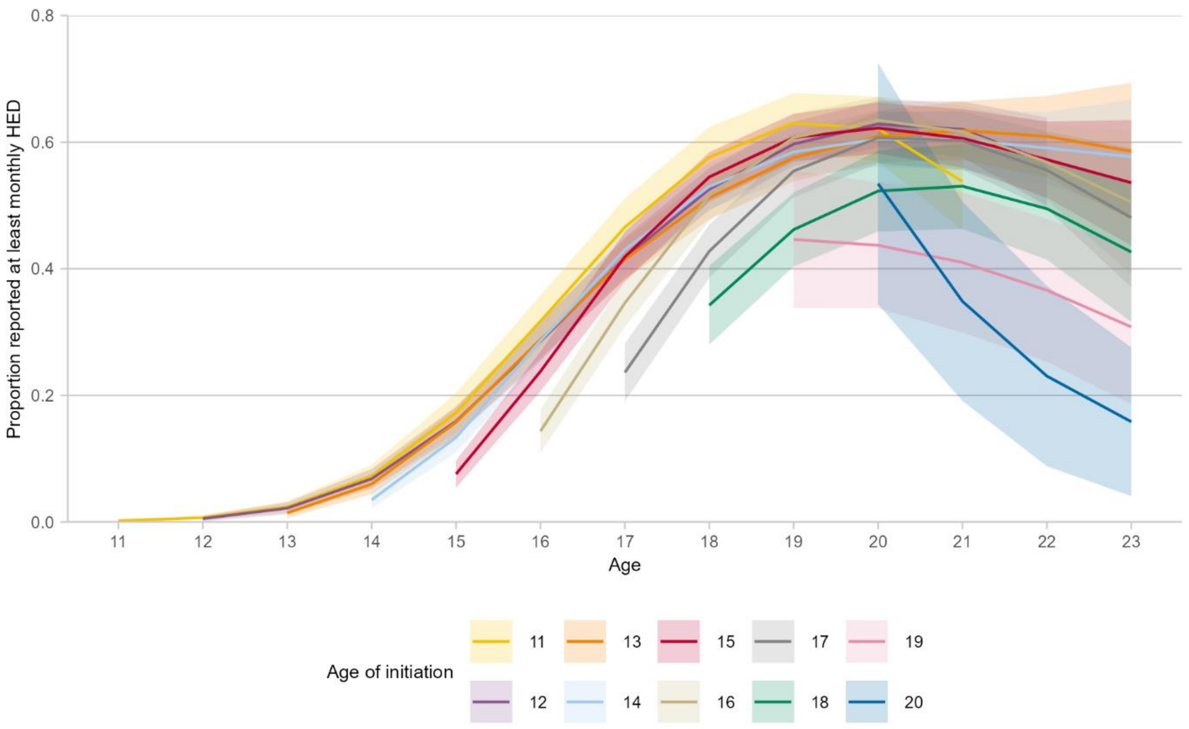

Model included linear and cubic terms for age; and linear, quadratic, and cubic terms for time (age + age^3 + time + $time^2$ + $time^3$). Further information on model fit is included in Appendix Table E1.

Table E3 Trajectories of at least monthly heavy episodic drinking – by age of initiation

		**Proportion reported monthly heavy episodic drinking (95% CI)**												
		**Age**												
		**11**	**12**	**13**	**14**	**15**	**16**	**17**	**18**	**19**	**20**	**21**	**22**	**23**
**Age of initiation**	11	0.2 (0.0, 0.4)	0.7 (0.2, 1.1)	2.4 (1.5, 3.3)	7.2 (5.5, 9.0)	17.3 (14.2, 20.3)	31.7 (27.7, 35.8)	46.6 (42.0, 51.1)	57.6 (52.9, 62.4)	63.0 (58.3, 67.8)	62.1 (57.0, 67.2)	53.8 (46.1, 61.4)		
	12		0.5 (0.1, 1.0)	2.2 (1.2, 3.1)	6.8 (5.3, 8.4)	15.9 (13.6, 18.2)	28.5 (25.5, 31.6)	41.7 (38.4, 45.0)	52.6 (49.2, 55.9)	59.7 (56.2, 63.2)	62.9 (59.2, 66.5)	62.0 (57.6, 66.4)	56.9 (49.9, 63.9)	
	13			1.4 (0.6, 2.2)	6.0 (4.4, 7.6)	15.8 (13.4, 18.1)	28.8 (26.0, 31.7)	41.5 (38.3, 44.7)	51.2 (47.8, 54.6)	57.6 (53.8, 61.3)	60.9 (56.8, 65.0)	61.8 (57.2, 66.5)	60.9 (54.5, 67.3)	58.6 (47.7, 69.4)
	14				3.5 (2.2, 4.8)	13.4 (11.0, 15.7)	28.6 (25.4, 31.9)	43.0 (39.4, 46.6)	53.0 (49.4, 56.6)	58.5 (54.5, 62.4)	60.5 (56.2, 64.9)	60.4 (55.6, 65.1)	59.1 (53.4, 64.7)	57.7 (48.7, 66.7)
	15					7.6 (5.4, 9.7)	23.8 (20.6, 26.9)	41.9 (38.0, 45.8)	54.5 (50.6, 58.4)	60.7 (57.0, 64.5)	62.2 (58.2, 66.3)	60.6 (56.0, 65.2)	57.2 (51.2, 63.2)	53.6 (43.7, 63.5)
	16						14.3 (11.0, 17.7)	34.6 (30.8, 38.4)	51.6 (47.6, 55.6)	60.7 (57.0, 64.5)	63.5 (59.8, 67.1)	61.7 (57.5, 65.8)	56.8 (50.9, 62.8)	50.5 (39.2, 61.8)
	17							23.6 (19.1, 28.2)	42.8 (38.6, 46.9)	55.4 (51.3, 59.6)	60.7 (56.6, 64.7)	60.2 (55.6, 64.8)	55.6 (49.5, 61.7)	48.1 (37.1, 59.0)
	18								34.2 (28.0, 40.5)	46.2 (40.4, 52.0)	52.3 (45.9, 58.7)	53.0 (46.3, 59.8)	49.5 (41.4, 57.5)	42.6 (31.6, 53.7)
	19									44.6 (33.8, 55.5)	43.7 (33.7, 53.7)	41.0 (29.9, 52.1)	36.6 (25.3, 47.9)	30.8 (18.6, 43.0)
	20										53.4 (34.4, 72.5)	34.8 (19.1, 50.6)	23.0 (8.8, 37.3)	15.8 (4.1, 27.6)

Appendix G - Sensitivity analysis results

Figure E5 Trajectories of number of alcohol-related harm in the three years following initiation

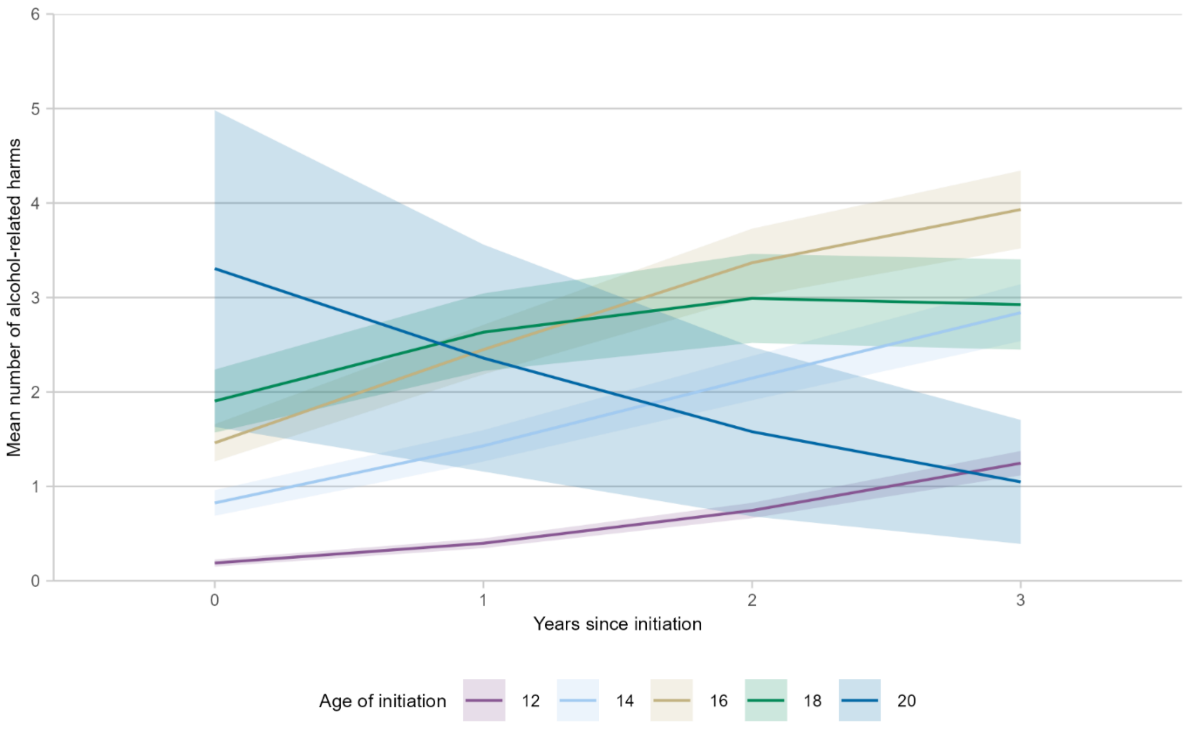

Figure E6 Trajectories of number of alcohol-related harm for all ages of initiation

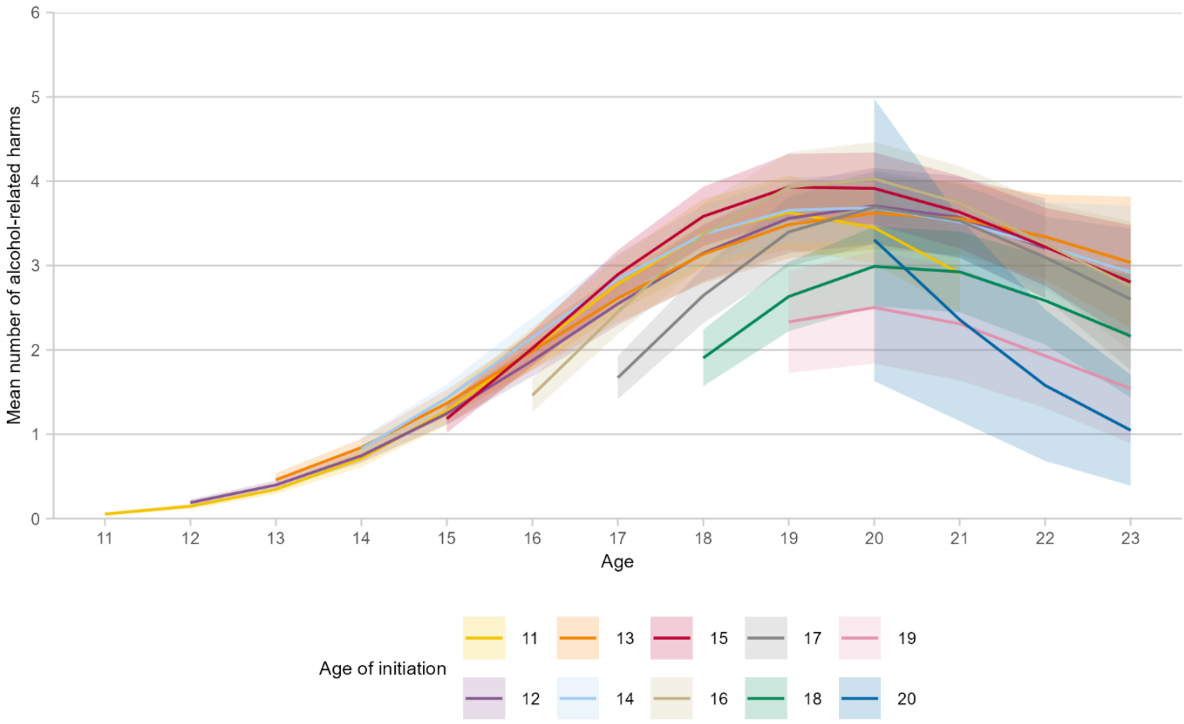

Model included linear, quadratic and cubic terms for age; and linear, quadratic, and cubic terms for time (age + age^2 + age^3 + time + $time^2$ + $time^3$). Further information on model fit is included in Appendix Table E1.

Table E4 Trajectories of number of alcohol related harms – by age of initiation

		Mean number of different alcohol-related harms (95% CI)												
		Age												
		**11**	**12**	**13**	**14**	**15**	**16**	**17**	**18**	**19**	**20**	**21**	**22**	**23**
**Age of initiation**	11	0.1 (0.0, 0.1)	0.1 (0.1, 0.2)	0.3 (0.3, 0.4)	0.7 (0.6, 0.8)	1.3 (1.1, 1.4)	2.0 (1.8, 2.3)	2.8 (2.5, 3.1)	3.4 (3.0, 3.8)	3.6 (3.2, 4.1)	3.4 (3.0, 3.9)	2.9 (2.4, 3.4)		
	12		0.2 (0.2, 0.2)	0.4 (0.3, 0.5)	0.7 (0.7, 0.8)	1.2 (1.1, 1.4)	1.9 (1.7, 2.1)	2.5 (2.3, 2.8)	3.1 (2.8, 3.5)	3.6 (3.1, 4.0)	3.7 (3.3, 4.2)	3.6 (3.1, 4.1)	3.2 (2.6, 3.8)	
	13			0.5 (0.4, 0.5)	0.8 (0.7, 0.9)	1.4 (1.2, 1.5)	2.0 (1.8, 2.2)	2.6 (2.3, 2.9)	3.1 (2.8, 3.5)	3.5 (3.1, 3.9)	3.6 (3.2, 4.1)	3.6 (3.1, 4.0)	3.3 (2.8, 3.8)	3.0 (2.3, 3.8)
	14				0.8 (0.7, 1.0)	1.4 (1.3, 1.6)	2.1 (1.9, 2.4)	2.8 (2.5, 3.1)	3.4 (3.0, 3.7)	3.7 (3.3, 4.0)	3.7 (3.3, 4.1)	3.5 (3.1, 3.9)	3.2 (2.7, 3.8)	2.9 (2.1, 3.7)
	15					1.2 (1.0, 1.3)	2.0 (1.8, 2.2)	2.9 (2.6, 3.2)	3.6 (3.2, 3.9)	3.9 (3.5, 4.3)	3.9 (3.5, 4.3)	3.6 (3.2, 4.1)	3.2 (2.8, 3.7)	2.8 (2.1, 3.5)
	16						1.5 (1.3, 1.7)	2.4 (2.2, 2.7)	3.4 (3.0, 3.7)	3.9 (3.5, 4.3)	4.0 (3.6, 4.5)	3.7 (3.3, 4.2)	3.3 (2.8, 3.7)	2.8 (2.0, 3.5)
	17							1.7 (1.4, 1.9)	2.6 (2.3, 3.0)	3.4 (3.0, 3.8)	3.7 (3.3, 4.1)	3.5 (3.1, 4.0)	3.1 (2.6, 3.6)	2.6 (1.8, 3.4)
	18								1.9 (1.6, 2.2)	2.6 (2.2, 3.0)	3.0 (2.5, 3.5)	2.9 (2.4, 3.4)	2.6 (2.1, 3.1)	2.2 (1.4, 2.9)
	19									2.3 (1.7, 2.9)	2.5 (1.8, 3.2)	2.3 (1.6, 3.0)	1.9 (1.3, 2.5)	1.5 (0.9, 2.2)
	20										3.3 (1.6, 5.0)	2.4 (1.2, 3.6)	1.6 (0.7, 2.5)	1.0 (0.4, 1.7)

Appendix G - Sensitivity analysis results

Figure E7 Trajectories of DSM-IV alcohol dependence in the three years following initiation

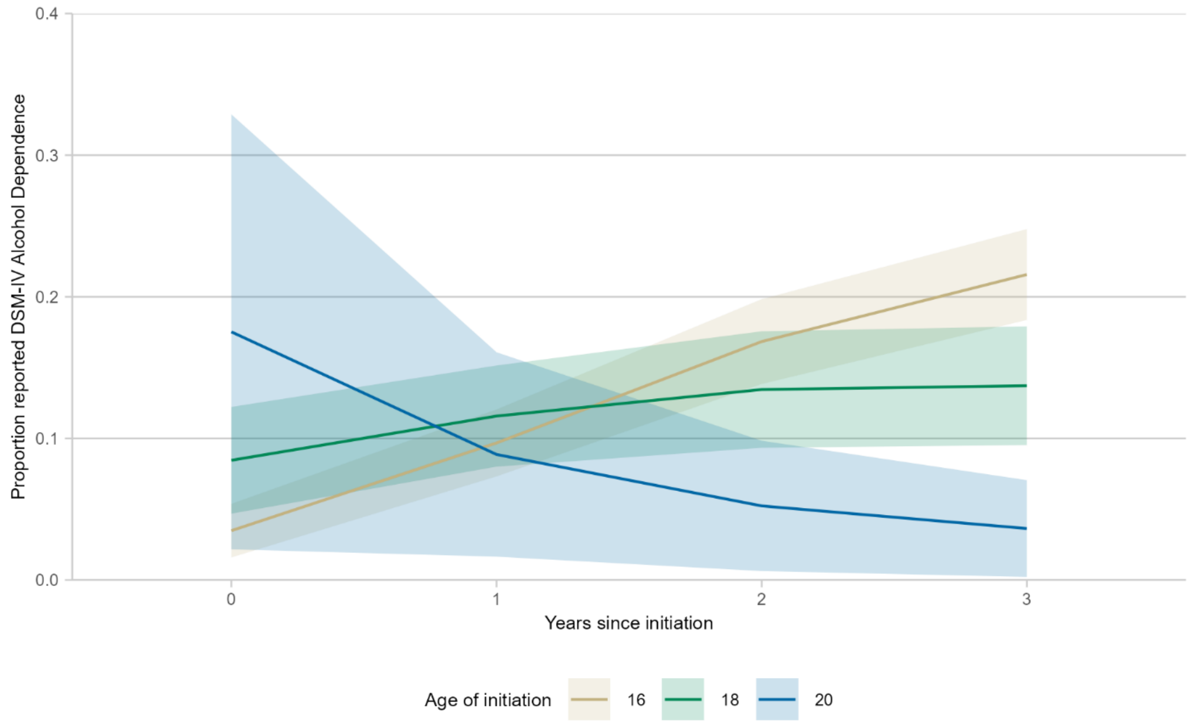

Figure E8 Trajectories of DSM-IV alcohol dependence for all ages of initiation

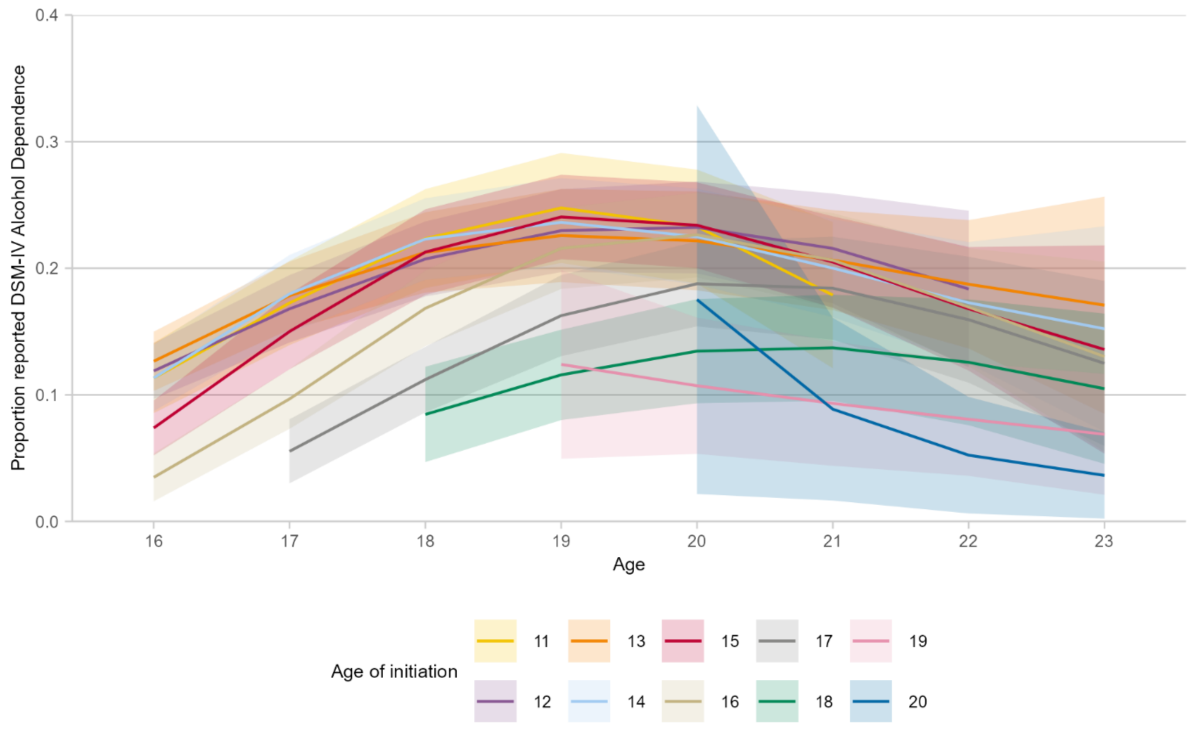

Model included linear and cubic terms for age; and linear, quadratic, and cubic terms for time (age + age^3 + time + $time^2$ + $time^3$). Further information on model fit is included in Appendix Table E1.

Appendix G - Sensitivity analysis results

Table E5 Trajectories of DSM-IV alcohol dependence – by age of initiation

		**Proportion reported symptoms consistent with DSM-IV alcohol dependence (95% CI)**							
		**Age**							
		**16**	**17**	**18**	**19**	**20**	**21**	**22**	**23**
**Age of initiation**	11	11.3 (8.6, 14.1)	17.3 (13.9, 20.6)	22.3 (18.4, 26.2)	24.8 (20.4, 29.1)	23.3 (18.8, 27.8)	17.9 (12.1, 23.6)		
	12	11.9 (9.7, 14.1)	16.8 (14.1, 19.5)	20.7 (17.8, 23.7)	23.0 (19.7, 26.2)	23.2 (19.6, 26.8)	21.6 (17.2, 25.9)	18.4 (12.2, 24.5)	
	13	12.7 (10.3, 15.0)	17.8 (15.2, 20.5)	21.3 (18.1, 24.4)	22.6 (18.9, 26.3)	22.2 (18.3, 26.1)	20.6 (16.7, 24.6)	18.7 (13.7, 23.8)	17.1 (8.5, 25.7)
	14	11.3 (8.7, 13.9)	18.0 (14.9, 21.0)	22.3 (19.1, 25.5)	23.6 (20.1, 27.1)	22.5 (18.7, 26.3)	20.0 (16.1, 23.9)	17.3 (12.5, 22.1)	15.2 (7.1, 23.3)
	15	7.4 (5.2, 9.6)	15.0 (12.0, 18.0)	21.3 (17.9, 24.7)	24.1 (20.7, 27.4)	23.4 (20.0, 26.8)	20.5 (16.9, 24.1)	16.8 (11.9, 21.7)	13.6 (5.4, 21.8)
	16	3.5 (1.6, 5.4)	9.7 (7.3, 12.0)	16.8 (13.8, 19.8)	21.6 (18.4, 24.8)	22.6 (19.3, 26.0)	20.6 (16.9, 24.3)	16.9 (12.3, 21.6)	13.0 (5.5, 20.5)
	17		5.5 (3.0, 8.1)	11.2 (8.6, 13.8)	16.3 (13.1, 19.4)	18.8 (15.4, 22.1)	18.4 (14.4, 22.5)	15.9 (11.0, 20.9)	12.5 (6.0, 19.0)
	18			8.5 (4.7, 12.2)	11.6 (8.0, 15.1)	13.4 (9.3, 17.6)	13.7 (9.5, 17.9)	12.6 (7.6, 17.5)	10.5 (4.6, 16.4)
	19				12.4 (5.0, 19.9)	10.7 (5.3, 16.1)	9.3 (4.4, 14.2)	8.1 (3.6, 12.5)	6.9 (2.1, 11.7)
	20					17.5 (2.2, 32.9)	8.9 (1.7, 16.1)	5.2 (0.6, 9.8)	3.6 (0.2, 7.1)

Appendix G - Sensitivity analysis results

Figure E9 Trajectories of DSM-IV alcohol abuse in the three years following initiation

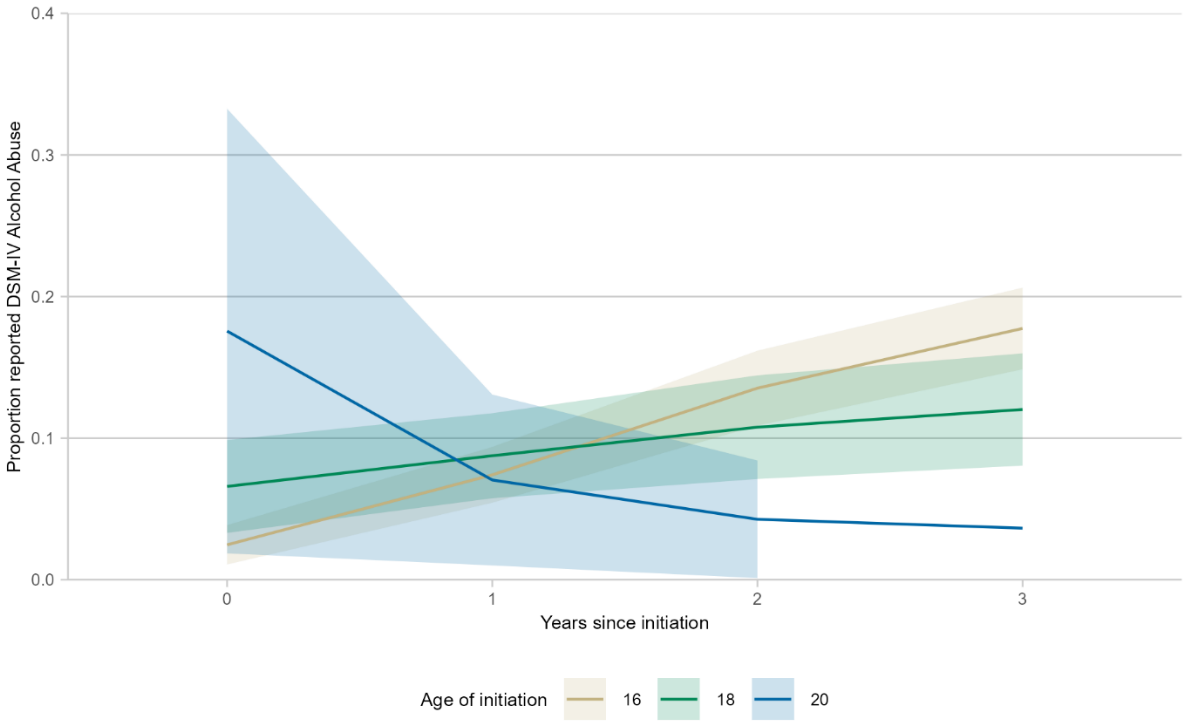

Figure E10 Trajectories of DSM-IV alcohol abuse for all ages of initiation

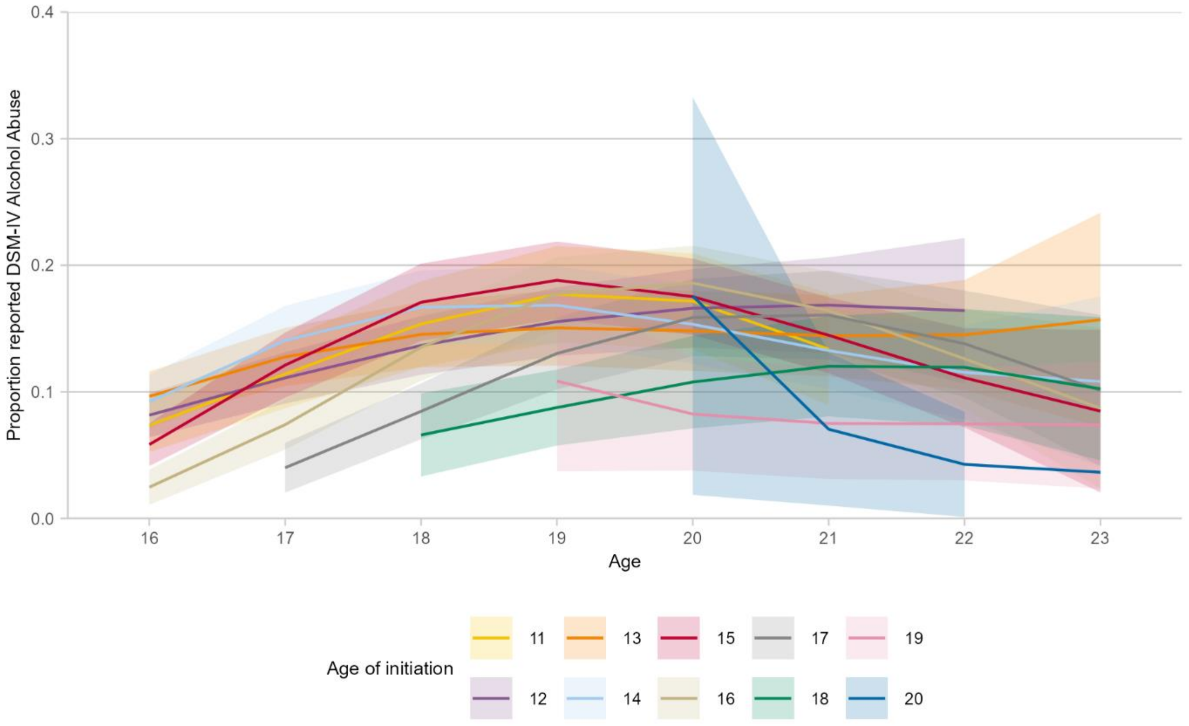

Model included linear and cubic terms for age; and linear, quadratic, and cubic terms for time (age + age^3 + time + $time^2$ + $time^3$). Further information on model fit is included in Appendix Table E1.

Table E6 Trajectories of DSM-IV alcohol abuse – by age of initiation

		Proportion reported symptoms consistent with DSM-IV alcohol abuse (95% CI)							
		Age							
		**16**	**17**	**18**	**19**	**20**	**21**	**22**	**23**
**Age of initiation**	11	7.3 (5.2, 9.4)	11.4 (8.7, 14.1)	15.4 (12.0, 18.7)	17.7 (13.9, 21.5)	17.2 (13.4, 20.9)	13.4 (9.0, 17.8)		
	12	8.2 (6.4, 9.9)	11.1 (9.1, 13.1)	13.7 (11.4, 16.0)	15.5 (12.8, 18.3)	16.6 (13.5, 19.7)	16.9 (13.1, 20.6)	16.4 (10.7, 22.1)	
	13	9.6 (7.7, 11.6)	12.8 (10.5, 15.1)	14.5 (11.9, 17.1)	15.1 (12.1, 18.0)	14.8 (11.7, 18.0)	14.4 (11.2, 17.6)	14.5 (10.2, 18.8)	15.7 (7.2, 24.1)
	14	9.2 (7.1, 11.3)	14.1 (11.3, 16.8)	16.7 (13.8, 19.6)	16.8 (13.8, 19.9)	15.3 (12.2, 18.5)	13.3 (10.2, 16.3)	11.5 (7.8, 15.2)	10.8 (4.1, 17.5)
	15	5.8 (4.1, 7.5)	12.1 (9.5, 14.7)	17.1 (14.0, 20.1)	18.8 (15.8, 21.9)	17.5 (14.5, 20.5)	14.5 (11.5, 17.5)	11.1 (7.2, 15.0)	8.5 (2.1, 14.9)
	16	2.5 (1.1, 3.9)	7.4 (5.4, 9.4)	13.5 (10.9, 16.2)	17.7 (14.9, 20.6)	18.6 (15.6, 21.5)	16.4 (13.3, 19.5)	12.6 (8.7, 16.5)	8.7 (2.4, 15.0)
	17		4.0 (2.0, 6.0)	8.5 (6.3, 10.7)	13.0 (10.2, 15.9)	15.9 (12.8, 18.9)	16.1 (12.6, 19.6)	13.8 (9.6, 18.0)	10.1 (4.1, 16.1)
	18			6.6 (3.3, 9.9)	8.8 (5.8, 11.8)	10.8 (7.1, 14.4)	12.0 (8.1, 16.0)	11.9 (7.3, 16.5)	10.2 (4.6, 15.9)
	19				10.9 (3.7, 18.0)	8.2 (3.8, 12.7)	7.5 (3.1, 11.9)	7.5 (3.0, 11.9)	7.4 (2.4, 12.4)
	20					17.6 (1.9, 33.3)	7.0 (1.0, 13.1)	4.3 (0.1, 8.4)	3.6 (-0.1, 7.4)

Figure E11 Trajectories of DSM-5 alcohol use disorder in the three years following initiation

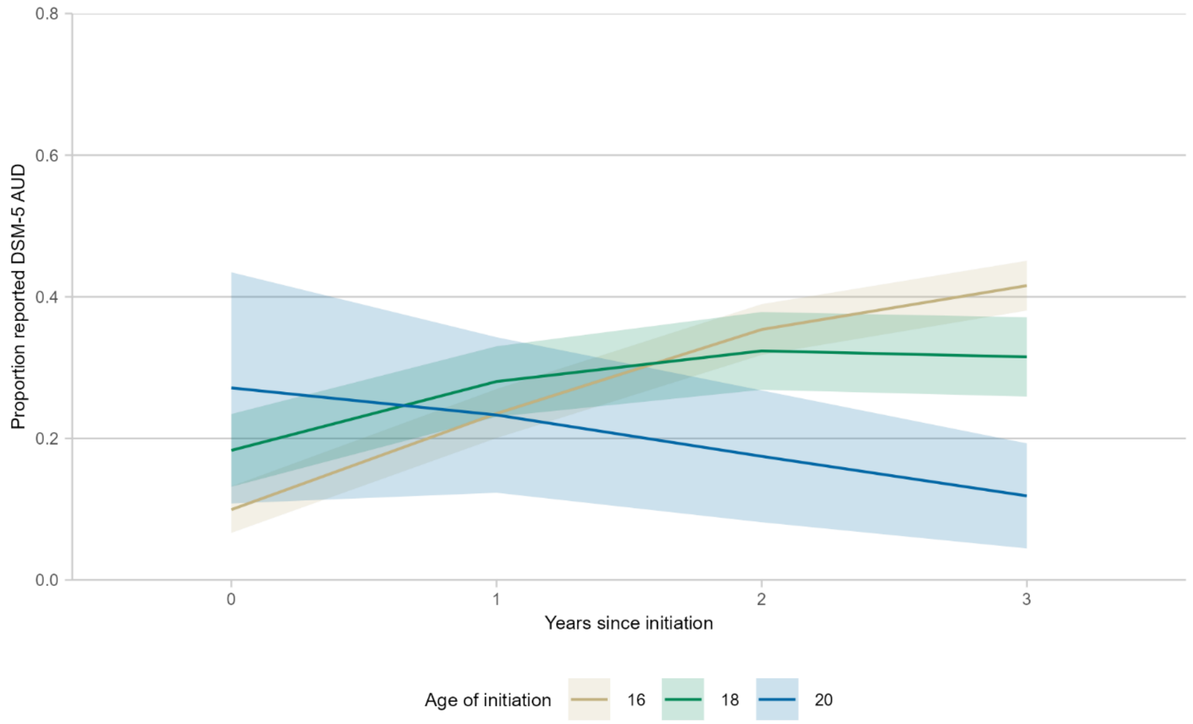

Figure E12 Trajectories of DSM-5 alcohol use disorder for all ages of initiation

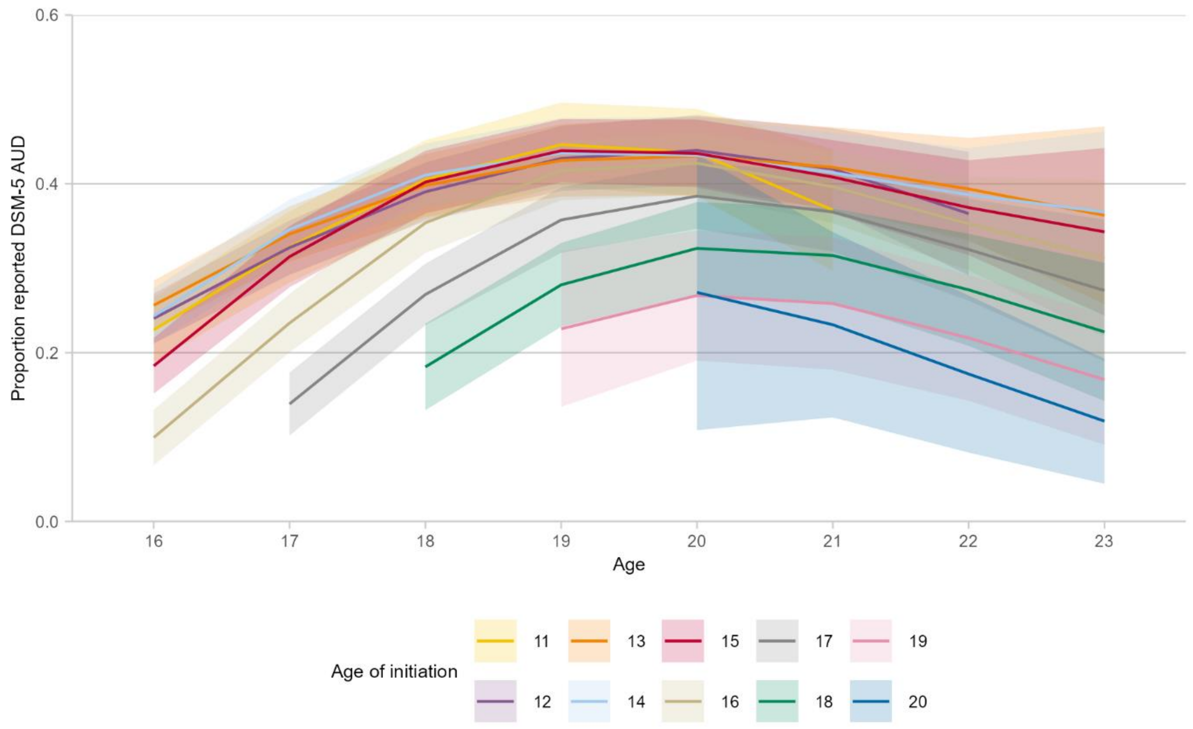

Model included linear and cubic terms for age; and linear, quadratic, and cubic terms for time (age + age^3 + time + $time^2$ + $time^3$). Further information on model fit is included in Appendix Table E1.

Appendix G - Sensitivity analysis results

Table E7 Trajectories of DSM-5 alcohol use disorder – by age of initiation

		**Proportion reported symptoms consistent with DSM-5 alcohol use disorder (95% CI)**							
		**Age**							
		**16**	**17**	**18**	**19**	**20**	**21**	**22**	**23**
**Age of initiation**	11	22.7 (18.9, 26.5)	32.5 (28.3, 36.7)	40.5 (35.8, 45.2)	44.6 (39.7, 49.6)	43.7 (38.5, 48.9)	36.9 (29.7, 44.1)		
	12	24.0 (21.1, 27.0)	32.4 (29.2, 35.7)	39.1 (35.6, 42.5)	43.0 (39.2, 46.9)	44.0 (39.8, 48.1)	41.8 (36.9, 46.6)	36.5 (29.2, 43.8)	
	13	25.6 (22.7, 28.5)	34.0 (30.8, 37.3)	39.8 (36.1, 43.5)	42.8 (38.5, 47.1)	43.3 (38.7, 47.9)	42.0 (37.2, 46.7)	39.4 (33.4, 45.4)	36.3 (25.8, 46.8)
	14	24.3 (21.1, 27.6)	34.6 (31.1, 38.1)	41.0 (37.3, 44.8)	43.7 (39.5, 47.8)	43.4 (38.8, 47.9)	41.3 (36.6, 46.0)	38.7 (33.2, 44.2)	36.7 (27.1, 46.2)
	15	18.4 (15.1, 21.7)	31.4 (27.8, 35.0)	40.2 (36.5, 43.9)	43.9 (40.2, 47.7)	43.6 (39.6, 47.6)	40.8 (36.4, 45.2)	37.2 (31.6, 42.8)	34.3 (24.4, 44.2)
	16	9.9 (6.7, 13.2)	23.5 (20.1, 26.9)	35.4 (31.8, 38.9)	41.6 (38.1, 45.1)	42.4 (38.7, 46.2)	39.7 (35.4, 44.0)	35.3 (29.8, 40.7)	31.2 (21.9, 40.5)
	17		13.9 (10.2, 17.6)	26.9 (23.3, 30.5)	35.7 (31.8, 39.6)	38.6 (34.7, 42.4)	36.7 (32.0, 41.4)	32.2 (26.2, 38.2)	27.4 (19.0, 35.7)
	18			18.3 (13.2, 23.4)	28.0 (23.1, 33.0)	32.4 (26.9, 37.8)	31.5 (25.9, 37.1)	27.4 (20.8, 34.1)	22.5 (14.3, 30.6)
	19				22.8 (13.6, 32.1)	26.8 (19.1, 34.5)	25.8 (18.0, 33.6)	21.7 (14.3, 29.2)	16.8 (9.1, 24.5)
	20					27.1 (10.8, 43.5)	23.3 (12.3, 34.3)	17.5 (8.2, 26.8)	11.9 (4.5, 19.3)

Appendix F - Additional results from secondary analysis

Table F1 Assessment of model fit for non-linear terms of age of initiation of whole drinks and age

Age of initiation	Time	Number of drinks		Monthly HED		Any HED		Number of harms		Any harms		DSM-IV Dependence		DSM-IV Abuse		DSM-5 AUD	
		AIC	BIC	AIC	BIC	AIC	BIC	AIC	BIC	AIC	BIC	AIC	BIC	AIC	BIC	AIC	BIC
x	z	79289	79490	7824	8018	6859	7054	28351	28552	7510	7705	4847	5035	4014	4202	6849	7037
x + x^2	z	79195	79410	7801	8010	6825	7033	28275	28490	7448	7656	4847	5048	4011	4212	6844	7045
x + x^3	z	79198	79413	7797	8006	6816	7025	28281	28496	7445	7654	4848	5049	4013	4214	6844	7045
x + x^2 + x^3	z	79195	79424	7797	8019	6808	7030	28271	28500	7442	7664	4841	5056	4009	4223	6832	7047
x	z + z^2	78395	78610	7429	7637	6467	6675	27610	27826	7216	7425	4783	4984	3962	4163	6739	6940
x + x^2	z + z^2	78259	**78495**	7339	7568	6375	6604	27467	27703	7108	7338	4749	**4970**	3931	4152	6669	6890
x + x^3	z + z^2	78261	78497	7336	7566	6366	6596	27480	27717	7105	7335	4750	4971	3930	**4151**	6668	**6889**
x + x^2 + x^3	z + z^2	78253	78510	7324	7574	6351	6602	27437	**27694**	7093	7343	4746	4988	3923	4164	6667	6909
x	z + z^3	78429	78644	7454	7662	6459	6667	27657	27872	7207	7415	4789	4990	3969	4170	6752	6953
x + x^2	z + z^3	78291	78527	7362	7591	6366	6595	27510	27746	7094	7324	4760	4981	3939	4160	6692	6913
x + x^3	z + z^3	78294	78530	7360	7590	6358	6588	27523	27759	7092	7321	4761	4982	3940	4161	6689	6910
x + x^2 + x^3	z + z^3	78288	78545	7347	7597	6342	6592	27484	27741	7078	7328	4756	4997	3929	4170	6686	6927
x	z + z^2 + z^3	78390	78619	7418	7640	6442	6665	27600	27829	7177	7400	4785	5000	3964	4178	6738	6952
x + x^2	z + z^2 + z^3	78240	78497	7301	7551	6343	6593	27447	27704	7063	7313	**4739**	4980	**3919**	4160	**6652**	6893
x + x^3	z + z^2 + z^3	**78239**	78496	**7299**	**7549**	6331	**6581**	27457	27714	**7057**	**7307**	4742	4983	3921	4162	6656	6897
x + x^2 + x^3	z + z^2 + z^3	78239	78524	7303	7581	**6331**	6608	**27436**	27721	7062	7340	4742	5010	3924	4192	6656	6924

Note: best performing model based on each criteria is highlighted in bold. Final model was chosen based on AIC to prioritise predictions, because results are based on marginal predicted means based on the models, not the model parameters themselves.

Appendix F - Additional results from secondary analysis

Table F2 Comparison of outcomes at age 18 based on age of initiation

	RR / IRR (95% CI)					
**Age of initiation**	**Number of drinks***	**At least monthly HED**	**Number of harms***	**DSM-IV Dependence**	**DSM-IV Abuse**	**DSM-5 AUD**
12	2.56 (1.62, 4.05)	2.52 (2.18, 2.91)	1.82 (1.38, 2.39)	5.38 (3.80, 7.63)	4.96 (2.87, 8.55)	3.30 (2.41, 4.52)
13	3.25 (2.45, 4.31)	2.25 (1.94, 2.61)	2.31 (1.93, 2.77)	4.15 (2.96, 5.81)	4.51 (2.80, 7.27)	3.05 (2.25, 4.13)
14	3.38 (2.66, 4.30)	1.94 (1.68, 2.25)	2.35 (2.01, 2.75)	3.20 (2.31, 4.44)	3.89 (2.50, 6.04)	2.91 (2.16, 3.91)
15	2.90 (2.34, 3.59)	1.63 (1.42, 1.88)	2.11 (1.84, 2.43)	2.45 (1.79, 3.34)	3.17 (2.07, 4.87)	2.70 (2.01, 3.64)
16	2.11 (1.74, 2.56)	1.35 (1.20, 1.53)	1.75 (1.55, 1.98)	1.83 (1.40, 2.40)	2.42 (1.59, 3.67)	2.30 (1.72, 3.09)
17	1.39 (1.18, 1.62)	1.14 (1.05, 1.23)	1.33 (1.20, 1.47)	1.35 (1.14, 1.60)	1.67 (1.20, 2.34)	1.68 (1.31, 2.15)
18	REF	REF	REF	REF	REF	REF

Appendix F - Additional results from secondary analysis

Table F3 Comparison of outcomes at age 20 based on age of initiation

	**RR / IRR (95% CI)**					
**Age of initiation**	**Number of drinks***	**At least monthly HED**	**Number of harms***	**DSM-IV Dependence**	**DSM-IV Abuse**	**DSM-5 AUD**
12	2.56 (1.54, 4.23)	1.31 (1.00, 1.73)	3.31 (2.44, 4.51)	1.79 (1.61, 1.98)	1.54 (1.04, 2.29)	1.74 (1.40, 2.16)
13	2.67 (1.98, 3.60)	1.63 (1.36, 1.95)	2.57 (1.96, 3.38)	1.65 (1.49, 1.83)	1.38 (0.92, 2.05)	1.57 (1.29, 1.91)
14	2.58 (2.04, 3.26)	1.69 (1.46, 1.95)	2.10 (1.65, 2.67)	1.51 (1.36, 1.67)	1.42 (0.95, 2.13)	1.47 (1.21, 1.78)
15	2.22 (1.81, 2.72)	1.51 (1.33, 1.72)	1.77 (1.45, 2.15)	1.37 (1.25, 1.49)	1.63 (1.10, 2.40)	1.40 (1.16, 1.69)
16	1.77 (1.49, 2.10)	1.32 (1.19, 1.46)	1.49 (1.29, 1.73)	1.23 (1.15, 1.31)	1.73 (1.22, 2.44)	1.31 (1.11, 1.55)
17	1.34 (1.20, 1.51)	1.16 (1.08, 1.24)	1.24 (1.13, 1.36)	1.11 (1.07, 1.15)	1.47 (1.17, 1.85)	1.17 (1.05, 1.31)
18	REF	REF	REF	REF	REF	REF
19	0.83 (0.67, 1.03)	0.92 (0.81, 1.04)	0.79 (0.68, 0.92)	0.93 (0.87, 0.98)	0.76 (0.56, 1.05)	0.89 (0.75, 1.06)
20	1.19 (0.60, 2.36)	1.42 (0.97, 2.07)	0.62 (0.42, 0.92)	0.90 (0.78, 1.04)	1.62 (0.65, 4.03)	1.00 (0.55, 1.80)

Appendix F - Additional results from secondary analysis

Figure F1 Trajectories of alcohol consumption in the three years following initiation of whole drinks

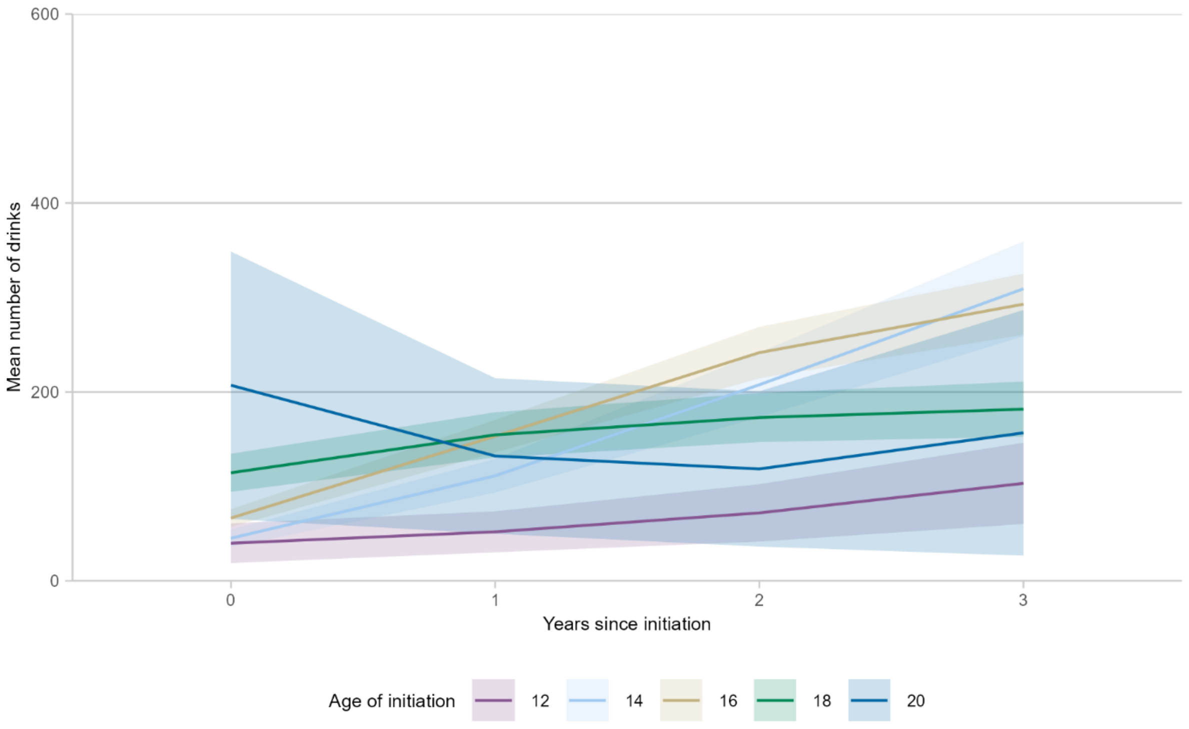

Figure F2 Trajectories of alcohol consumption for all ages of initiation of whole drinks

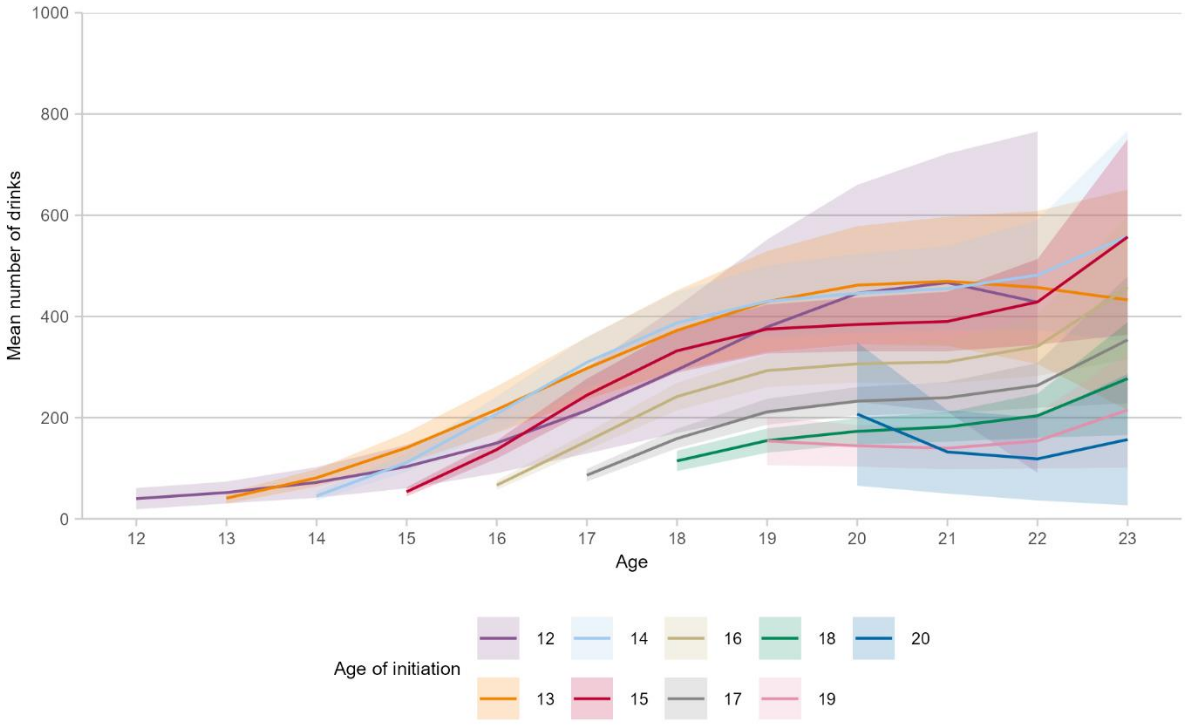

Model included linear and cubic terms for age; and linear, quadratic, and cubic terms for time (age + age^3 + time + $time^2$ + $time^3$). Further information on model fit is included in Appendix Table F1.

Table F4 Trajectories of alcohol consumption– by age of initiation of whole drinks

		**Mean number of drinks (95% CI)**											
		**Age**											
		**12**	**13**	**14**	**15**	**16**	**17**	**18**	**19**	**20**	**21**	**22**	**23**
	12	39.9 (18.9, 60.8)	51.9 (30.3, 73.6)	72.0 (41.7, 102.2)	103.3 (60.4, 146.1)	149.7 (90.2, 209.2)	214.0 (129.0, 299.1)	294.3 (169.7, 418.9)	379.1 (206.4, 551.8)	445.6 (231.3, 659.9)	466.8 (211.9, 721.7)	428.2 (91.0, 765.4)	
	13		40.8 (30.2, 51.3)	81.0 (63.8, 98.1)	140.5 (110.2, 170.8)	215.8 (169.9, 261.7)	297.3 (236.0, 358.7)	372.3 (293.1, 451.5)	429.2 (329.5, 529.0)	461.7 (345.2, 578.2)	469.4 (342.0, 596.9)	457.2 (306.2, 608.1)	432.7 (215.2, 650.2)
	14			45.2 (36.2, 54.2)	111.2 (93.7, 128.8)	207.6 (173.6, 241.6)	309.2 (259.1, 359.2)	387.2 (326.1, 448.4)	429.9 (359.6, 500.1)	445.7 (368.2, 523.2)	454.9 (371.4, 538.4)	481.7 (373.3, 590.2)	558.3 (350.1, 766.5)
	15				53.4 (44.4, 62.4)	136.8 (119.0, 154.6)	244.4 (211.9, 276.8)	332.1 (289.1, 375.1)	375.0 (326.5, 423.4)	384.2 (331.3, 437.1)	390.0 (331.2, 448.9)	428.6 (343.9, 513.2)	557.1 (364.0, 750.1)
**Age of initiation**	16					66.5 (57.3, 75.7)	153.2 (136.2, 170.1)	241.6 (214.5, 268.7)	292.9 (260.7, 325.1)	306.3 (269.6, 343.0)	310.0 (268.1, 352.0)	340.9 (282.7, 399.1)	457.3 (315.5, 599.1)
	17						86.2 (73.8, 98.6)	158.7 (139.5, 177.8)	211.5 (185.9, 237.1)	232.4 (204.6, 260.2)	239.6 (208.6, 270.5)	263.7 (219.5, 307.9)	353.6 (228.4, 478.9)
	18							114.5 (94.4, 134.5)	154.6 (130.7, 178.4)	172.9 (147.0, 198.8)	181.7 (152.6, 210.9)	203.7 (160.3, 247.2)	277.1 (165.5, 388.7)
	19								153.9 (106.3, 201.4)	144.3 (103.3, 185.4)	139.5 (98.1, 181.0)	153.9 (99.4, 208.4)	215.1 (101.6, 328.6)
	20									207.0 (65.6, 348.5)	132.2 (49.9, 214.5)	118.5 (36.4, 200.6)	156.7 (26.8, 286.7)

Figure F3 Trajectories of at least monthly heavy episodic drinking in the three years following initiation of whole drinks

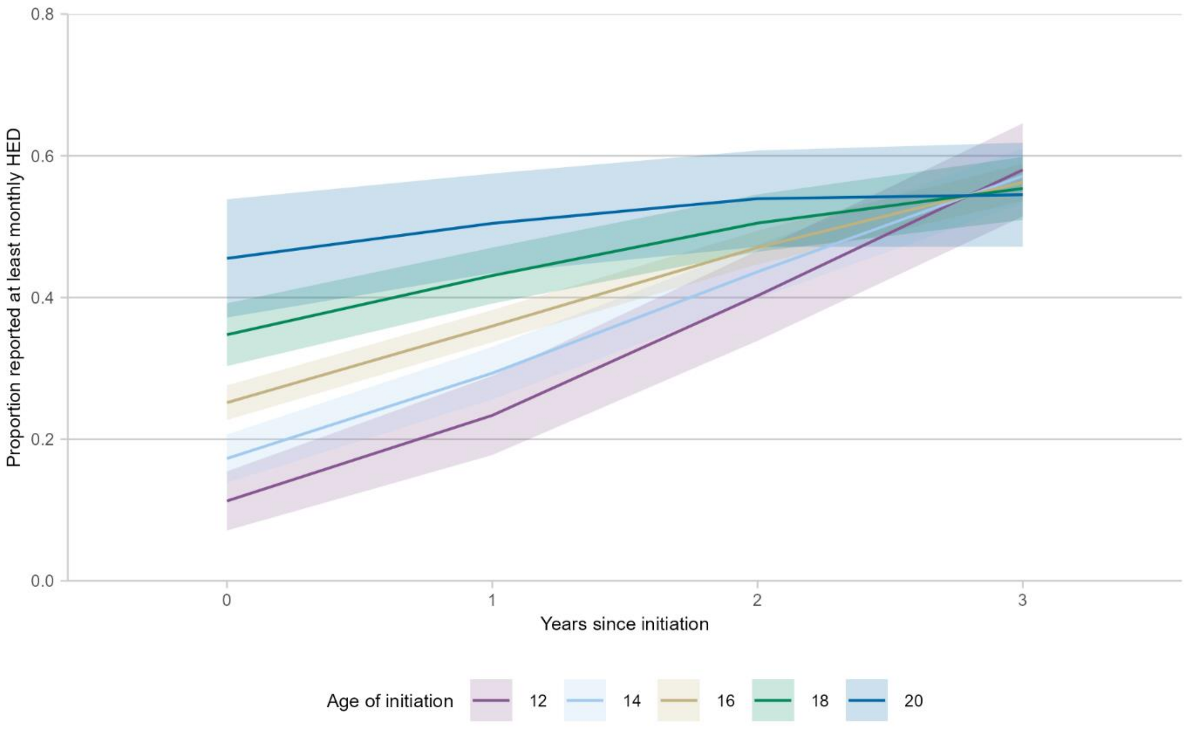

Figure F4 Trajectories of at least monthly heavy episodic drinking for all ages of initiation of whole drinks

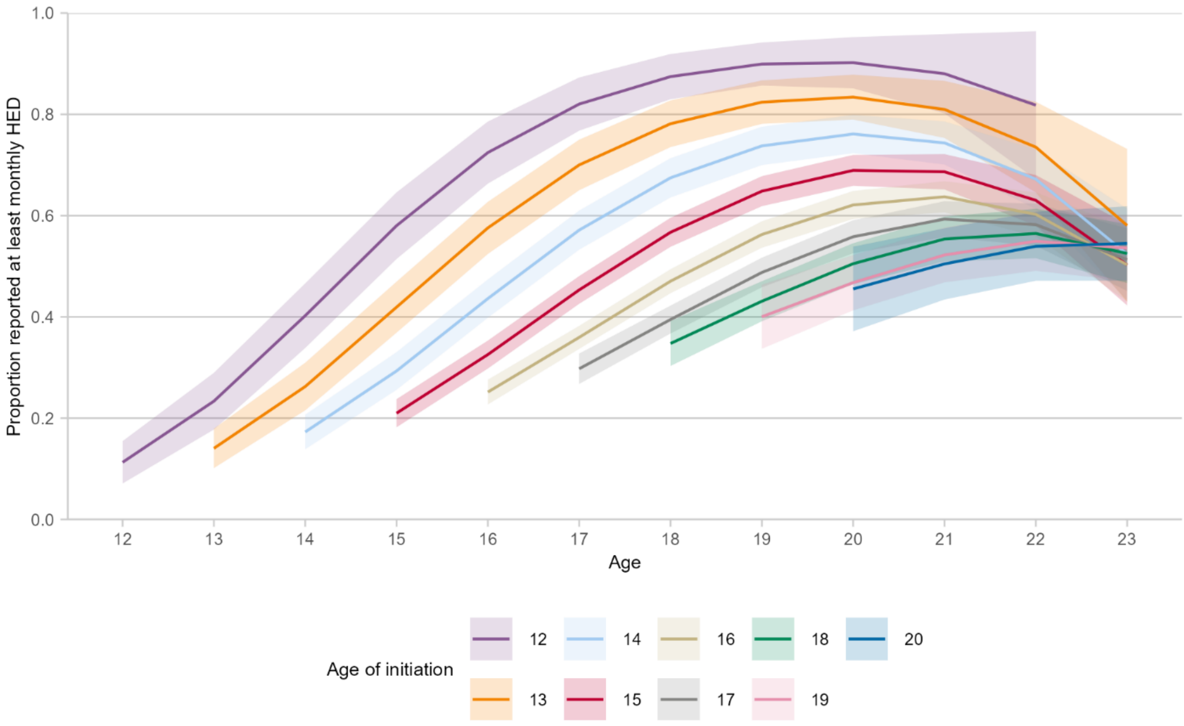

Model included linear and cubic terms for age; and linear, quadratic, and cubic terms for time (age + age^3 + time + $time^2$ + $time^3$). Further information on model fit is included in Appendix Table F1.

Table F5 Trajectories of at least monthly heavy episodic drinking – by age of initiation of whole drinks

		**Proportion reported monthly heavy episodic drinking (95% CI)**											
		**Age**											
		**12**	**13**	**14**	**15**	**16**	**17**	**18**	**19**	**20**	**21**	**22**	**23**
**Age of initiation**	12	11.3 (7.1, 15.4)	23.4 (17.8, 28.9)	40.2 (33.9, 46.6)	58.0 (51.4, 64.6)	72.4 (66.3, 78.6)	82.0 (76.8, 87.3)	87.4 (83.0, 91.9)	89.9 (85.7, 94.2)	90.2 (85.1, 95.2)	88.0 (80.2, 95.8)	81.8 (67.2, 96.4)	
	13		14.0 (10.2, 17.9)	26.2 (21.5, 30.9)	41.9 (36.9, 46.9)	57.6 (52.4, 62.7)	70.0 (65.0, 75.0)	78.1 (73.5, 82.7)	82.4 (78.1, 86.7)	83.4 (79.0, 87.8)	81.0 (75.3, 86.6)	73.5 (64.6, 82.5)	58.1 (43.0, 73.2)
	14			17.3 (13.9, 20.7)	29.3 (25.6, 33.0)	43.6 (39.9, 47.4)	57.1 (53.3, 61.0)	67.5 (63.6, 71.3)	73.8 (70.0, 77.5)	76.1 (72.3, 79.9)	74.4 (70.1, 78.6)	67.2 (61.4, 73.0)	52.4 (43.4, 61.3)
	15				21.0 (18.2, 23.8)	32.6 (29.8, 35.3)	45.3 (42.6, 48.1)	56.7 (53.8, 59.6)	64.8 (61.9, 67.8)	68.9 (65.9, 72.0)	68.7 (65.2, 72.1)	63.0 (58.0, 68.0)	50.3 (42.3, 58.2)
	16					25.2 (22.7, 27.6)	36.0 (33.7, 38.2)	47.0 (44.7, 49.4)	56.3 (53.7, 58.9)	62.1 (59.3, 64.9)	63.7 (60.7, 66.8)	60.2 (55.9, 64.6)	50.3 (43.0, 57.5)
	17						29.8 (26.8, 32.7)	39.5 (36.7, 42.2)	48.8 (45.9, 51.7)	55.8 (52.6, 59.1)	59.3 (55.8, 62.8)	58.2 (54.1, 62.4)	51.3 (45.2, 57.4)
	18							34.7 (30.3, 39.2)	43.1 (39.1, 47.0)	50.5 (46.5, 54.5)	55.4 (51.0, 59.8)	56.5 (51.6, 61.3)	52.5 (46.7, 58.3)
	19								40.0 (33.7, 46.3)	46.8 (41.3, 52.2)	52.2 (46.8, 57.6)	54.9 (49.1, 60.8)	53.6 (47.1, 60.1)
	20									45.5 (37.2, 53.9)	50.5 (43.4, 57.5)	53.9 (47.1, 60.7)	54.5 (47.2, 61.8)

Figure F5 Trajectories of number of alcohol-related harm in the three years following initiation of whole drinks

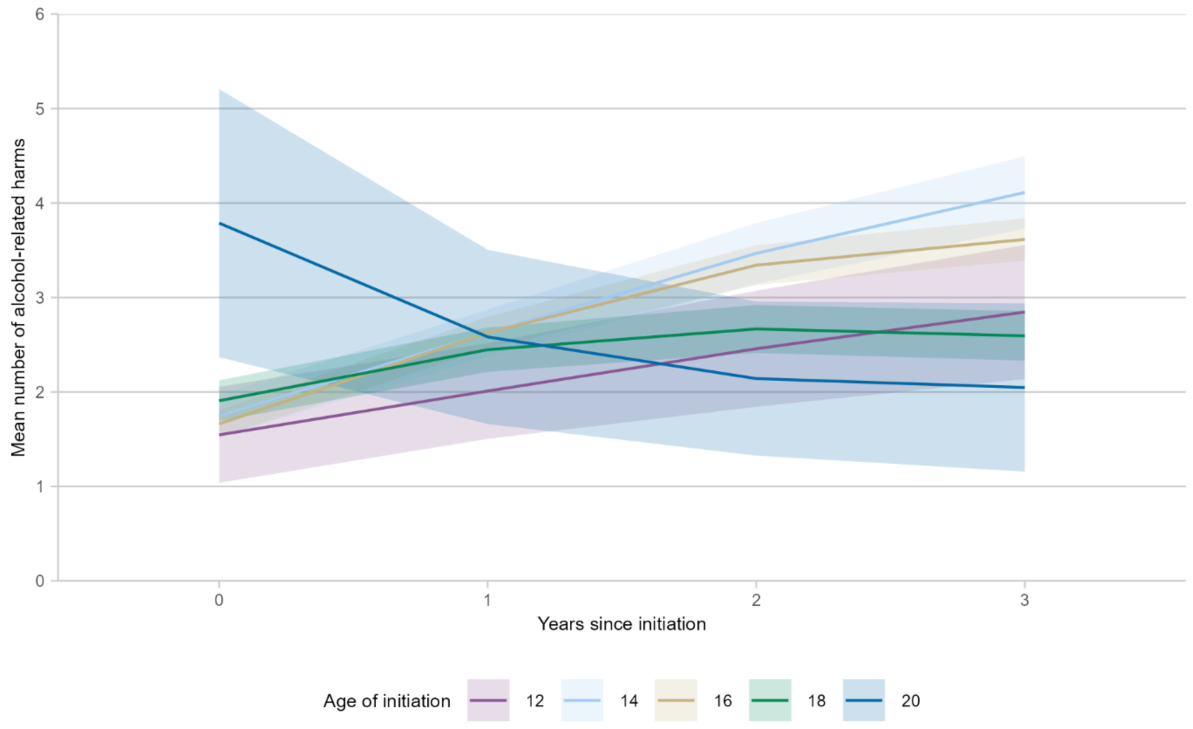

Figure F6 Trajectories of number of alcohol-related harm for all ages of initiation of whole drinks

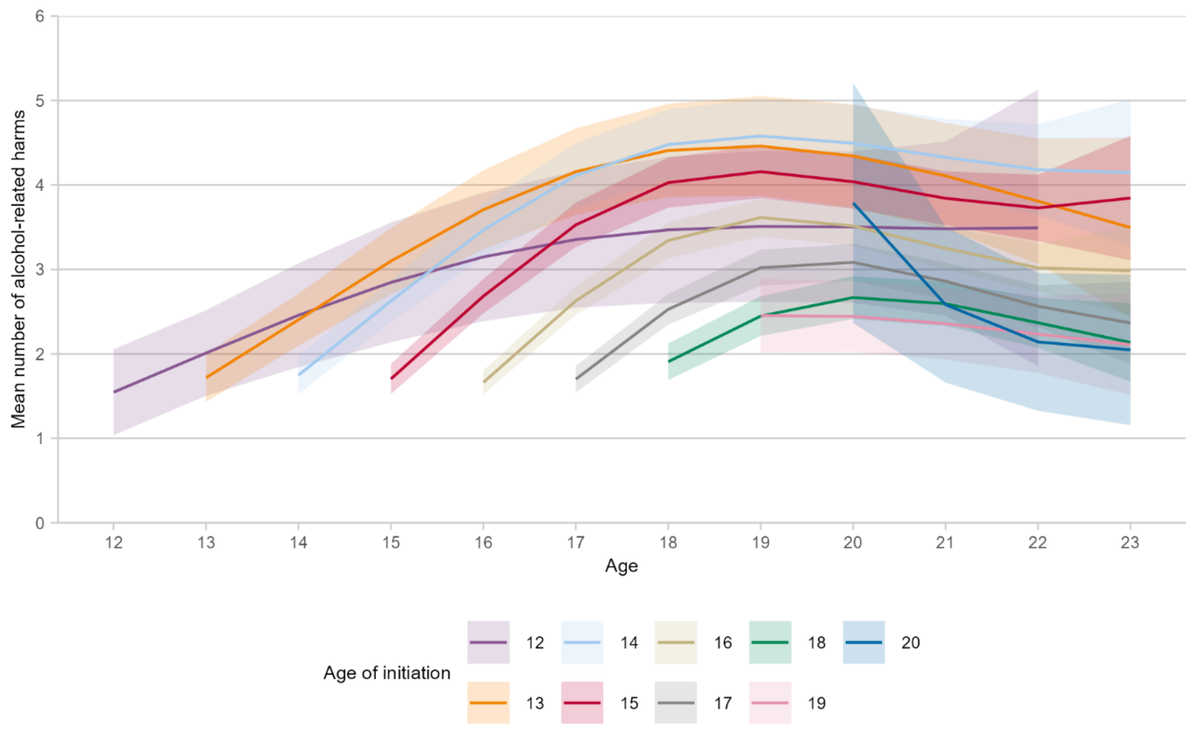

Model included linear, quadratic and cubic terms for age; and linear, quadratic, and cubic terms for time (age + age^2 + age^3 + time + $time^2$ + $time^3$). Further information on model fit is included in Appendix Table F1.

Table F6 Trajectories of number of alcohol related harms – by age of initiation of whole drinks

		Mean number of different alcohol-related harms (95% CI)											
		**Age**											
		**12**	**13**	**14**	**15**	**16**	**17**	**18**	**19**	**20**	**21**	**22**	**23**
	12	1.5 (1.0, 2.1)	2.0 (1.5, 2.5)	2.5 (1.8, 3.1)	2.8 (2.1, 3.6)	3.1 (2.4, 3.9)	3.4 (2.5, 4.2)	3.5 (2.6, 4.3)	3.5 (2.6, 4.4)	3.5 (2.6, 4.4)	3.5 (2.5, 4.5)	3.5 (1.9, 5.1)	
	13		1.7 (1.4, 2.0)	2.4 (2.1, 2.7)	3.1 (2.7, 3.5)	3.7 (3.2, 4.2)	4.2 (3.6, 4.7)	4.4 (3.9, 5.0)	4.5 (3.9, 5.1)	4.3 (3.7, 5.0)	4.1 (3.5, 4.7)	3.8 (3.1, 4.5)	3.5 (2.4, 4.6)
	14			1.7 (1.5, 2.0)	2.6 (2.4, 2.9)	3.5 (3.1, 3.8)	4.1 (3.7, 4.5)	4.5 (4.1, 4.9)	4.6 (4.1, 5.0)	4.5 (4.0, 4.9)	4.3 (3.9, 4.8)	4.2 (3.6, 4.7)	4.1 (3.3, 5.0)
	15				1.7 (1.5, 1.9)	2.7 (2.5, 2.9)	3.5 (3.3, 3.8)	4.0 (3.7, 4.3)	4.2 (3.8, 4.5)	4.0 (3.7, 4.4)	3.8 (3.5, 4.2)	3.7 (3.3, 4.1)	3.8 (3.1, 4.6)
**Age of initiation**	16					1.7 (1.5, 1.8)	2.6 (2.5, 2.8)	3.3 (3.1, 3.6)	3.6 (3.4, 3.8)	3.5 (3.3, 3.8)	3.2 (3.0, 3.5)	3.0 (2.7, 3.3)	3.0 (2.5, 3.5)
	17						1.7 (1.5, 1.9)	2.5 (2.3, 2.7)	3.0 (2.8, 3.2)	3.1 (2.9, 3.3)	2.9 (2.6, 3.1)	2.6 (2.3, 2.8)	2.4 (1.9, 2.8)
	18							1.9 (1.7, 2.1)	2.4 (2.2, 2.7)	2.7 (2.4, 2.9)	2.6 (2.3, 2.9)	2.4 (2.1, 2.7)	2.1 (1.7, 2.6)
	19								2.5 (2.0, 2.9)	2.4 (2.0, 2.9)	2.4 (1.9, 2.8)	2.2 (1.8, 2.7)	2.1 (1.5, 2.7)
	20									3.8 (2.4, 5.2)	2.6 (1.7, 3.5)	2.1 (1.3, 3.0)	2.0 (1.2, 2.9)

Figure F7 Trajectories of DSM-IV alcohol dependence in the three years following initiation of whole drinks

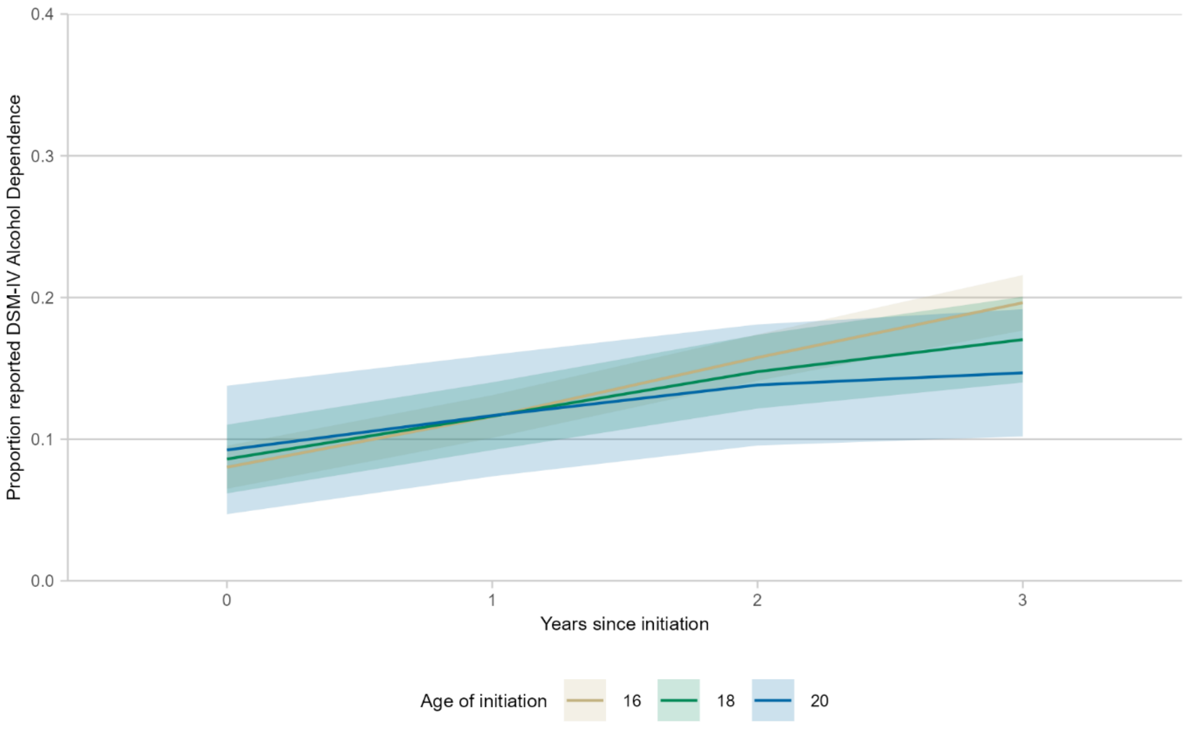

Figure F8 Trajectories of DSM-IV alcohol dependence for all ages of initiation of whole drinks

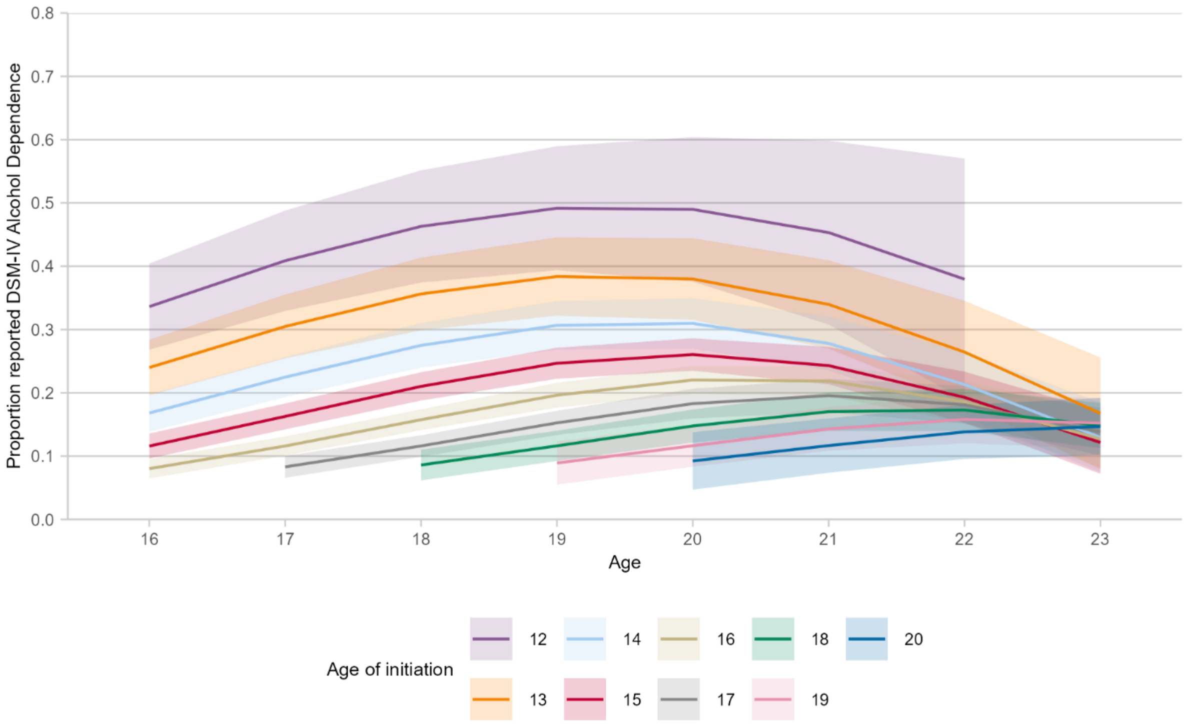

Model included linear and cubic terms for age; and linear, quadratic, and cubic terms for time (age + age^3 + time + $time^2$ + $time^3$). Further information on model fit is included in Appendix Table F1.

Table F7 Trajectories of DSM-IV alcohol dependence – by age of initiation of whole drinks

		Proportion reported symptoms consistent with DSM-IV alcohol dependence (95% CI)							
		Age							
		**16**	**17**	**18**	**19**	**20**	**21**	**22**	**23**
**Age of initiation**	12	33.6 (26.8, 40.4)	40.9 (33.0, 48.8)	46.3 (37.4, 55.1)	49.2 (39.4, 59.0)	49.0 (37.6, 60.4)	45.3 (30.8, 59.8)	38.0 (18.9, 57.0)	
	13	24.0 (19.6, 28.4)	30.5 (25.5, 35.5)	35.6 (29.9, 41.3)	38.4 (32.2, 44.6)	38.0 (31.6, 44.4)	34.0 (27.0, 40.9)	26.4 (18.3, 34.5)	16.8 (7.9, 25.6)
	14	16.8 (13.9, 19.7)	22.5 (19.4, 25.6)	27.5 (24.0, 31.0)	30.7 (26.8, 34.5)	31.0 (27.0, 34.9)	27.8 (23.6, 32.0)	21.3 (16.3, 26.3)	13.0 (7.5, 18.4)
	15	11.6 (9.6, 13.6)	16.3 (14.2, 18.3)	21.0 (18.8, 23.3)	24.7 (22.2, 27.1)	26.1 (23.5, 28.6)	24.3 (21.4, 27.2)	19.3 (15.2, 23.3)	12.1 (7.2, 17.1)
	16	8.0 (6.5, 9.5)	11.6 (10.1, 13.1)	15.8 (14.1, 17.4)	19.6 (17.7, 21.6)	22.0 (19.9, 24.2)	21.9 (19.5, 24.2)	18.6 (15.3, 21.8)	12.7 (8.3, 17.1)
	17		8.3 (6.6, 10.0)	11.6 (9.9, 13.3)	15.3 (13.4, 17.2)	18.3 (16.0, 20.6)	19.6 (16.9, 22.2)	18.1 (15.2, 21.0)	13.8 (10.1, 17.6)
	18			8.6 (6.2, 11.0)	11.6 (9.2, 14.0)	14.8 (12.2, 17.4)	17.0 (14.0, 20.1)	17.3 (14.0, 20.6)	14.8 (11.3, 18.4)
	19				8.9 (5.5, 12.3)	11.6 (8.3, 14.9)	14.3 (10.9, 17.7)	15.8 (12.0, 19.6)	15.2 (11.2, 19.2)
	20					9.2 (4.7, 13.8)	11.7 (7.4, 15.9)	13.8 (9.6, 18.1)	14.7 (10.2, 19.2)

Figure F9 Trajectories of DSM-IV alcohol abuse in the three years following initiation of whole drinks

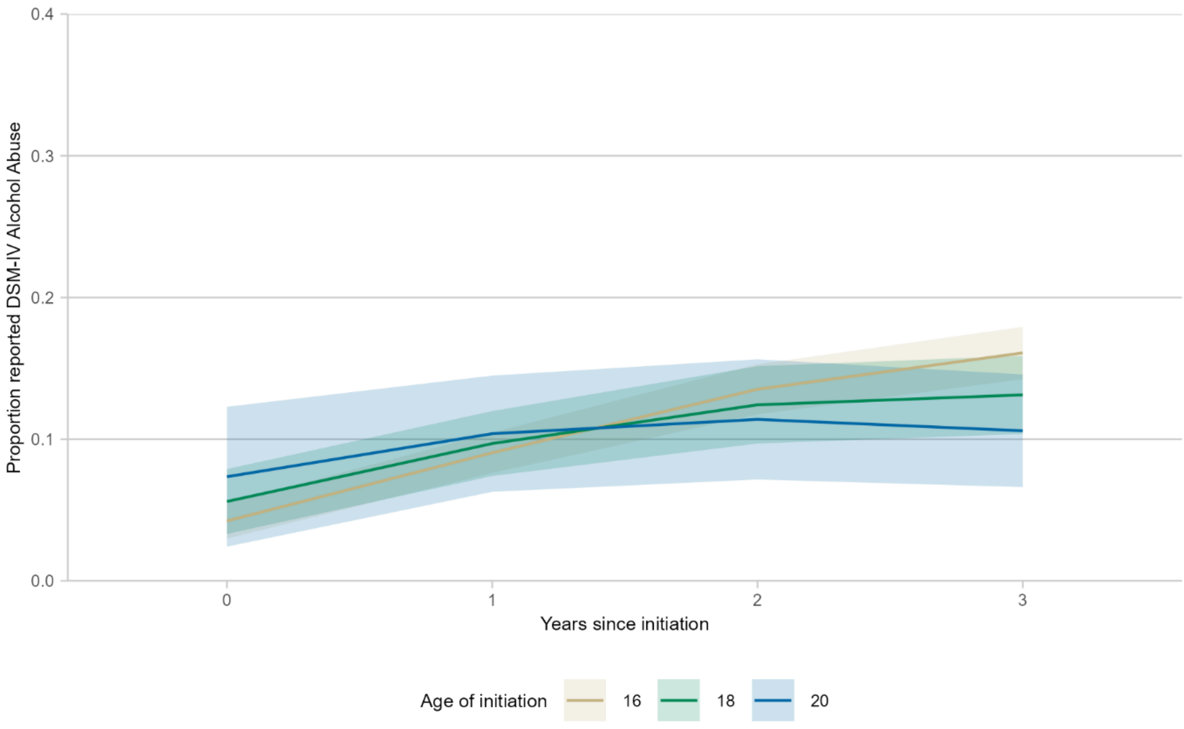

Figure F10 Trajectories of DSM-IV alcohol abuse for all ages of initiation of whole drinks

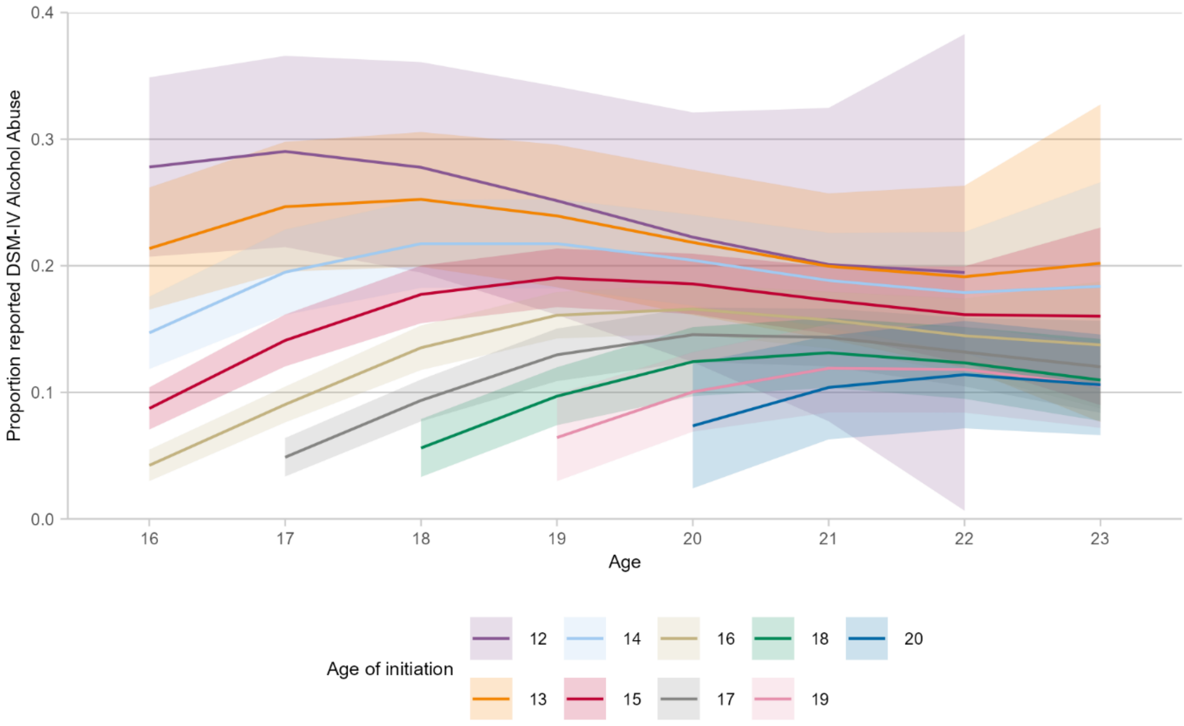

Model included linear and cubic terms for age; and linear, quadratic, and cubic terms for time (age + age^3 + time + $time^2$ + $time^3$). Further information on model fit is included in Appendix Table F1.

Table F8 Trajectories of DSM-IV alcohol abuse – by age of initiation of whole drinks

		Proportion reported symptoms consistent with DSM-IV alcohol abuse (95% CI)							
		Age							
		**16**	**17**	**18**	**19**	**20**	**21**	**22**	**23**
**Age of initiation**	12	27.8 (20.7, 34.9)	29.0 (21.5, 36.6)	27.8 (19.5, 36.1)	25.1 (16.1, 34.2)	22.3 (12.4, 32.1)	20.1 (7.7, 32.5)	19.5 (0.7, 38.3)	
	13	21.4 (16.5, 26.2)	24.7 (19.5, 29.8)	25.2 (19.9, 30.6)	23.9 (18.3, 29.6)	21.8 (16.1, 27.6)	20.0 (14.2, 25.7)	19.1 (11.9, 26.3)	20.2 (7.7, 32.7)
	14	14.7 (11.9, 17.5)	19.5 (16.1, 22.9)	21.7 (18.3, 25.2)	21.7 (18.2, 25.3)	20.4 (16.8, 24.0)	18.8 (15.1, 22.6)	17.9 (13.1, 22.7)	18.4 (10.2, 26.6)
	15	8.7 (7.1, 10.4)	14.1 (12.1, 16.1)	17.7 (15.5, 20.0)	19.0 (16.7, 21.4)	18.6 (16.2, 20.9)	17.3 (14.6, 19.9)	16.1 (12.3, 20.0)	16.0 (9.0, 23.0)
	16	4.2 (3.0, 5.5)	9.0 (7.6, 10.4)	13.5 (11.8, 15.3)	16.1 (14.3, 17.9)	16.6 (14.6, 18.5)	15.7 (13.5, 17.9)	14.5 (11.5, 17.4)	13.7 (8.7, 18.7)
	17		4.9 (3.4, 6.4)	9.4 (7.7, 11.0)	13.0 (10.9, 15.0)	14.6 (12.4, 16.7)	14.3 (12.1, 16.6)	13.2 (10.5, 15.9)	12.0 (8.4, 15.6)
	18			5.6 (3.3, 7.9)	9.7 (7.4, 12.0)	12.4 (9.7, 15.1)	13.1 (10.4, 15.9)	12.3 (9.5, 15.2)	11.0 (7.7, 14.2)
	19				6.4 (3.0, 9.9)	10.0 (6.9, 13.2)	11.9 (8.4, 15.4)	11.8 (8.4, 15.2)	10.5 (7.2, 13.9)
	20					7.4 (2.4, 12.3)	10.4 (6.3, 14.5)	11.4 (7.2, 15.6)	10.6 (6.6, 14.6)

Appendix F - Additional results from secondary analysis

Figure F11 Trajectories of DSM-5 alcohol use disorder in the three years following initiation of whole drinks

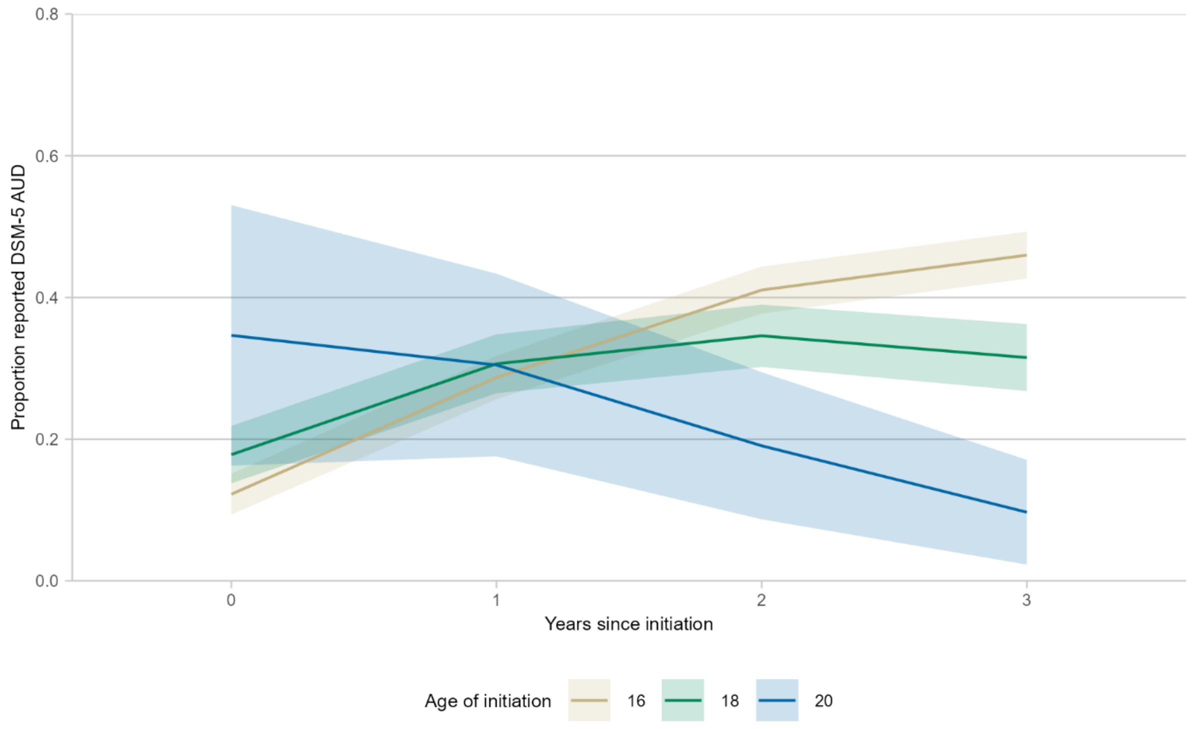

Figure F12 Trajectories of DSM-5 alcohol use disorder for all ages of initiation of whole drinks

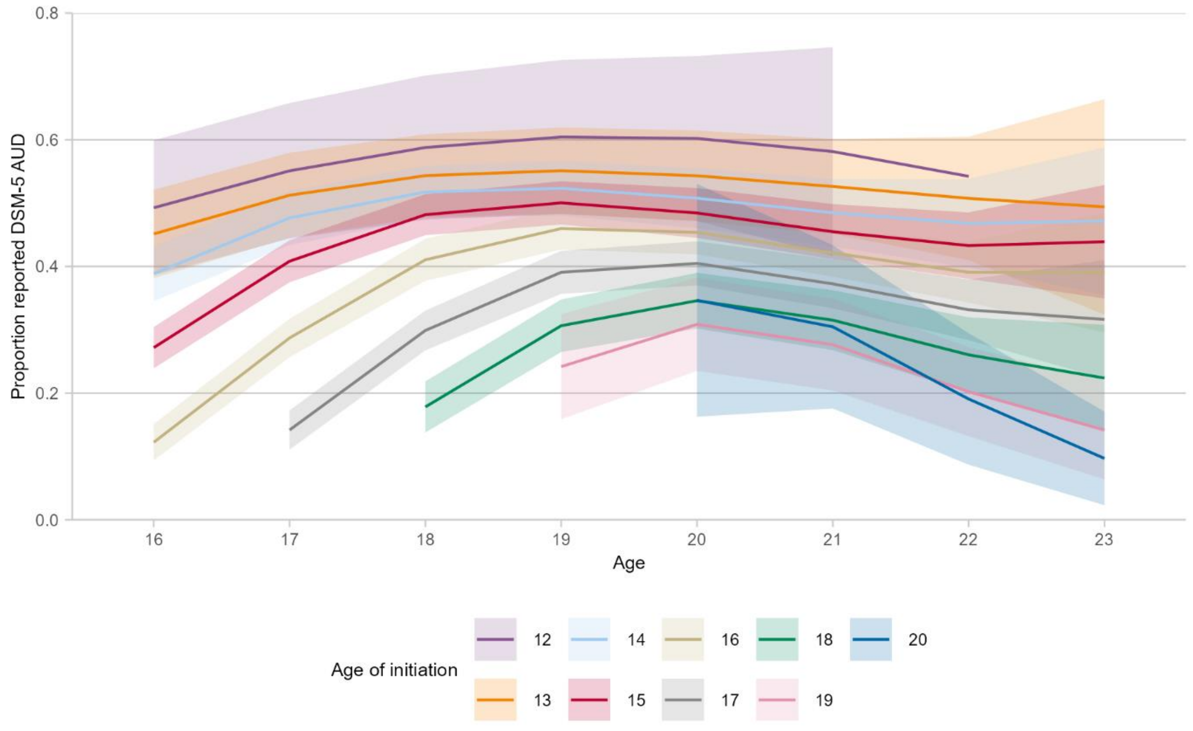

Model included linear and cubic terms for age; and linear, quadratic, and cubic terms for time (age + age^3 + time + $time^2$ + $time^3$). Further information on model fit is included in Appendix Table F1.

Table F9 Trajectories of DSM-5 alcohol use disorder – by age of initiation of whole drinks

		**Proportion reported symptoms consistent with DSM-5 alcohol use disorder (95% CI)**							
		**Age**							
		**16**	**17**	**18**	**19**	**20**	**21**	**22**	**23**
**Age of initiation**	12	49.3 (38.6, 59.9)	55.1 (44.4, 65.8)	58.8 (47.4, 70.1)	60.4 (48.3, 72.6)	60.2 (47.2, 73.2)	58.1 (41.7, 74.6)	54.2 (27.4, 81.0)	
	13	45.1 (38.2, 52.1)	51.2 (44.5, 57.9)	54.3 (47.8, 60.9)	55.1 (48.3, 61.9)	54.3 (47.1, 61.5)	52.6 (45.1, 60.1)	50.7 (41.0, 60.5)	49.4 (32.4, 66.4)
	14	38.8 (34.5, 43.2)	47.7 (43.4, 51.9)	51.7 (47.6, 55.9)	52.3 (48.0, 56.6)	50.8 (46.0, 55.5)	48.5 (43.2, 53.8)	46.8 (39.8, 53.8)	47.2 (35.5, 58.8)
	15	27.2 (23.9, 30.4)	40.8 (37.5, 44.1)	48.2 (44.9, 51.4)	50.0 (46.6, 53.5)	48.4 (44.5, 52.4)	45.5 (41.1, 49.8)	43.3 (38.0, 48.5)	43.9 (34.9, 52.9)
	16	12.2 (9.4, 15.1)	28.7 (25.7, 31.7)	41.0 (37.8, 44.3)	46.0 (42.7, 49.3)	45.4 (41.9, 48.8)	42.1 (38.4, 45.8)	39.1 (34.3, 43.9)	39.0 (29.6, 48.5)
	17		14.2 (11.1, 17.3)	29.9 (26.8, 33.0)	39.1 (35.7, 42.5)	40.5 (37.0, 44.0)	37.2 (33.4, 41.1)	33.2 (28.4, 37.9)	31.6 (22.3, 41.0)
	18			17.8 (13.8, 21.9)	30.6 (26.5, 34.8)	34.6 (30.2, 39.0)	31.5 (26.8, 36.2)	26.0 (20.1, 32.0)	22.4 (14.0, 30.8)
	19				24.2 (15.9, 32.5)	30.9 (23.5, 38.2)	27.7 (20.4, 34.9)	20.2 (13.2, 27.2)	14.2 (6.4, 21.9)
	20					34.7 (16.3, 53.0)	30.5 (17.6, 43.4)	19.1 (8.7, 29.4)	9.7 (2.3, 17.1)

Appendix G - Sensitivity analysis results

Figure G1 Trajectories of any heavy episodic drinking in the three years following initiation

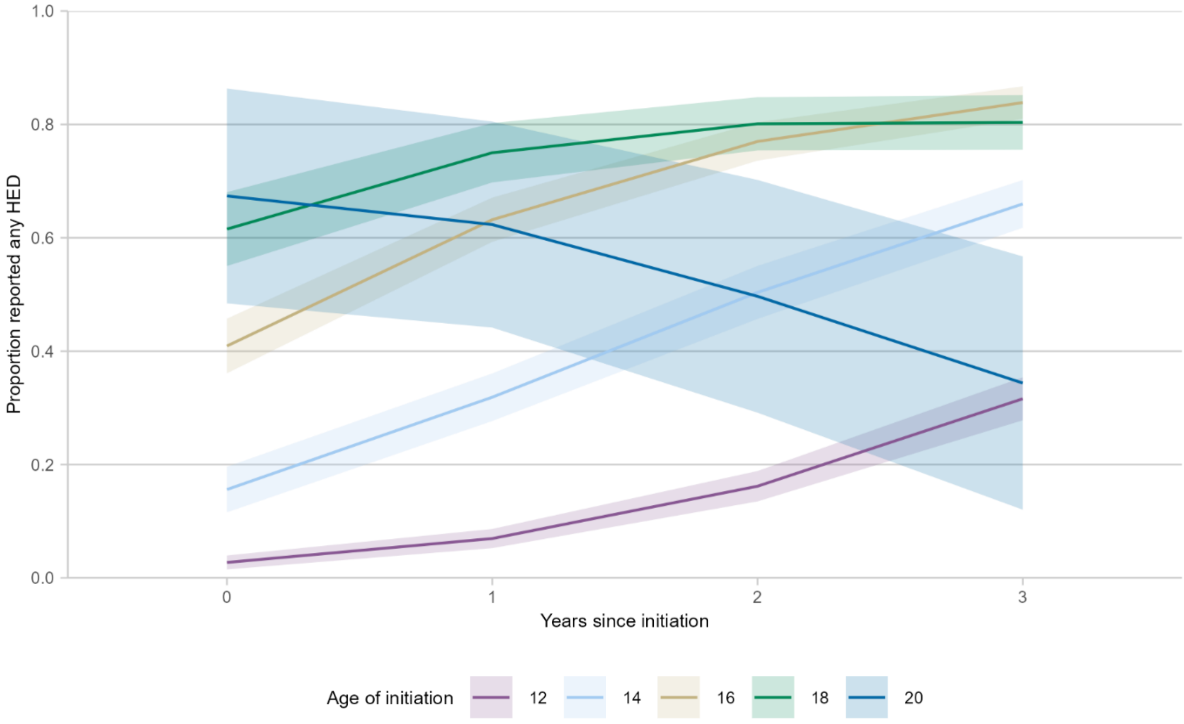

Figure G2 Trajectories of any heavy episodic drinking for all ages of initiation

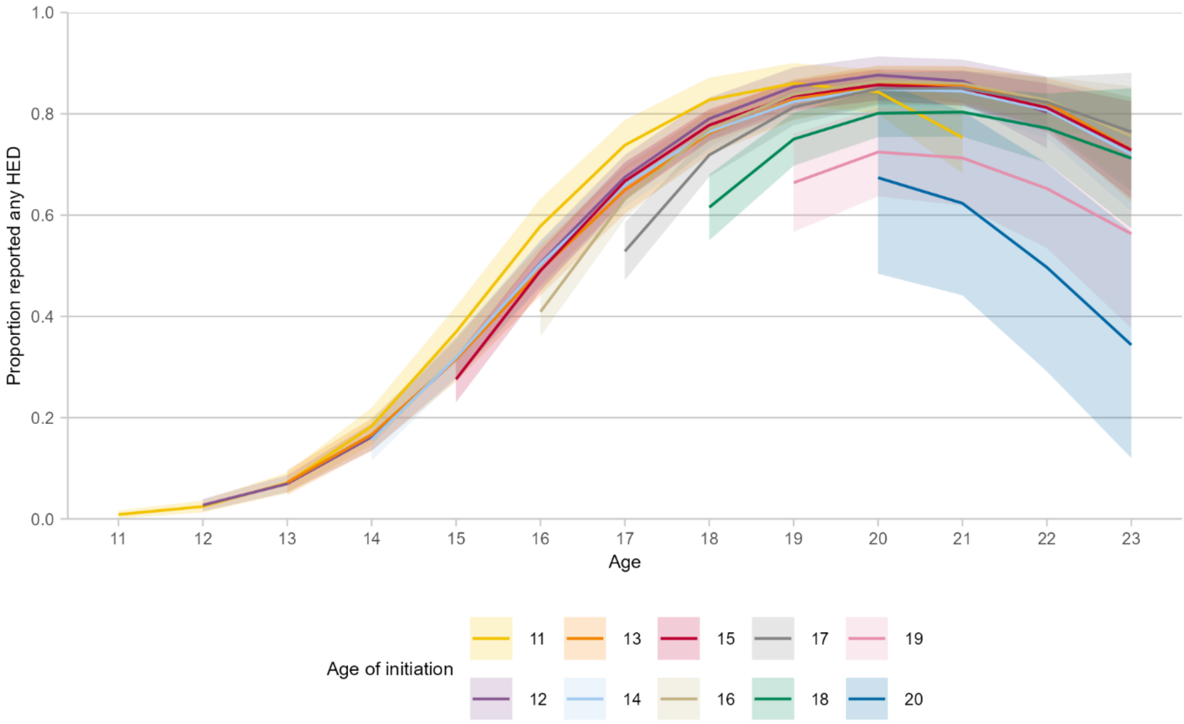

Model included linear and cubic terms for age; and linear, quadratic, and cubic terms for time (age + age^3 + time + $time^2$ + $time^3$).

Appendix G - Sensitivity analysis results

Table G1 Trajectories of any heavy episodic drinking – by age of initiation

	**z**	**Proportion reported any heavy episodic drinking (95% CI)**												
								**Age**						
		**11**	**12**	**13**	**14**	**15**	**16**	**17**	**18**	**19**	**20**	**21**	**22**	**23**
	11	0.9 (0.1, 1.6)	2.5 (1.3, 3.6)	7.1 (5.1, 9.2)	18.3 (14.7, 21.9)	36.9 (31.9, 41.9)	57.8 (52.5, 63.2)	73.8 (68.9, 78.8)	82.8 (78.4, 87.1)	86.0 (82.0, 90.0)	84.3 (80.0, 88.6)	75.3 (68.3, 82.3)		
	12		2.7 (1.5, 3.9)	6.9 (5.3, 8.6)	16.2 (13.5, 18.8)	31.6 (27.8, 35.4)	50.5 (46.2, 54.9)	67.5 (63.1, 71.9)	79.0 (74.9, 83.2)	85.3 (81.5, 89.2)	87.6 (84.0, 91.3)	86.4 (82.2, 90.7)	80.3 (73.2, 87.4)	
	13			7.2 (4.7, 9.7)	16.7 (13.5, 19.8)	31.5 (27.2, 35.9)	49.1 (44.2, 54.1)	64.9 (60.4, 69.5)	76.2 (71.9, 80.4)	82.8 (78.8, 86.8)	85.8 (82.0, 89.5)	85.8 (82.1, 89.4)	82.2 (77.4, 87.0)	72.9 (62.6, 83.2)
	14				15.6 (11.6, 19.6)	31.9 (27.7, 36.1)	50.4 (45.7, 55.0)	66.0 (61.8, 70.2)	76.5 (72.9, 80.0)	82.4 (79.1, 85.7)	84.9 (81.5, 88.2)	84.5 (80.7, 88.2)	80.7 (75.3, 86.2)	72.0 (60.8, 83.3)
**Age of initiation**	15					27.6 (23.1, 32.1)	49.0 (45.0, 53.0)	66.7 (63.0, 70.5)	77.8 (74.6, 80.9)	83.6 (80.7, 86.5)	85.7 (82.7, 88.8)	85.0 (81.6, 88.5)	81.2 (76.5, 86.0)	72.9 (63.3, 82.4)
	16						40.9 (36.1, 45.7)	63.2 (59.3, 67.1)	77.0 (73.6, 80.4)	83.9 (81.0, 86.7)	86.4 (83.5, 89.3)	86.0 (82.7, 89.3)	82.7 (78.1, 87.3)	75.6 (65.8, 85.4)
	17							52.9 (47.2, 58.5)	71.9 (67.6, 76.1)	81.3 (77.8, 84.9)	85.0 (81.8, 88.1)	85.1 (81.6, 88.5)	82.3 (77.4, 87.2)	76.4 (64.8, 88.1)
	18								61.5 (55.0, 68.0)	75.0 (69.8, 80.2)	80.1 (75.4, 84.8)	80.3 (75.5, 85.2)	77.2 (70.3, 84.0)	71.2 (57.4, 85.1)
	19									66.4 (56.7, 76.1)	72.4 (63.7, 81.2)	71.3 (61.9, 80.7)	65.2 (53.5, 77.0)	56.3 (37.7, 74.9)
	20										67.4 (48.4, 86.3)	62.3 (44.2, 80.5)	49.7 (29.2, 70.2)	34.4 (12.0, 56.7)

Figure G3 Trajectories of experience of any alcohol-related harm in the three years following initiation

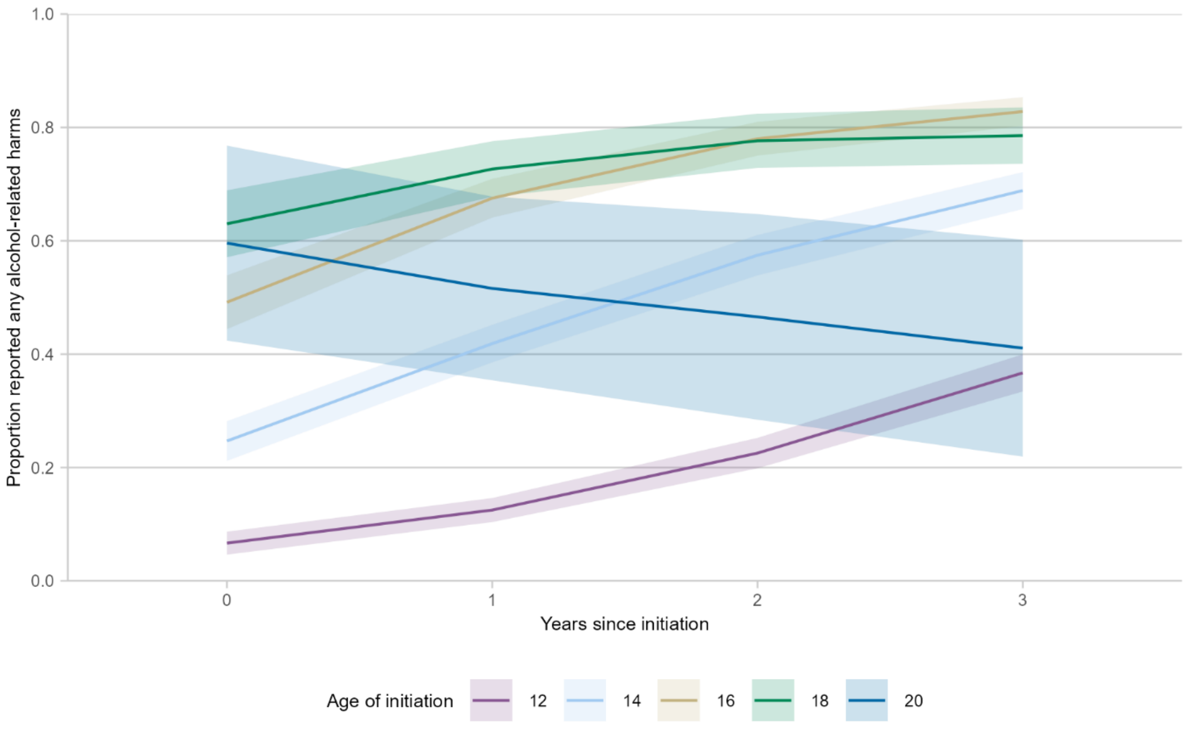

Figure G4 Trajectories of experience of any alcohol-related harm for all ages of initiation

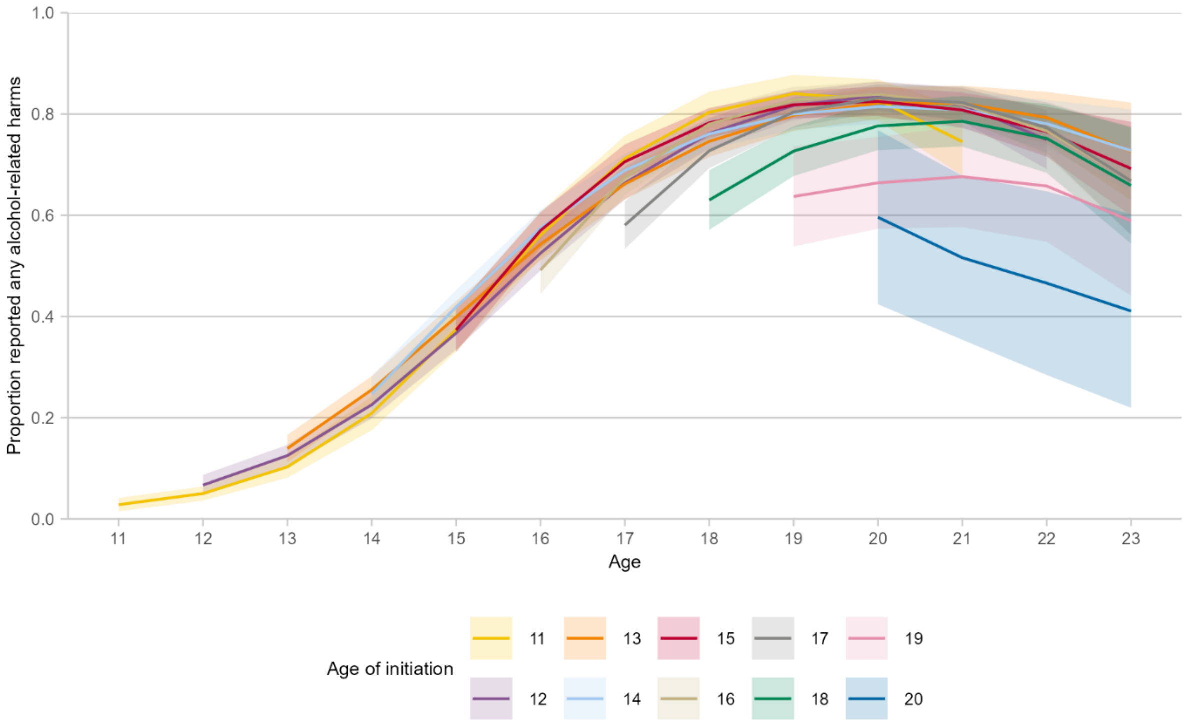

Model included linear and cubic terms for age; and linear, quadratic, and cubic terms for time (age + age^3 + time + $time^2$ + $time^3$).

Table G2 Trajectories of experience of any alcohol related harms – by age of initiation

		Proportion reported any alcohol-related harms (95% CI)												
		Age												
		**11**	**12**	**13**	**14**	**15**	**16**	**17**	**18**	**19**	**20**	**21**	**22**	**23**
**Age of initiation**	11	2.8 (1.5, 4.1)	5.0 (3.6, 6.4)	10.3 (8.1, 12.4)	20.9 (17.5, 24.2)	37.4 (33.1, 41.7)	56.1 (51.4, 60.7)	71.2 (66.7, 75.7)	80.3 (76.3, 84.4)	84.0 (80.3, 87.7)	82.8 (78.7, 86.8)	74.5 (67.8, 81.2)		
	12		6.7 (4.7, 8.7)	12.5 (10.4, 14.6)	22.5 (19.9, 25.2)	36.7 (33.4, 40.0)	52.5 (49.1, 55.9)	66.4 (63.1, 69.6)	76.2 (73.1, 79.2)	81.7 (78.8, 84.6)	83.6 (80.8, 86.4)	81.9 (78.6, 85.2)	74.9 (69.1, 80.7)	
	13			13.9 (11.2, 16.6)	25.5 (22.8, 28.2)	39.9 (36.8, 43.0)	54.3 (51.1, 57.5)	66.2 (63.1, 69.2)	74.6 (71.6, 77.6)	79.8 (76.7, 82.8)	82.2 (79.0, 85.3)	82.1 (78.6, 85.6)	79.3 (74.2, 84.4)	72.6 (63.0, 82.2)
	14				24.7 (21.2, 28.2)	41.9 (38.6, 45.1)	57.5 (53.9, 61.0)	68.9 (65.6, 72.1)	76.1 (73.1, 79.0)	80.0 (76.9, 83.1)	81.5 (78.1, 84.8)	80.8 (77.2, 84.5)	78.1 (73.4, 82.8)	72.8 (64.6, 80.9)
	15					37.3 (33.0, 41.7)	57.0 (53.4, 60.6)	70.6 (67.2, 74.0)	78.3 (75.3, 81.2)	81.8 (79.1, 84.5)	82.5 (79.6, 85.4)	80.8 (77.3, 84.2)	76.5 (71.6, 81.4)	69.2 (60.0, 78.5)
	16						49.2 (44.4, 53.9)	67.5 (64.1, 70.9)	78.0 (75.0, 81.0)	82.8 (80.3, 85.3)	83.9 (81.4, 86.4)	82.0 (79.0, 84.9)	76.6 (71.8, 81.4)	66.9 (56.2, 77.6)
	17							58.0 (53.4, 62.7)	72.7 (69.3, 76.1)	80.4 (77.4, 83.3)	83.1 (80.4, 85.9)	82.2 (79.0, 85.5)	77.3 (72.3, 82.3)	66.8 (56.1, 77.5)
	18								63.0 (57.1, 68.9)	72.7 (67.8, 77.6)	77.6 (72.9, 82.4)	78.6 (73.6, 83.5)	75.2 (68.4, 82.0)	65.9 (54.4, 77.3)
	19									63.7 (53.8, 73.6)	66.4 (57.3, 75.4)	67.6 (57.7, 77.6)	65.8 (54.8, 76.8)	58.8 (44.2, 73.5)
	20										59.6 (42.4, 76.8)	51.6 (35.4, 67.8)	46.6 (28.4, 64.7)	41.1 (21.9, 60.2)

Appendix G - Sensitivity analysis results

Figure G5 Trajectories of any heavy episodic drinking in the three years following initiation of whole drinks

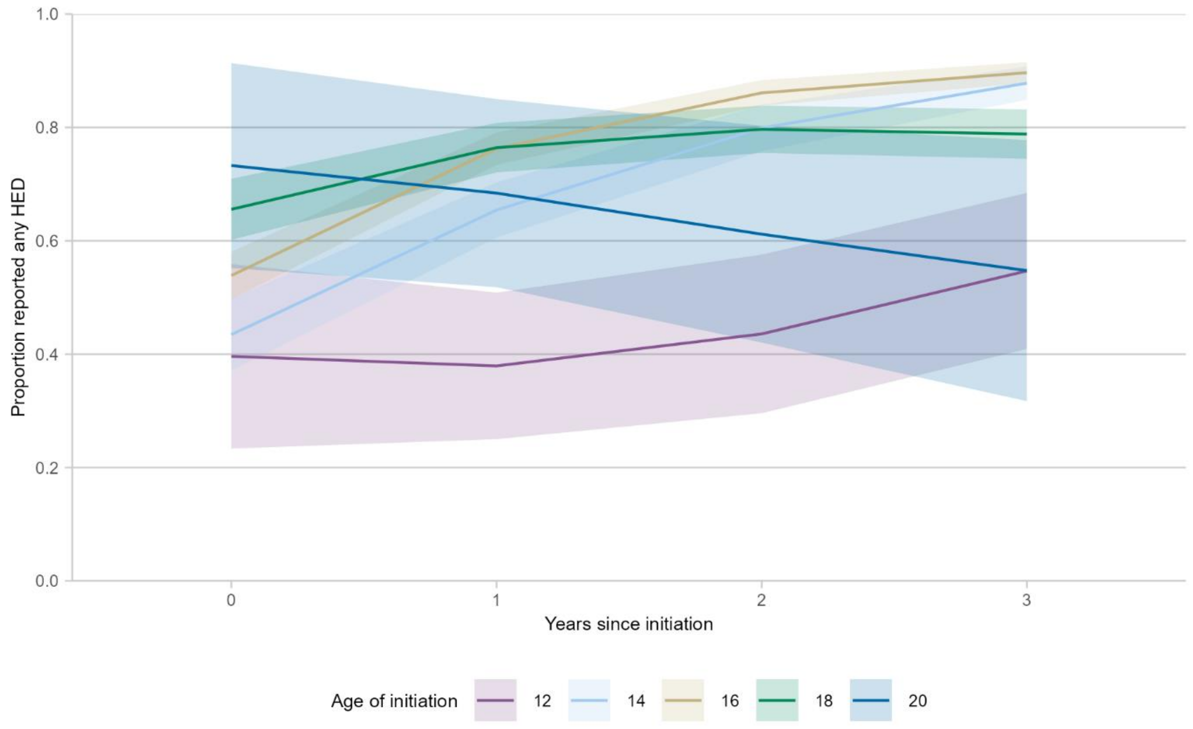

Figure G6 Trajectories of any heavy episodic drinking for all ages of initiation of whole drinks

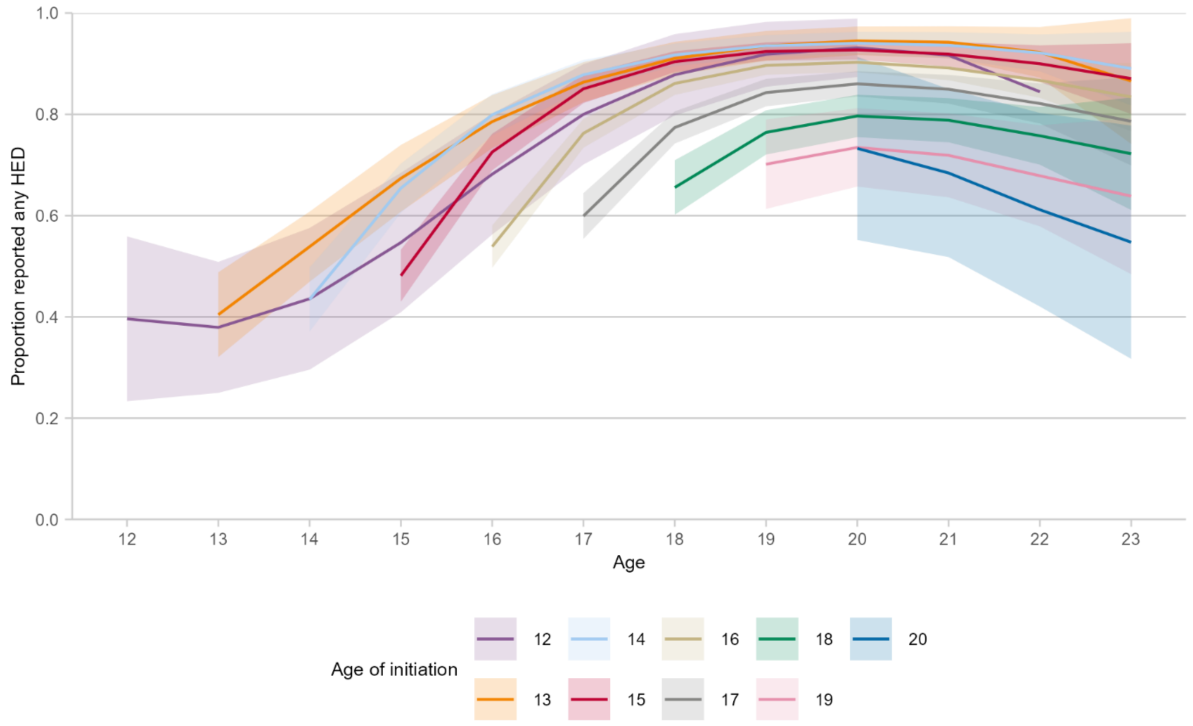

Model included linear and cubic terms for age; and linear, quadratic, and cubic terms for time (age + age^3 + time + $time^2$ + $time^3$).

Table G3 Trajectories of any heavy episodic drinking – by age of initiation of whole drinks

		Proportion reported any heavy episodic drinking (95% CI)											
		**Age**											
		**12**	**13**	**14**	**15**	**16**	**17**	**18**	**19**	**20**	**21**	**22**	**23**
	12	39.6 (23.3, 55.9)	37.9 (25.0, 50.8)	43.6 (29.6, 57.5)	54.7 (40.9, 68.4)	68.2 (56.3, 80.1)	80.0 (70.1, 89.9)	87.8 (79.8, 95.8)	91.9 (85.5, 98.2)	93.1 (87.4, 98.9)	91.7 (83.3, 100.0)	84.5 (61.9, 107.0)	
	13		40.4 (32.1, 48.8)	53.9 (47.0, 60.8)	67.4 (60.8, 73.9)	78.6 (73.3, 83.8)	86.3 (82.4, 90.3)	91.0 (87.8, 94.3)	93.5 (90.6, 96.5)	94.5 (91.7, 97.3)	94.2 (91.1, 97.4)	92.2 (87.3, 97.2)	86.6 (74.2, 99.0)
	14			43.4 (37.1, 49.8)	65.4 (60.6, 70.3)	79.9 (75.8, 84.0)	87.8 (84.9, 90.7)	91.7 (89.5, 94.0)	93.5 (91.4, 95.6)	94.1 (91.8, 96.3)	93.6 (91.0, 96.2)	92.1 (88.5, 95.8)	89.0 (81.8, 96.3)
	15				48.1 (43.0, 53.3)	72.6 (68.9, 76.2)	85.0 (82.2, 87.9)	90.4 (88.3, 92.5)	92.4 (90.6, 94.2)	92.7 (90.8, 94.7)	91.9 (89.5, 94.3)	90.0 (86.4, 93.6)	87.1 (80.1, 94.0)
**Age of initiation**	16					53.9 (49.7, 58.0)	76.3 (73.4, 79.1)	86.1 (83.8, 88.3)	89.7 (87.8, 91.5)	90.3 (88.3, 92.2)	89.2 (86.7, 91.6)	86.7 (83.3, 90.1)	83.4 (76.7, 90.2)
	17						59.9 (55.4, 64.4)	77.4 (74.2, 80.6)	84.3 (81.6, 87.0)	86.0 (83.5, 88.5)	85.0 (82.1, 87.8)	82.1 (78.2, 86.1)	78.6 (69.9, 87.3)
	18							65.5 (60.2, 70.9)	76.4 (72.1, 80.8)	79.7 (75.5, 83.8)	78.8 (74.5, 83.2)	75.8 (70.1, 81.5)	72.2 (61.1, 83.3)
	19								70.1 (61.3, 79.0)	73.5 (65.8, 81.2)	71.9 (63.6, 80.2)	67.9 (57.9, 77.8)	63.8 (48.5, 79.2)
	20									73.3 (55.2, 91.3)	68.4 (51.8, 85.0)	61.2 (42.1, 80.3)	54.7 (31.7, 77.8)

Figure G7 Trajectories of experience of any alcohol-related harm in the three years following initiation of whole drinks

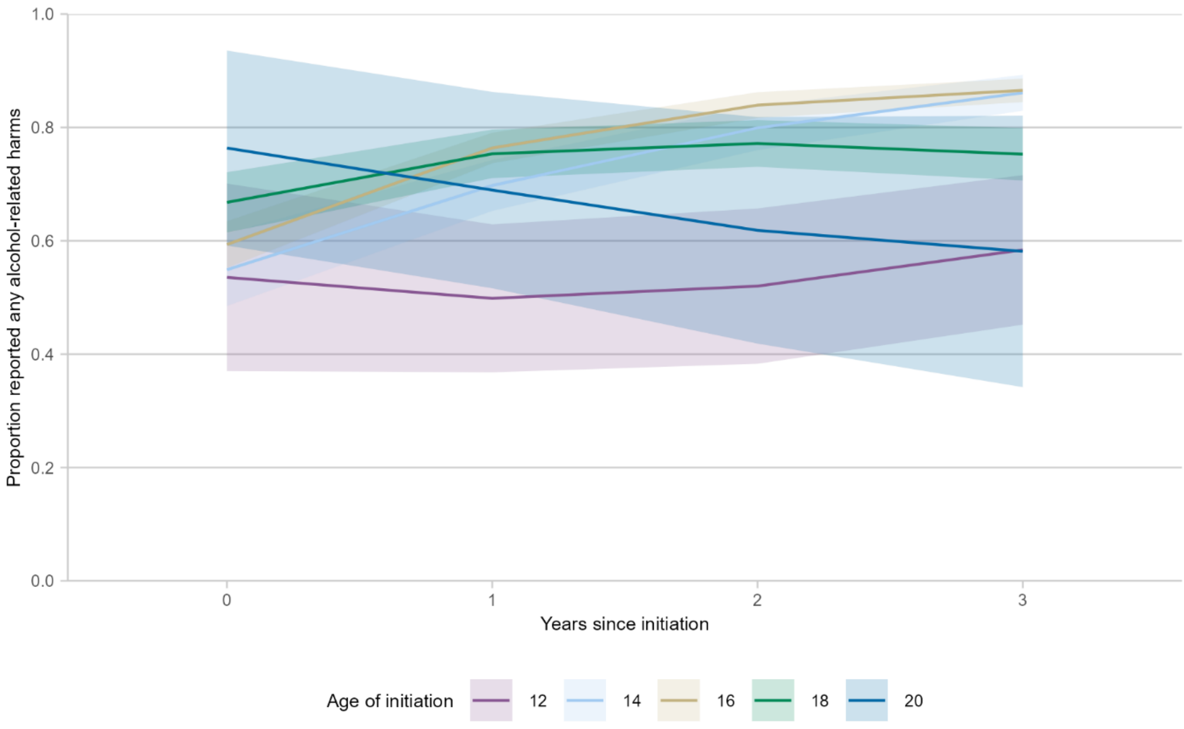

Figure G8 Trajectories of experience of any alcohol-related harm for all ages of initiation of whole drinks

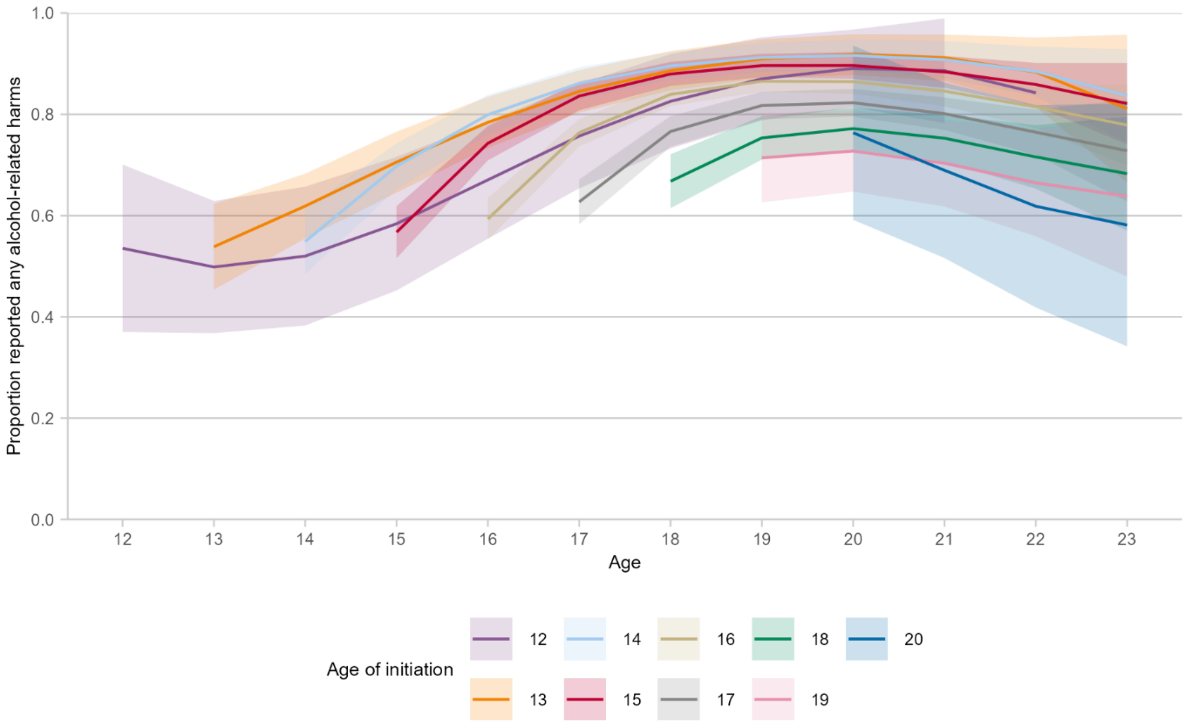

Model included linear, quadratic, and cubic terms for age; and linear, quadratic, and cubic terms for time (age + age^2 +age^3 + time + $time^2$ + $time^3$).

Table G4 Trajectories of experience of any alcohol related harms – by age of initiation of whole drinks

		Proportion reported any alcohol-related harms (95% CI)											
		Age											
		**12**	**13**	**14**	**15**	**16**	**17**	**18**	**19**	**20**	**21**	**22**	**23**
**Age of initiation**	12	53.5 (37.0, 70.1)	49.8 (36.8, 62.9)	52.0 (38.3, 65.7)	58.4 (45.2, 71.6)	67.1 (55.5, 78.6)	75.7 (65.4, 86.0)	82.6 (73.3, 91.9)	87.0 (78.8, 95.2)	89.1 (81.4, 96.7)	88.6 (78.3, 98.9)	84.3 (62.2, 106.4)	
	13		53.8 (45.4, 62.3)	61.9 (55.5, 68.3)	70.6 (64.6, 76.6)	78.4 (73.4, 83.5)	84.5 (80.3, 88.7)	88.6 (84.8, 92.5)	91.0 (87.1, 94.8)	91.8 (87.8, 95.8)	91.2 (86.6, 95.8)	88.4 (81.5, 95.2)	81.1 (66.6, 95.7)
	14			54.9 (48.5, 61.3)	69.8 (65.3, 74.2)	79.9 (76.0, 83.8)	86.1 (83.0, 89.3)	89.6 (86.8, 92.3)	91.2 (88.4, 94.1)	91.6 (88.5, 94.7)	90.8 (87.2, 94.4)	88.5 (83.6, 93.3)	83.7 (74.5, 92.8)
	15				56.7 (51.6, 61.8)	74.3 (70.9, 77.6)	83.6 (80.8, 86.4)	88.0 (85.6, 90.3)	89.6 (87.3, 91.9)	89.6 (87.0, 92.3)	88.4 (85.2, 91.5)	85.9 (81.6, 90.2)	82.1 (74.1, 90.1)
	16					59.3 (55.2, 63.4)	76.4 (73.7, 79.0)	83.9 (81.7, 86.2)	86.5 (84.5, 88.6)	86.5 (84.1, 88.8)	84.6 (81.6, 87.5)	81.5 (77.4, 85.6)	77.9 (69.9, 85.8)
	17						62.7 (58.3, 67.1)	76.6 (73.6, 79.6)	81.7 (79.0, 84.5)	82.3 (79.6, 85.0)	80.1 (77.0, 83.3)	76.5 (72.0, 80.9)	72.8 (62.8, 82.8)
	18							66.8 (61.5, 72.1)	75.3 (71.1, 79.6)	77.2 (73.1, 81.3)	75.3 (70.7, 79.9)	71.6 (65.3, 77.8)	68.3 (57.1, 79.5)
	19								71.4 (62.6, 80.2)	72.7 (64.8, 80.7)	70.3 (61.8, 78.8)	66.5 (56.0, 77.0)	63.8 (48.0, 79.6)
	20									76.4 (59.2, 93.5)	68.9 (51.6, 86.3)	61.8 (41.9, 81.8)	58.1 (34.2, 82.1)
